# An Overview on Antimalarial Peptides: Natural Sources, Synthetic Methodology and Biological Properties

**DOI:** 10.3390/molecules28237778

**Published:** 2023-11-25

**Authors:** Nety Kurniaty, Rani Maharani, Ace Tatang Hidayat, Unang Supratman

**Affiliations:** 1 Department of Pharmacy, Faculty of Mathematics and Natural Sciences, Universitas Islam Bandung, Jl. Tamansari No.1, Tamansari, Kec. Bandung Wetan, Kota Bandung 40116, West Java, Indonesia; 2 Department of Chemistry, Faculty of Mathematics and Natural Sciences, Universitas Padjadjaran, Sumedang 45363, Indonesia; ace.hidayat@unpad.ac.id (A.T.H.); unang.supratman@unpad.ac.id (U.S.); 3 Laboratorium Sentral, Universitas Padjadjaran, Sumedang 45363, Indonesia; 4 Centre of Natural Products and Synthesis Studies, Faculty of Mathematics and Natural Sciences, Universitas Padjadjaran, Sumedang 45363, Indonesia

**Keywords:** peptide, antimalarial activity, isolation, synthesis peptide

## Abstract

Peptide compounds play a significant role in medicinal chemistry as they can inhibit the activity of species that cause malaria. This literature review summarizes the isolation of antimalarial peptides, the synthesis method with the detailed structure and sequences of each peptide, and discusses the biological activity of the isolated and synthesized compounds. The synthetic routes and reactions for cyclic and linear antimalarial peptides are systematically highlighted in this review including preparing building blocks, protection and deprotection, coupling and cyclization reactions until the target compound is obtained. Based on the literature data and the results, this review’s aim is to provide information to discover and synthesize more antimalarial peptide for future research.

## 1. Introduction

Malaria is a common disease in Africa and certain Asian countries and is one of the leading causes of death worldwide. Indeed, the World Health Organization (WHO) reported that the number of deaths increased by 10% in 2020 compared with 2019, but declined to an estimated 619,000 deaths in 2021. Furthermore, nearly half of the world’s population is at risk of malaria [1].

Malaria is caused by a tiny protozoon belonging to the *Plasmodium* species that is spread to humans through the bite of an infected female *Anopheles* mosquito. Of the 172 *Plasmodium* species, five can infect humans, including *Plasmodium malariae*, *P. falciparum*, *P. vivax*, *P. ovale*, and *P. knowlesi* [2]. Many drugs have been produced to treat and prevent this disease. For example, several peptides have been identified as possessing antimalaria properties against *P. falciparum*. These antimalarial peptides were initially isolated from plants or animals and the low yields have stimulated great advances in the development of the synthetic methodologies and synthetic analogs. This review will discuss the relevant literature published between 2015 and 2023 regarding the isolation techniques, chemical synthesis, and biological activity of the antimalarial peptides.

## 2. Isolation of Antimalarial Peptide

**Figure 1:**
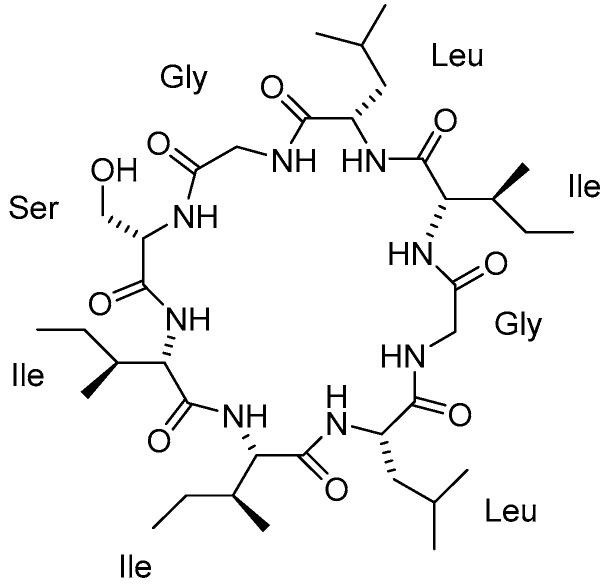
Structure of ribifolin.

Nature has become a source of antimalarial peptides. Various types of peptides with antimalarial activity have been isolated with cyclic and linear structures (Table 1). Peptides with antimalarial activity are commonly found as cyclopeptides; for example, ribifolin is a cyclopeptide containing eight amino acid residues (ILGSIILG) that has antimalarial activity (Figure 1) and was first isolated by Pinto et al. in 2015 from the aerial parts of *Jatropha ribifolia*. The peptide was extracted using Strata C18-E solid-phase extraction catridge using H_2_O and CH_3_CN as mobile phases (0% 30%, 70%, and 100% CH_3_CN in H_2_O), then purified by semipreparative HPLC using a linear gradient of 40−80% of CH_3_CN, containing 0.036% TFA in H_2_O containing 0.045% TFA for 120 min [3].

**Figure 2:**
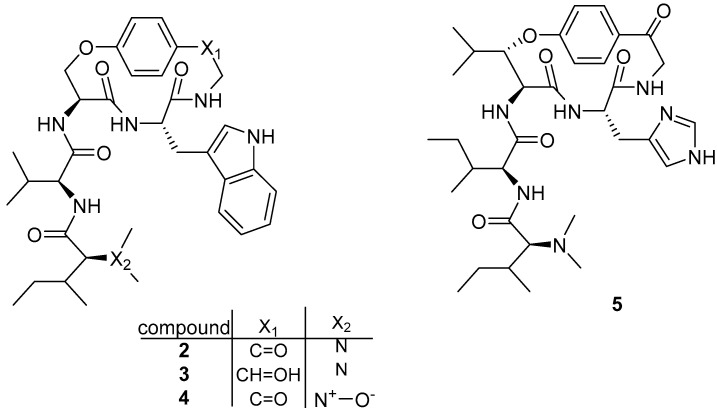
Structure of cyclopeptide alkaloids isolated from *Hymenocardia acida*.

Antimalarial cyclopeptides can also be isolated from alkaloid-producing plants, such as the cyclopeptide alkaloids hymenocardine (**2**), hymenocardinol (**3**), hymenocardine *N*-oxide (**4**), and hymenocardine-H (**5**) (Figure 2) extracted from the root bark of *Hymenocardia acida* with 80% methanol by Tuenter et al. in 2016 [4]. The crude extract was fractioned by liquid–liquid partitioning followed by flash chromatography, and then the compounds were purified by semipreparative HPLC with DAD and ESIMS detection.

**Figure 3:**
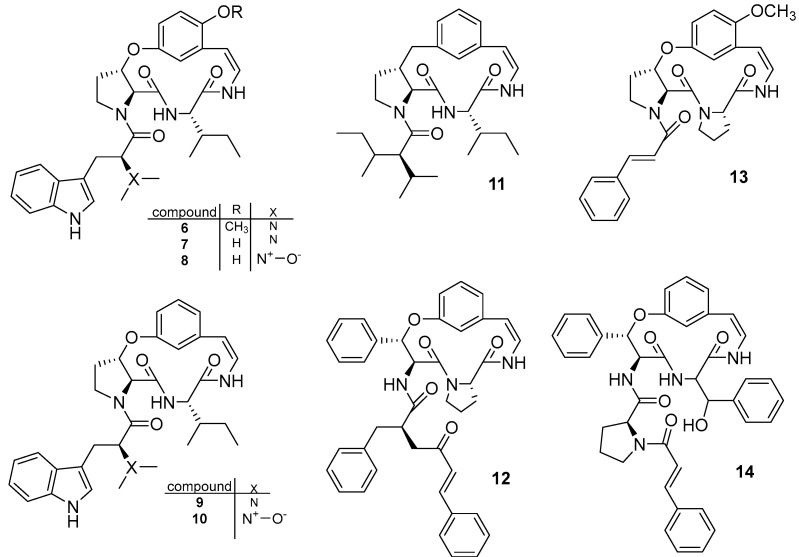
Structure of cyclopeptide alkaloids isolated from *Ziziphus oxyphylla*.

Tuenter et al. (2016) also isolated nine cyclopeptide alkaloids from two different batches of the roots of *Ziziphus oxyphylla*. The first batch was subjected to a general liquid−liquid partition and fractionation scheme, whereas a more alkaloid-specific fractionation procedure was used for the second batch to increase the number of alkaloids detected. The single compounds were purified by semipreparative HPLC with DAD and ESIMS detection with LC-DAD-SPE-NMR and identified as nummularine-R (**6**), *O*-desmethylnummularine-R (**7**), O-desmethylnummularine-R *N*-oxide (**8**), hemsine-A (**9**), hemsine-A *N*-oxide (**10**), ramosine-A (**11**), oxyphylline-C (**12**), oxyphylline-E (**13**), and oxyphylline-F (**14**) (Figure 3) [5].

Maluf et al. (2016) isolated a 42-amino acid polypeptide (YKCHKKGGHCFPKEKICLPPSSDFGKMDCRWRWKCCKKGSG, MW = 4889.81 Da) named crotamine (**15**) from rattlesnake venom [6]. Six hundred milligrams of crude dried venom was dissolved in 5 mL of 0.25 M ammonium formate buffer (pH 3.5) and the major component crotoxin was eliminated by slow-speed centrifugation before crotamine was recovered in a narrow protein peak by raising the NaCl concentration and acid hydrolysis. The purity of crotamine was further confirmed by analytical RP-HPLC with a C18-column, LCMS, and ESI-MS.

**Figure 4:**
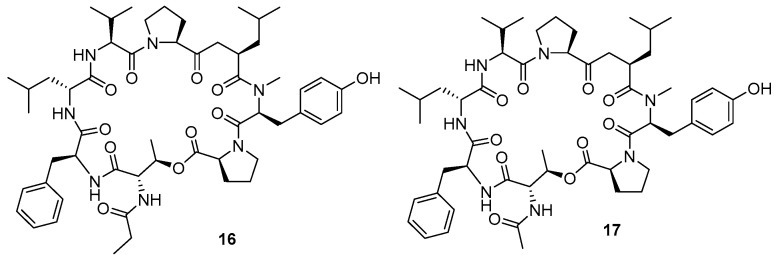
Structure of cyclic octadepsipeptides isolated from *Streptomyces* sp. RK85-270.

Two new cyclic octadepsipeptides octaminomycin A (**16**) and octaminomycin B (Figure 4) (**17**) discovered by Jang et al. (2017) exhibited activity against *P. falciparum*. The octadepsipeptides were isolated from a microbial metabolite fraction library of Streptomyces sp. RK85-270 based on Natural Products Plot (NPPlot) screening using liquid chromatography for extraction and purification [7].

**Figure 5:**
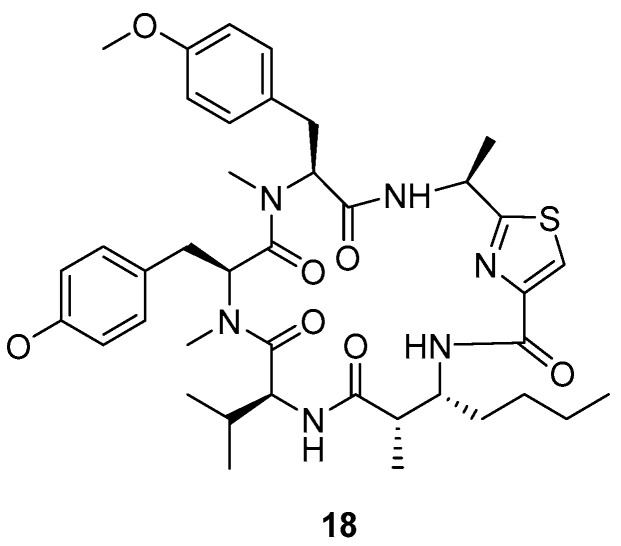
Structure of kakeromide B.

Sweeney–Jones et al. (2020) isolated an antimalarial cyclopeptide named kakeromamide B (**18**) (Figure 5) from Fijian marine cynobacterium by VLC using 80 mL of silica gel in a glass vacuum funnel [8]. The fractions were collected using a stepped gradient of hexane, EtOAc, and MeOH (100% hexane, 10% EtOAc/90% hexane, 20% EtOAc/80% hexane, 40% EtOAc/60% hexane, 60% EtOAc/40% hexane, 80% EtOAc/20% hexane, 100% EtOAc, 25% MeOH/75% EtOAc, and 100% MeOH), then separated by reverse SPE phase (gradient decreasing polarity gradually starting with 15% MeOH in H_2_O to 100% MeOH). Fractions 3, 4, and 5 were separated again by HPLC using a C18-silica column to obtain kakeromamide B (tR = 17.5 min, 0.18 mg).

**Figure 6:**
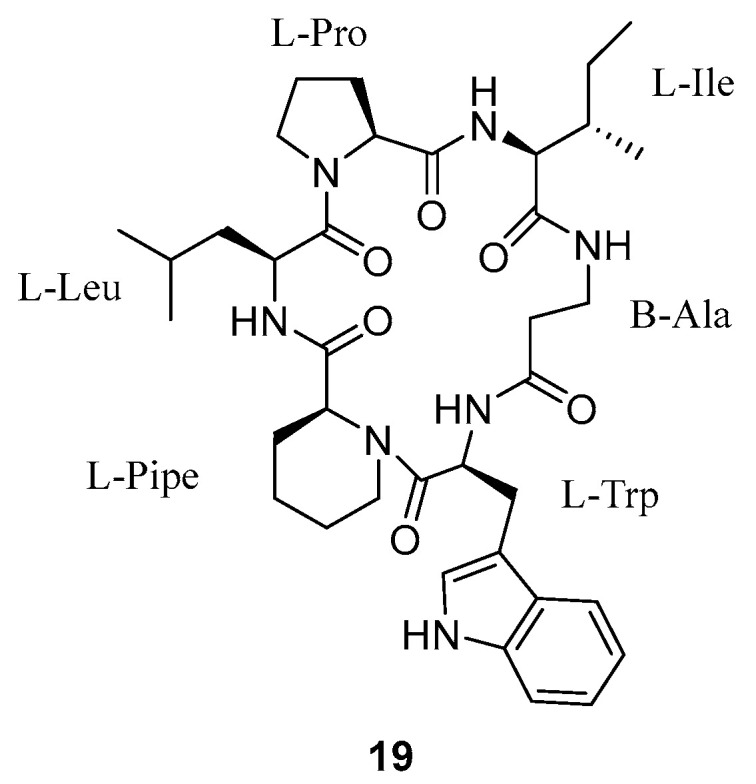
Structure of pipecolisporin.

Another cyclic peptide with antimalarial activity called pipecolisporin (**19**) (Figure 6) was isolated in 2021 by Fernández-Pastor et al. from the endophytic fungus Nigrospora oryzae CF-298113 [9]. A scaled-up culture of Nigrospora oryzae CF-298113 was extracted using methyl ethyl ketone (MEK) in BRFT medium and then purified using reversed-phase C18 medium pressure chromatography followed by semi-preparative reversed-phase HPLC on a phenyl column applying isocratic elution of H_2_O-CH_3_CN as the mobile phase (3.6 mL/min; 32% CH_3_CN for 40 min; UV detection at 210 nm). Pipecolisporin (**19**) was obtained as an amorphous white powder.

**Figure 7:**
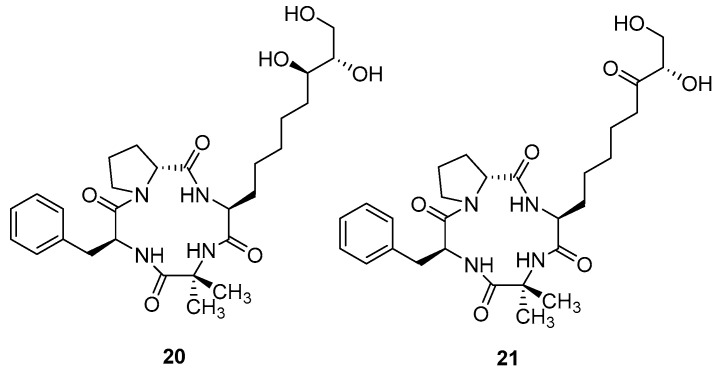
Structure of koshidacin A and B.

Subsequently, the antiplasmodial peptides kosidachin A (**20**) and kosidachin B (**21**) (Figure 7) were discovered by Watanabe et al. (2022) [10]. The compounds are cyclic tetrapeptides isolated from a culture broth of Pochonia binensis FKR-0564 by HPLC using Capcell Pak C18 column with an isocratic solvent system. Two new compounds were acquired from two fractions with retention times between 12.5–14.0 and 15.0–16.5 min.

**Figure 8:**
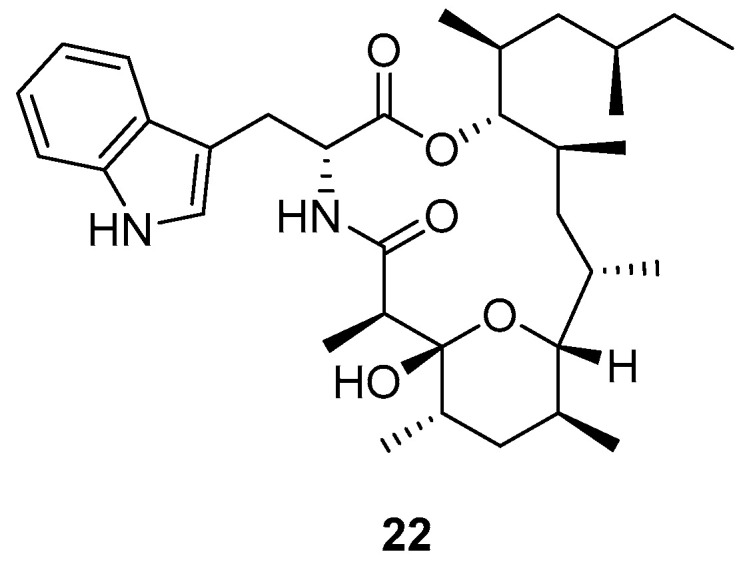
Structure of georatusin.

Another antimalarial peptide georatusin (**22**) (Figure 8) was isolated by Shi et al. (2018) from Geomyces auratus, which is a mesophilic fungus [11]. The ethyl extract was fractionated using a flash purification system with a gradient elution of n-hexane:acetone and purified by semipreparative HPLC on a RP-HPLC with C-18 to obtain georatusin.

**Figure 9:**
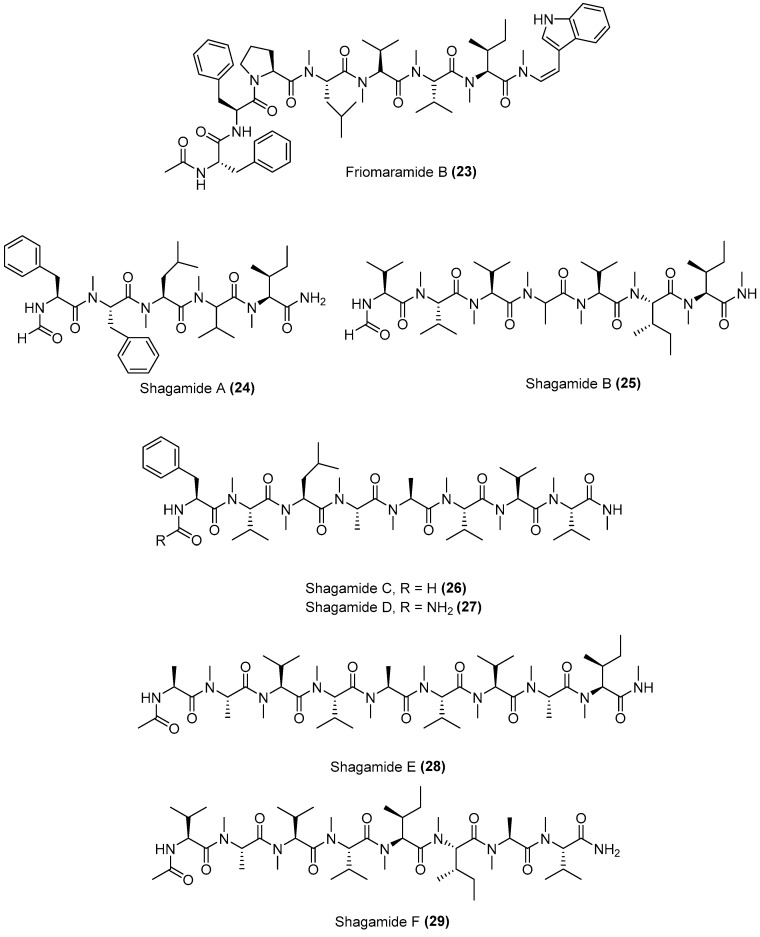
Structure of linear antimalarial peptides isolated from Inflatella coelosphaeroide.

Some linear peptides have also been reported to be active against malaria parasite. *N*-methylated linear peptides friomaramide B and shagamides A-F were purified from the marine sponge Inflatella coelosphaeroide by Bracegirdle et al. in 2022 (Figure 9) [12]. A collection of I. coelosphaeroides sponge samples was extracted, mounted onto silica gel and then partitioned using NP flash MPLC before fractionation by semipreparative C18 HPLC using a linear gradient. The individual fractions were then purified again by C18 HPLC to obtain friomaramide B (**23**), shagamide A (**24**), shagamide B (**25**), shagamide C (**26**), shagamide D (**27**), shagamide E (**28**), and shagamide F (**29**).

**Figure 10:**
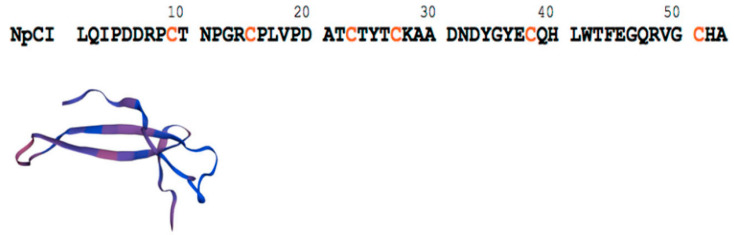
The derived putative 53-residue full protein sequence of NpCI and modelled folded of NpCI. The presence of six-cysteine (C) residues are highlighted in red.

Recently, Cabrera-Muñoz et al. (2023) reported that the carboxypeptidase NpCI (**30**) inhibited *P. falciparum* growth (Figure 10) [13]. The compound was isolated from the mollusk *Nerita versicolor* and purified using a CPA-ehtyloxy-Sepharose CL-6B matrix. Figure 10 shows the sequence of NpCI and modelled folded of NpCI displaying a central compact region, a long *N*-tail, and a short C-tail.

## 3. Synthesis of Antimalarial Peptides

Cyclic and linear peptides have been synthesized by various methods as outlined in the following section.

### 3.1. Cyclopeptides

#### 3.1.1. Mahafacyclin B

**Scheme 1:**
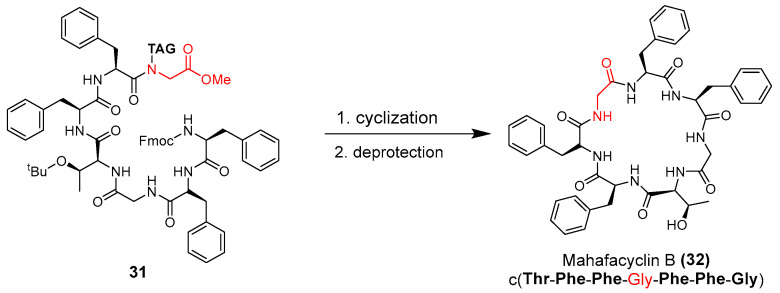
Synthesis of mahafacyclin B (**32**). Hydrophobically tagged Gly-OMe in compound **31** is highlighted in red. The untagged-glycine residue in compound **32** is highlighted in red.

The antimalarial mahafacyclin B (**32**) is a cyclic heptapeptide that was synthesized by Fujita et al. (2013) using the soluble-tag assisted liquid–phase method [14]. This method allowed peptide head-to-tail cyclization leading to the total synthesis of mahafacyclin B assisted by hydrophobic benzyl alcohol. The synthesis was initiated by reductive amination of hydrophobic benzaldehydes using amino acid methyl esters to obtain hydrophobically tagged Gly-Ome from its reaction with coupling reagent Fmoc-Phe-OH, and a combination of HATU, HOAt, DIPEA, and THF. Basic deprotection of the hydrophobically tagged dipeptide produces diketopiperazine when the C-terminal residue is proline. The hydrophobically tagged tripeptide was deprotected to obtain the cyclized compound (Scheme 1). A less polar solvent was employed during cyclization to overcome the issue of the highly hydrophobic residues in the linear precursor. The amide nitrogen was used as the marking site allowing the rapid reaction checks and product isolation.

#### 3.1.2. Aerucylamide B

**Scheme 2:**
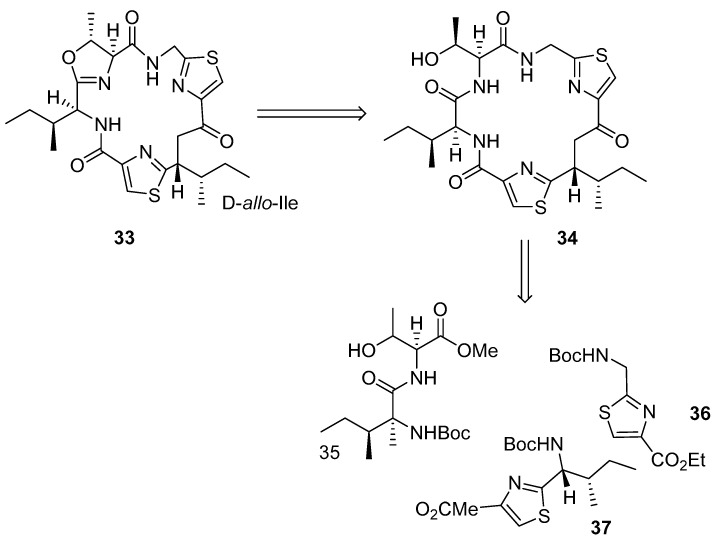
Retrosynthesis analysis of **33**.

**Scheme 3:**
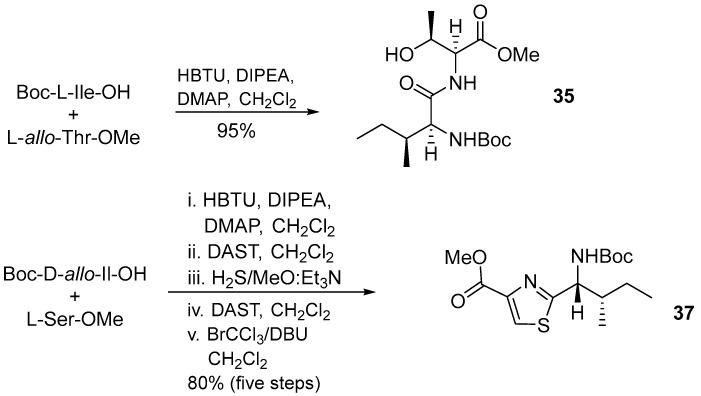
Synthesis of building blocks **35** and **37**.

Aerucyclamide B (**33**) was synthesized by Peña et al. (2013) using two heterocycles and a dipeptide as building blocks (Scheme 2) [15]. Dipeptide **34** and thiazole **35** were synthesized in the first step and then HBTU was used as coupling reagent to produce the thiazole precursor **37** through cyclodehydration of β-hydroxythioamides and further oxidation methodology (Scheme 3). 

**Scheme 4:**
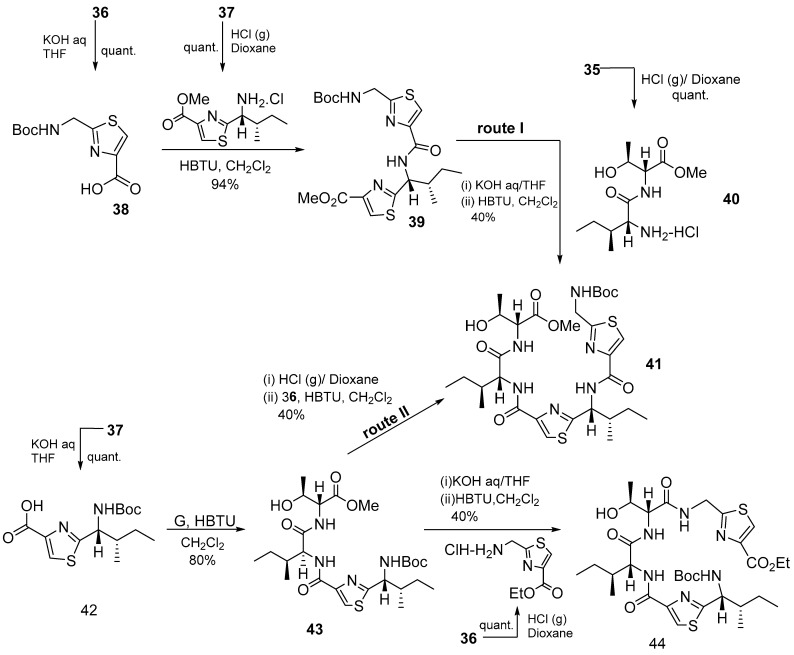
Synthesis of bis- and tris-heterocycles.

In the next step, two intermediate compounds **41** and **44** were prepared to obtain macrocycle **34**. Compound **41** was synthesized via routes I and II (Scheme 4). Route I involved a coupling between the *N*-deprotected dipeptide **40** and intermediate **39**, while route II involved coupling between compound **42** and **43**. 

**Scheme 5:**
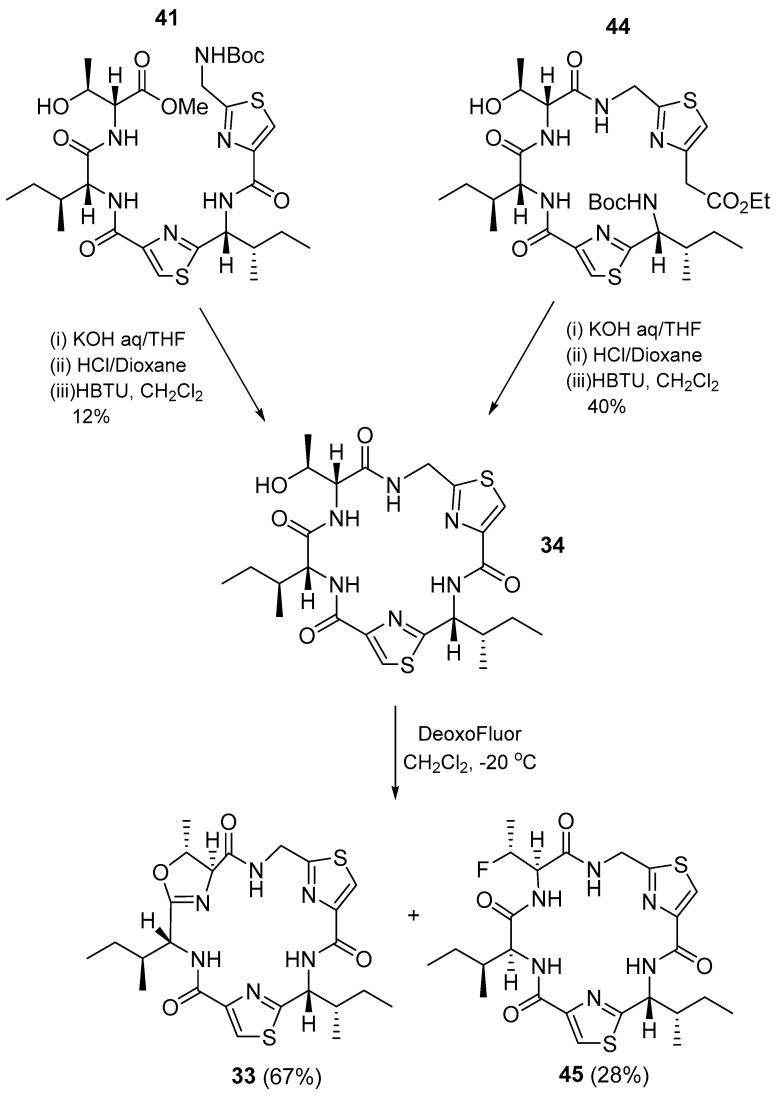
Synthesis of **34** and aerucyclamide B (**33**).

Macrocycle **34** was obtained from intermediates **41** and **44** using HBTU as a coupling agent (Scheme 5), and then aerucyclamide B was synthesized from **37** via intermediate **44**, and from **36** via intermediate **41 [15]**.

#### 3.1.3. Cyclomarin

**Figure 11:**
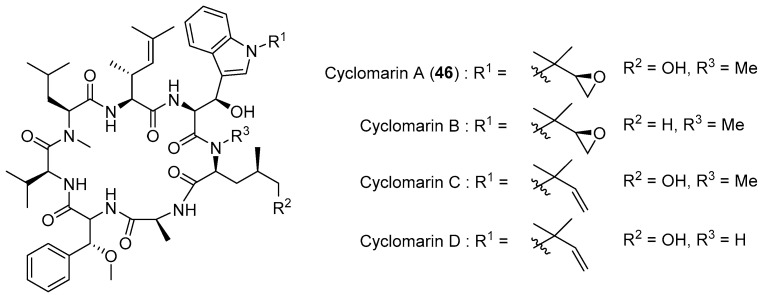
Natural occuring cyclomarins.

Cyclomarin A (**46**) (Figure 11) is a marine cycloheptapeptide with antimalarial activity that can be synthesized in an enantiomerically pure form [16]. Key steps in the synthesis of cyclomarin are asymmetric chelate−enolate Claisen rearrangement, asymmetric hydrogenation, and highly diastereoselective additions of organozinc and titanium reagents [16,17].

**Scheme 6:**
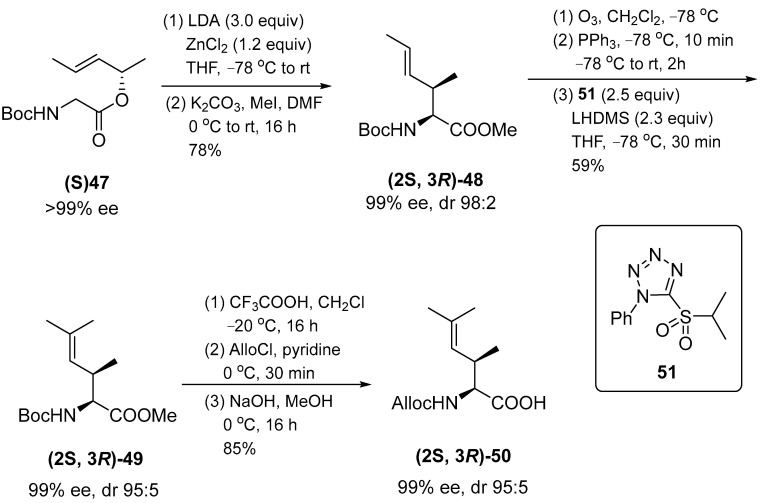
Synthesis of (2*S*,3*R*)–**50**.

The peptide synthesis developed to obtain the required building blocks is based on long-term experience in amino acid synthesis. Amino acid chelate enolate esters are used to obtain β-hydroxy through an aldol reaction, whereas γ,δ-unsaturated amino acids are obtained by transition metal-catalyzed allylic alkylation or Claisen enolate chelate rearrangement and syn-isomers are obtained by Claisen rearrangement. Thus, this approach was used to produce the desired *N*-protected amino acid (2*S*,3*R*)-**50** (Scheme 6).

**Scheme 7:**
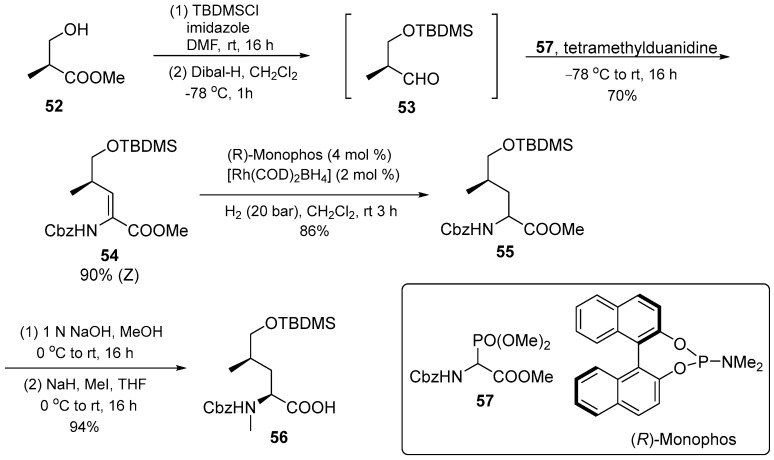
Synthesis of (2*S*,4*R*)–**56**.

The protected 5-hydroxyleucine **56** was synthesized with the commercially available Roche ester **52**. The compound was reduced to the aldehyde **53**, and used in an olefination using Schimdt’s phosphonoglycinate **57**. The α,β-unsaturated amino acid derivative obtained was stereoselectively hydrogenated with (*R*)-monophos as a ligand. Following *N*-methylation and saponification, amino acid **56** was produced with good yield selectivity (Scheme 7).

**Scheme 8:**
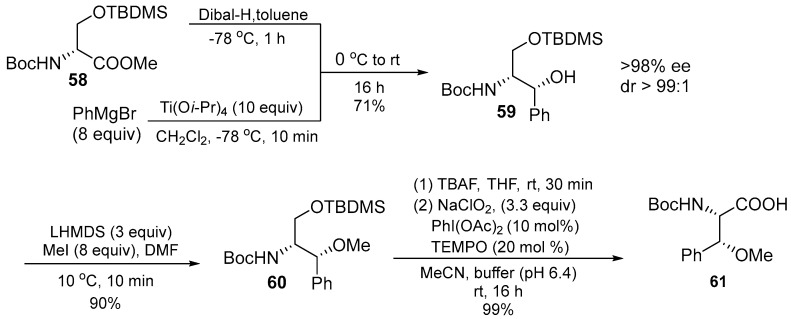
Synthesis of (2*S*,3*R*)–**61**.

β-methoxyphenylalanine **61** is the third unusual building block that was prepared through several attempts shown in Scheme 8. The first attempt using phenylmagnesium bromide (3 equiv.) and DIBAL, which gave an acceptable yield of the addition products (60%), but only moderate diastereoselective products (7:3 ratio). Consequently, the titanium reagent was added as the single diastereomer. Subsequent O-Methylation, cleavage of the silyl ether, and oxidation then gave rise to an excellent yield of the target acid **61**. Another method was also reported for regioselectively prenylated from electron-withdrawing indole. The Pd-catalyzed protocol to 3-indolecarboxylate **62** was applied resulting in indole **63**. The reaction temperature was maintained at 0 °C to suppress *N*-prenylation, the ester was then saponified directly before the peptide coupling to avoid decomposition of the labile acid.

**Scheme 9:**
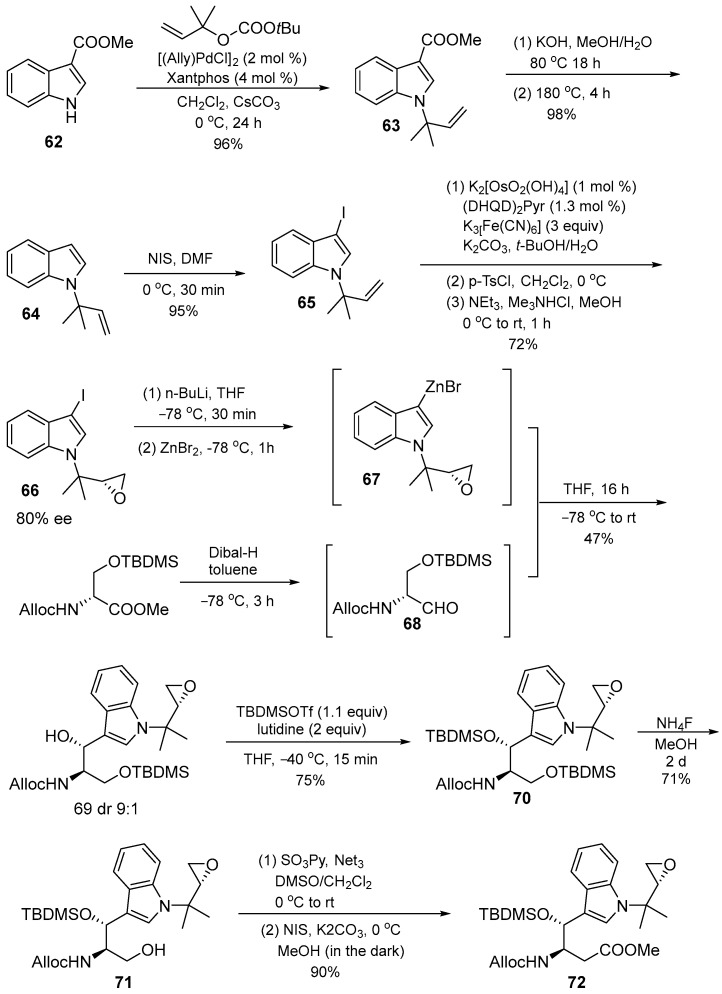
Synthesis of (2*S*,3*R*)–**72**.

The next building block was obtained by the ester saponification of compound **62**. The prenylated indole **64** was converted to the 3-iodo derivative **65** and the terminal double bond was changed to epoxide **66**. After several steps, niodosuccinimide (NIS) in MeOH produced methyl ester **72** (Scheme 9). The O-silylated cyclomarin A was obtained by removing the two protective silyl groups using a two-step protocol in which the primary OH group was deprotected first using NH_4_F. 

**Scheme 10:**
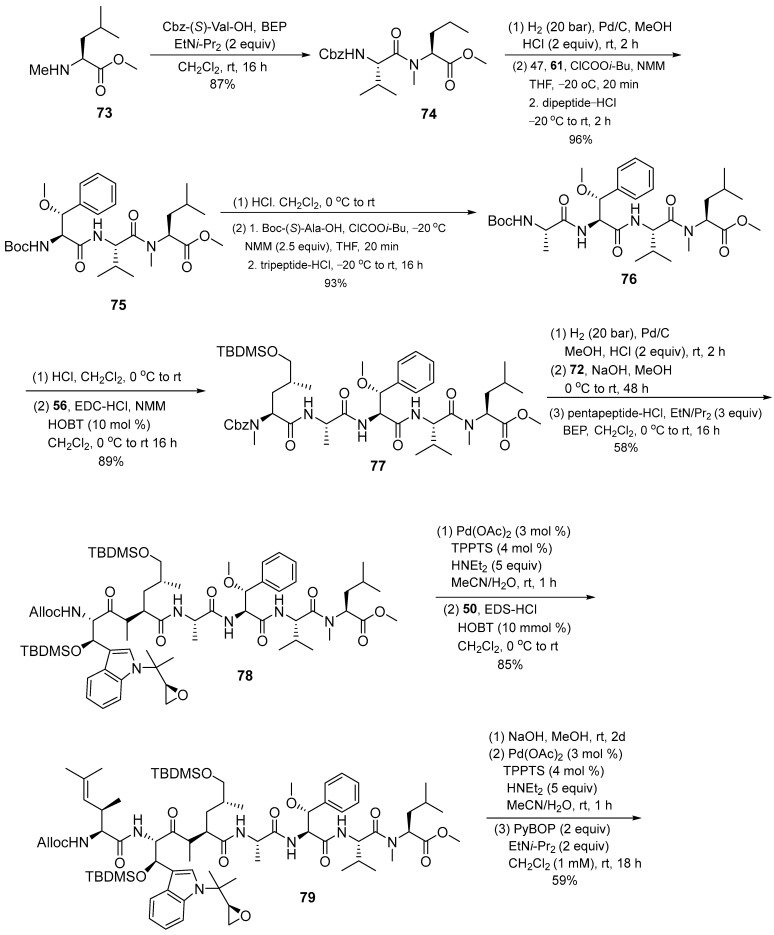

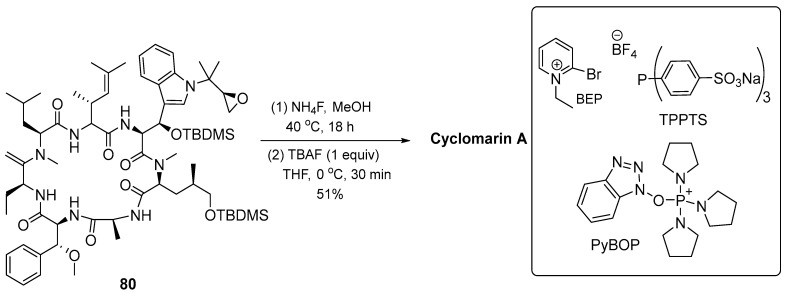
Synthesis of cyclomarin A (**46**).

After obtaining the necessary building blocks, cyclomarin A was then synthesized. Ring closure was planned between aminohexenoic acid and *N*-methylated leucine. BEP (2-bromo-1-ethylpyridinium tetrafluoroborate) **80** was used for coupling with protected valine. HCl was applied to suppress DKP formation during synthesis. The dipeptide salt was coupled to the next peptide with the activated compound **47**. The same protocol was then applied in the next coupling step. The HOBT reagent was used to activate *N*-methyl-4-hydroxyleucine **56** to avoid epimerization during the coupling reaction. This step obtained enantiomerically pure pentapeptide **77**. The methyl ester **72** was saponified and directly coupled using the BEP protocol to obtain compound **78**. Under Pd catalysis, the alloc protecting group was removed and combined with amino acid **50**, then the deprotected heptapetide was slowly added to an aqueous solution of PyBOP and base in CH_2_Cl_2_ to obtain O-sylilated cyclomarin A **46** (Scheme 10) [16].

**Scheme 11:**
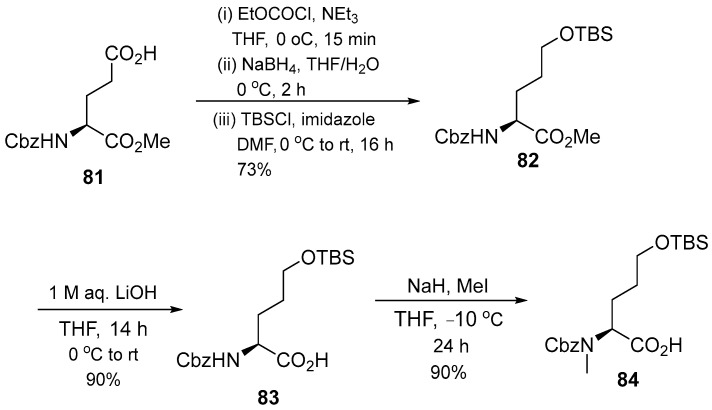
Synthesis of *N*-methylated d-hydroxy norvaline (**82**).

The synthesis of another cyclomarin was also reported by Kiefer et al. (2019). Seventeen desoxycyclomarin-inspired derivatives (**96**–**100**, **106**–**110**, **119**–**125**) were synthesized using a straightforward solution-phase approach [17]. First, an analog library was prepared stepwise by varying the *N*′-side chain of the tryptophan moiety followed by simplifying the aminohexenoic acid unit. A Negishi reaction served as key step for the generation of *N*′-isopropyltryptophan, with final saponification yielding the *N*′-isopropyltryptophan derivative. d-hydroxyleucine units were replaced by d-hydroxynorvaline for additional structural changes in the peptide backbone. The synthesized fragment with protected glutamic acid (**81**) was activated as mixed anhydride and then reduced with NaBH_4_. The alcohol produced as TBS ether gives compound **82**, which is saponified and *N*-methylated to obtain *N*-methyl-d-hydroxynorvaline **84**. The first step of synthesis is focused on linear precursor assembly with varying tryptophan motifs and hexenoic amino acids (Scheme 11).

**Scheme 12:**
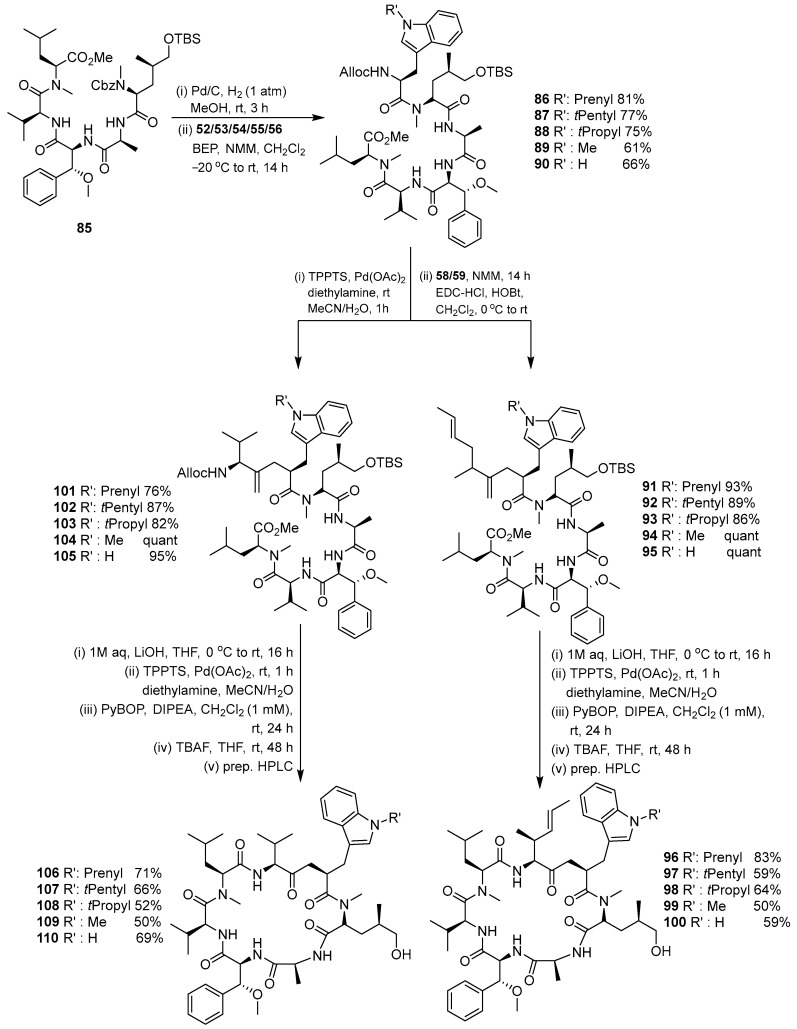
Coupling and macrolactamization of the first cyclomarin analogs.

**Scheme 13:**
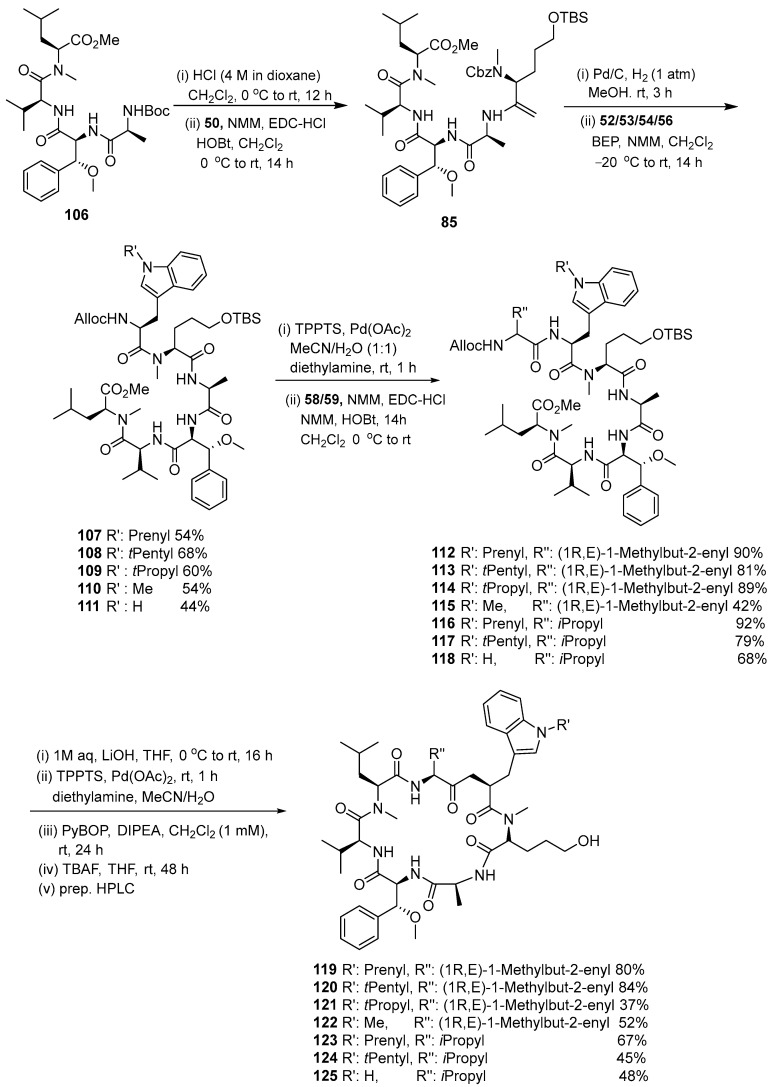
Synthesis of the second set of cyclomarin analogs based on key intermediate **106**.

The intermediate compounds **86**–**90** were reacted with compounds **58**/**59** using the HOBt coupling reagent to produce compounds **91**–**95**, which were then cyclized with PyBOP and DIPEA coupling reagents to produce compound **96**–**100** (Scheme 12). Then, compounds **106**–**110** were produced using the same synthesis steps. Intermediates **101**–**105** were cyclized with the same coupling reagent to produce the target compound. The second set of cyclomarin analogs was synthesized based on the key intermediate **106**. Several stages of coupling and deprotection were conducted to produce linear peptide compounds **112***–***118**. The linear compounds were then cyclized using PyBOP and DIPEA to produce compounds **119**–**125** (Scheme 13). 

#### 3.1.4. Chaiyaphumine

**Scheme 14:**
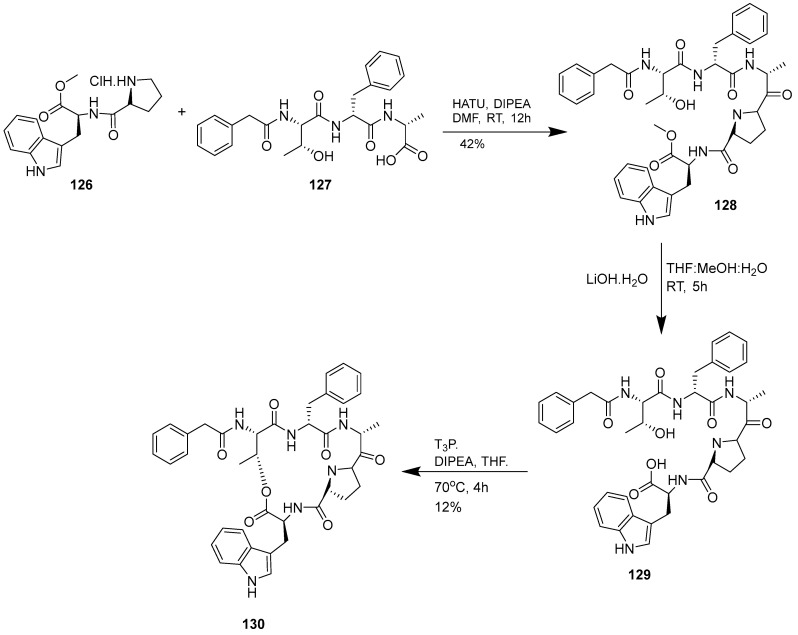
Synthesis of chaiyaphumine A (**130**).

Gholap and Ugale (2017) reported the synthesis of chaiyaphumine-A (**130**) by convergent-based solution phase synthesis involving protection, deprotection, coupling and cyclization reactions [18]. This novel class of peptide will the subject of much research to develop new antimalarial drugs. Five amino acids, L-threonine, D-phenyl alanine, D-alanine, L-proline and L-tryptophan, were assembled by coupling reactions involving HATU. The macrocylization of the side chain (-OH) of L-threonine and C-terminus of carboxylic acid from L-tryptophan using T_3_P yields chaiyaphumine-A (Scheme 14).

**Scheme 15:**
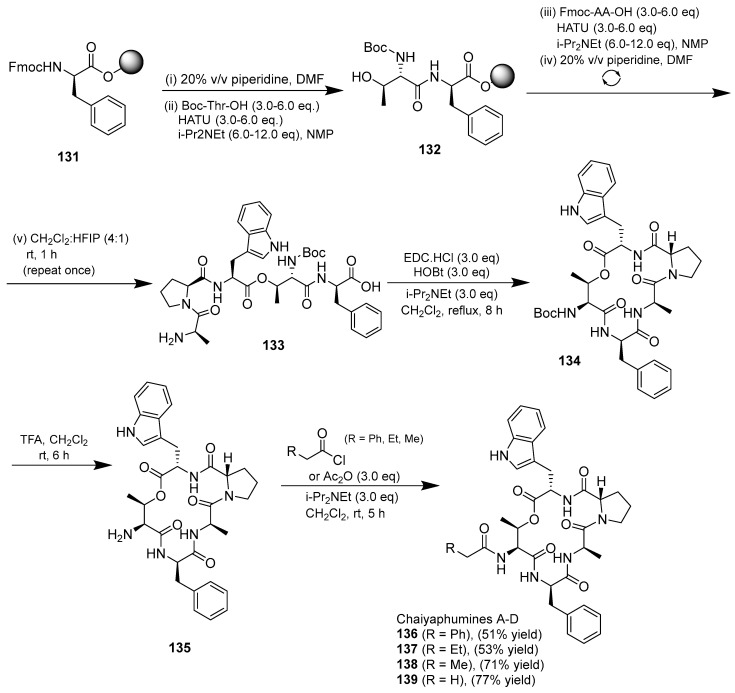
Synthesis of chaiyaphumines A–D.

The synthesis of chaiyapumines was also undertaken by Lu and Batey (2022) (Scheme 15). Fmoc-based solid-phase synthesis approaches were used for the synthesis of the requisite precursors. The solution-phase macrolactamization approach using EDC-HCl/HOBt was achieved without any detectable epimerization. This strategy was successfully applied for the total synthesis of the antimalarial compound chaiyaphumine A, as well as the natural products chaiyaphumine B, C, and D (**136**–**139**) using a late-stage acylation of the threonine amino group [19].

Cyclized products were not produced by the macrolactonization method using MNBA/DMAP/iPr_2_Net, while the use of the additive Dy(OTf)_3_ resulted in epimerization macrocycles. The slow rate of macrolactonization is influenced by the weak nucleophilicity of oxygen and the steric hindrance of threonine in the seco acid precursor, which allows competitive epimerization/cyclization to occur. Successful macrocyclization can be achieved via a macrolactamization approach that allows the total synthesis of all chaiyaphumine compunds.

#### 3.1.5. Cyclopeptides Anolgs Prepared by Macrocyclization

**Scheme 16:**
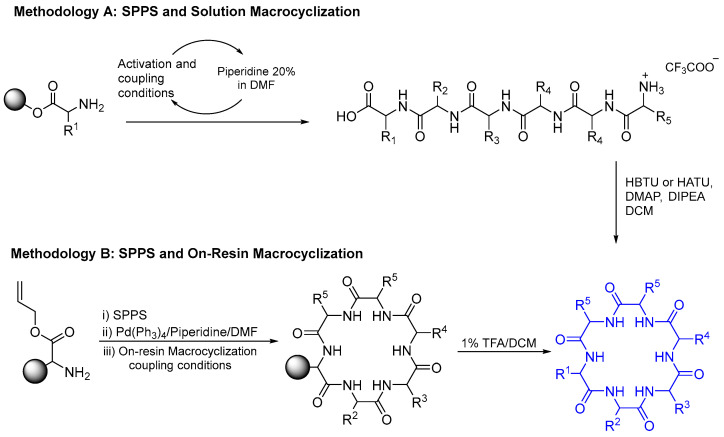
Methods A and B to synthesize cylopeptide.

Fagundez et al. (2018) investigated the synthesis of a cyclopeptide using solid-phase peptide synthesis and on-resin macrocyclization, with various analytical techniques used to monitor reactions and purify compounds [20]. The investigation compared solution- and solid-phase ring-closing reactions following the synthesis of linear precursor on a 2-chrotrytil chloride resin (Scheme 16). Method A applied a combination of the solid-phase (linear precursor) and the solution-phase methods (macrocylization), whereas method B involved all reactions on-resin. A combination of DIC and Cl-HOBt as a coupling agent was used when the next amino acid added was Fmoc-L-Cys(Trt)-OH because it does not require the addition of a base and can decrease potent racemization. The solution-phase macrocyclization involved HBTU or HATU in the presence of DIPEA in DCM. The final products were obtained in moderate to good yields 26–80%.

**Figure 12:**
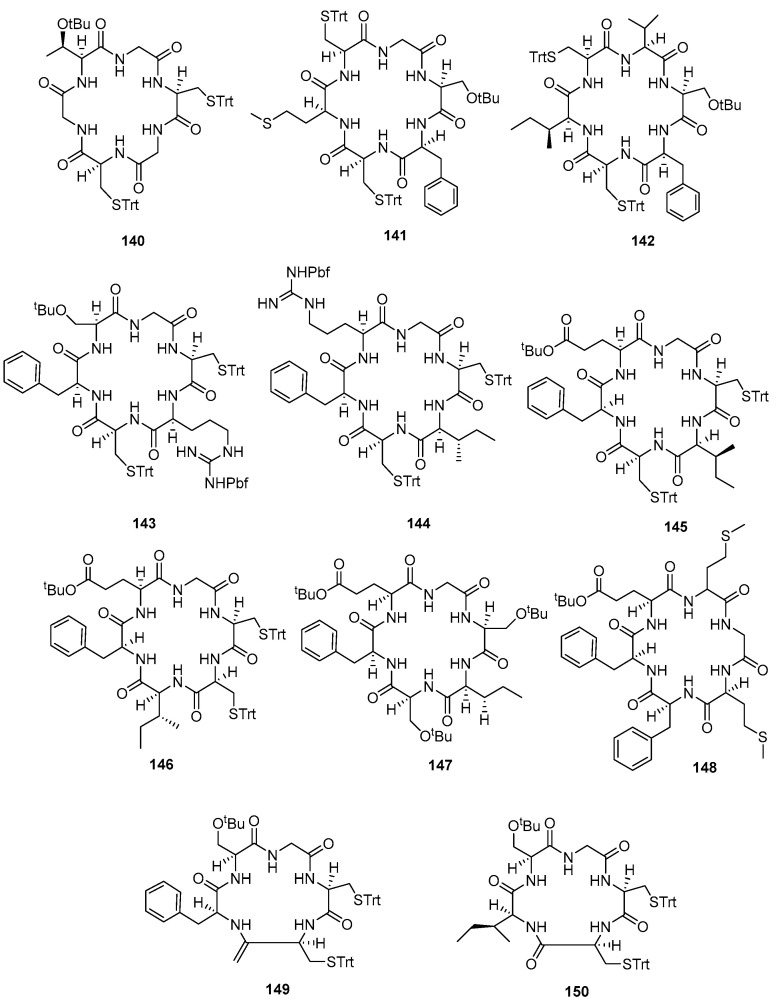
Compounds **140**–**150**.

**Figure 13:**
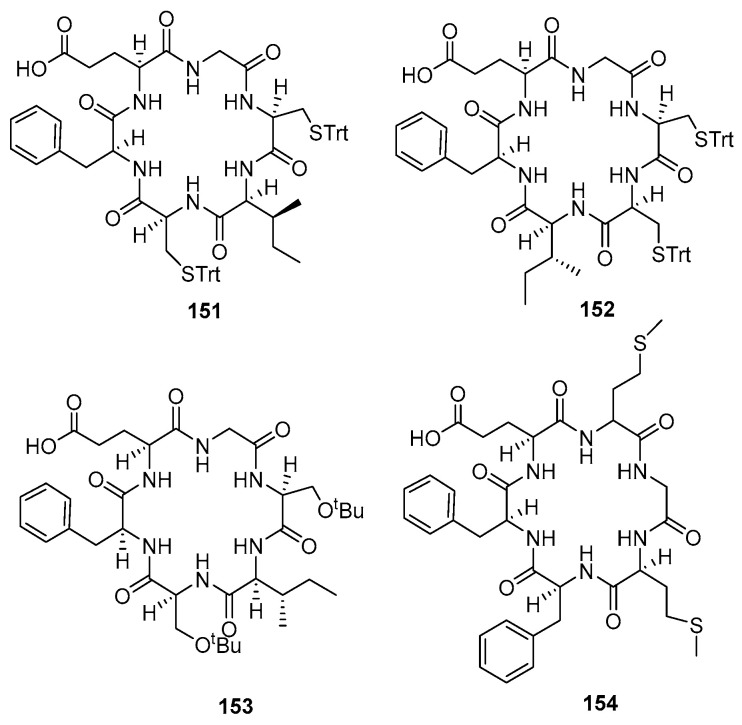
Compounds **151**–**154**.

Eleven cyclic peptides (**140**–**150**) (Figure 12) were synthesized (23 to 63% yield) using method A (Table 2). Method B constructed Fmoc-L-Glu-OH and all amino acids on 2-CTC resin and the capping step took advantage of DIPEA and MeOH (Table 3). Reagent [Pd(PPh_3_)_4_] in a solution of 10% piperidine in THF was used to remove the Fmoc-protected amino acid at the *N*-terminal group and produce a free carboxyl group at the C-terminal. This method yielded four antimalarial cyclopeptides (**151**–**154**) (Figure 13). A comparison of these methods is provided in Table 4.

**Scheme 17:**
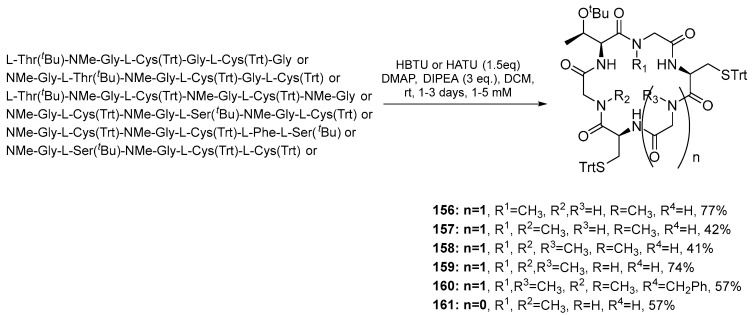
Synthesis of cyclopeptides **156**–**161** by solution macrocyclization.

A year later after synthesizing compounds **140**–**154** cyclopeptides, Fagundez et al. (2019) developed cyclopeptides containing *N*-methyl amino acids with promising antiplasmodial activity against both erythrocytic and liver stages of malaria in vitro. These compounds could potentially be used as new and safe drugs to combat malaria [21]. Compounds **156**–**161** were synthesized by employing solid-phase linear peptide synthesis and solution macrocyclization (Scheme 17). The 2-chlorotrityl resin (2-CTC) was used to decrease the diketopiperazine formation, and HBTU and DIPEA were employed as coupling reagents in most cases. HCTU and DIPEA reagents were used to combine *N*-Me-Gly with the next amino acids because they are more effective. DIC and Cl-HOBt were then used as reagents to activate the Fmoc-L-Cys(Trt)-OH and coupling to resin-attached peptides to minimize racemization of Cys residues. 

**Scheme 18:**
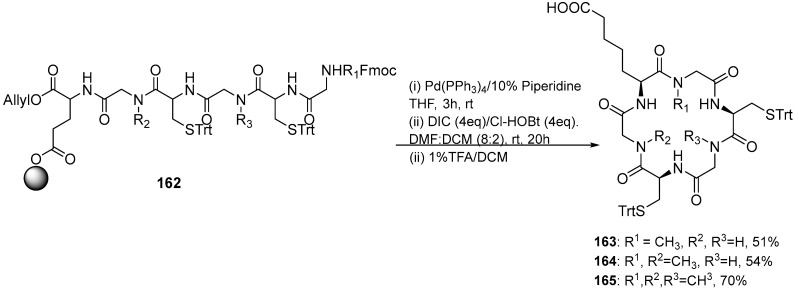
Synthesis of cyclopeptides **163**–**165** by on-resin cyclization.

**Figure 14:**
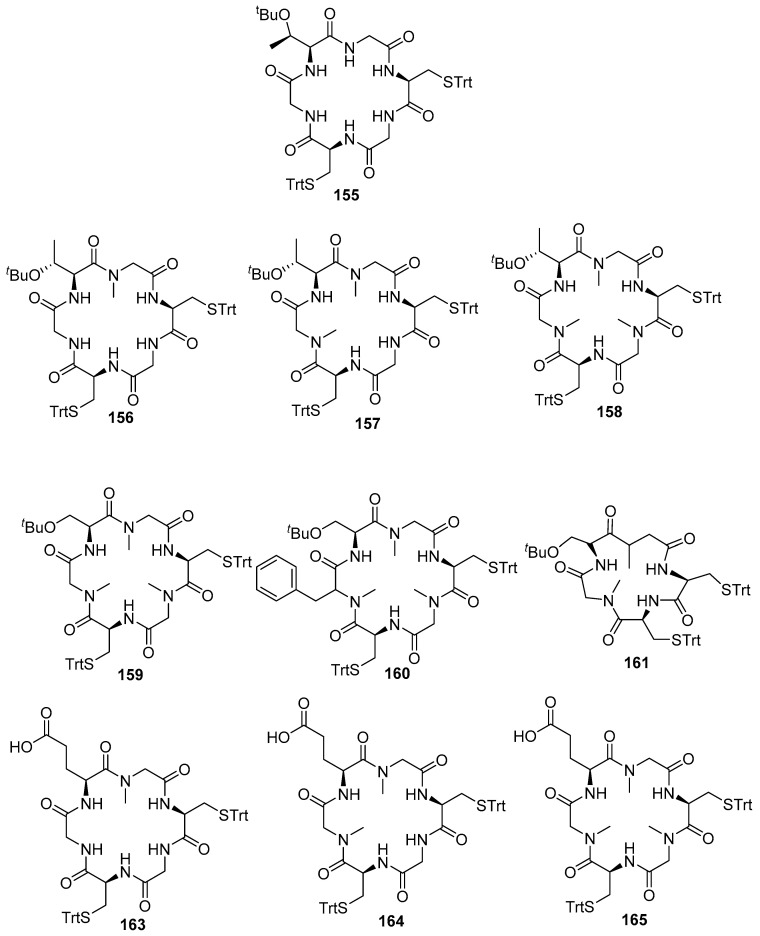
Structure of cyclopeptides analogs (**156**–**165**) and lead compound **155**.

Compounds **162**, **163**, and **164** were synthesized employing on-resin macrocyclization (Scheme 18). First, Fmoc-L-Glu-O was anchored to 2-CTC resin with DIPEA, and then the Fmoc SPPS protocol was followed. After the removal of the allyl ester and Fmoc group with [Pd(PPh_3_)_4_] in a solution of 10% piperidine in THF, the ring closure was performed using DIC and Cl-HOBt. Figure 14 shows the structure of compounds **156**–**165**.

#### 3.1.6. Hirsutellide A and Its Analogues

**Figure 15:**
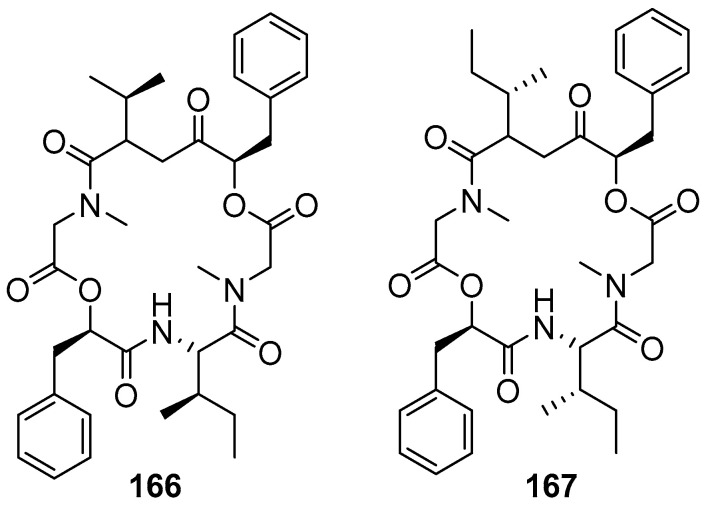
Structure of hirsutellide A (**166**) and its synthetic stereoisomer (**167**).

Sahile et al. (2020) reported the synthesis and evaluation of hirsutellide A (Figure 15) and its analogs for their antimycobacterial and antiplasmodial activities [22]. Optimizing the structures of lipophilic antimicrobial cyclic peptides resulted in more membrane-permeable bioactive peptides, such as the lipophilic antimicrobial depsipeptide (hirsutellide A). The first report of the synthesis hirsutellide A was by Xu et al. in 2005; compound **167** had Ile residues instead of allo-Ile resulting in stereoisomers that do not have antimycobacterial potential [22,23].

**Scheme 19:**
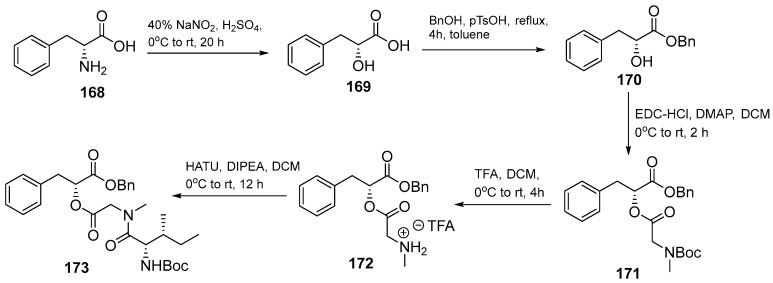
Synthesis of the protected tridepsipeptide, a precursor of hirsutellide A (**173**).

The compound was synthesized by a solution-phase method and initiated by the preparation of protected tridepsipeptide (Scheme 19). D-Phe **168** was hydrolyzed into α-hydroxyl carboxyl acid **169**. After several steps, dipepsipeptide **172** coupling with N-Boc-allo-Ile-OH using HATU/DIPEA to generate tridepsipeptide **173**.

**Scheme 20:**
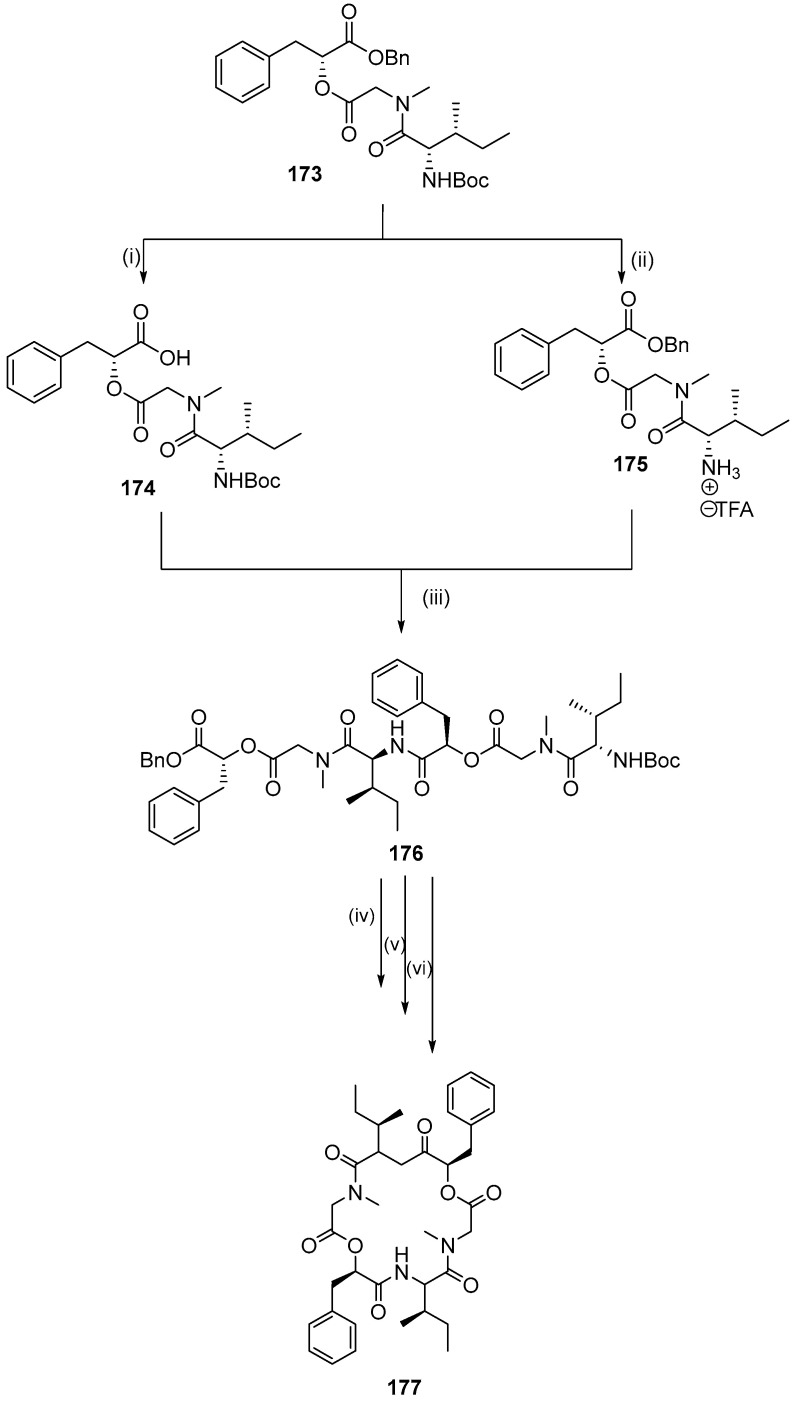
Synthesis of hexadepsipeptide precursor of hirsutellide A and macrolactamizations. Reagents and conditions: (i) 10% Pd/C, H_2_ (5 atm), EtOAc, rt, 12 h; (ii) TFA, DCM, 0 °C to rt, 4 h; (iii) BOP-Cl, DIPEA, 0 °C to rt, 18 h; (iv) 10% Pd/C, H_2_ (5 atm), EtOAc, rt, 12 h; (v) TFA, DCM, 0 °C to rt, 5 h; (vi) HATU, HOBt, DMF, 0 °C to rt, 3 days.

The desired cyclic depsipeptide **177** (Table 5) was obtained via macrocyclization of the precursor **176** using HATU/HOBt/DIPEA in DMF (Scheme 20). Furthermore, depsipeptide (**178**–**181**) analogs and peptide analogs (**182**–**188**) of hirsutellide A were designed and synthesized for preliminary SAR studies with a combination of solution and solid phase method (Table 5). 

#### 3.1.7. Macrocyclization Strategies

Recently, Zhang et al. (2022) synthesized macrocyclic peptides as species-selective antimalarial proteasome inhibitors [24]. The compounds were prepared through several macrocyclization strategies such as Suzuki coupling, intermolecular amidation, intermolecular alkylation, intramolecular alkylation, and ring-closing metathesis (RCM) reaction. 

**Scheme 21:**
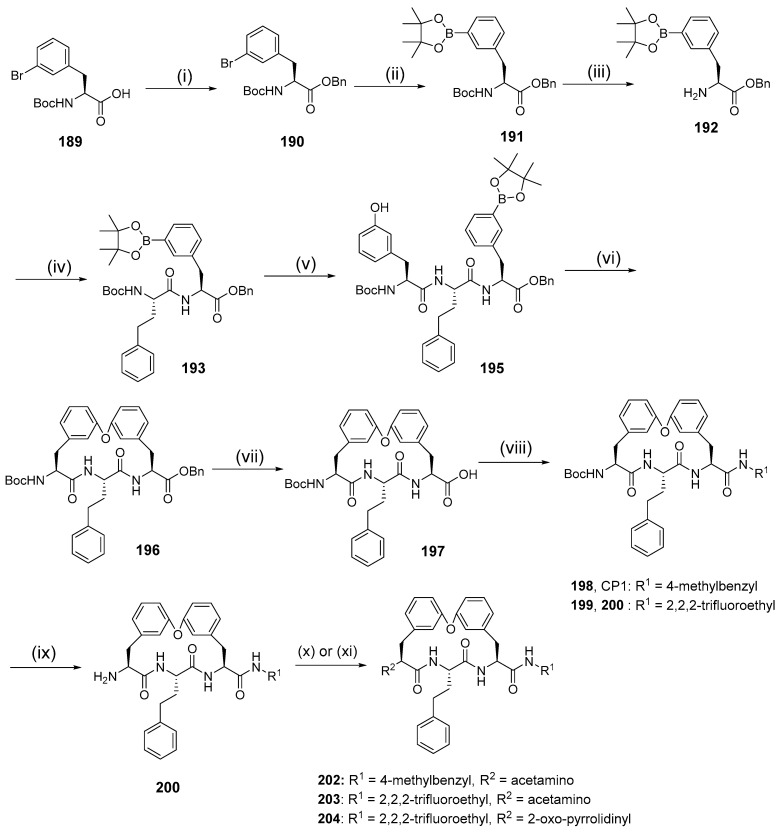
Synthesis of cyclic peptides **202**, **203**, **204** using the Suzuki coupling reaction as a macrocylization strategy. Reagents and conditions: (i) BnBr, K_2_CO_3_, acetone, r.t; (ii) Bis(pinacolato)diboron, KOAc, Pd(dppf)Cl_2_, dioxane, 80 °C; (iii) 4 N HCl/dioxane; (iv) Boc-L-homophenylalanine, HOBt, EDCI, DCM; (v) (1) 4 N HCl/dioxane, (2) carboxylic acid, HOBt, EDCIM DCM; (vi) (1) NaIO_4_, KOAc, acetone, H_2_O, (2) Cu(OAc)_2_, TEA, 4 Å molecular sieves, MeOH, DCM, r.t. 12 h; (vii) Pd/C, H_2_; (viii) R^1^NH_2_, HOBt, EDCI, DMF; (ix) TFA, DCM; (x) acetal chloride, TEA, DMF; (xi) (1) tert-butyl-4-bromobutanoate, K_2_CO_3_, DMF, 50 °C, (2) TFA, DCM, (3) EDCI, HOBt.

Synthesis of cyclic peptides **202**, **203**, and **204** took advantage of Suzuki coupling as a macrocyclization strategy (Scheme 21). Benzylation and borylation reactions of N-Boc-3-bromophenylalanine **189** produced boronate. Deprotection of Boc in compound **191** was followed by combining with Boc-L-homophenylalanine to produce dipeptide intermediate **193**, which then underwent Boc deprotection and condensation with carboxylic acid **194** to produce compound **195**. Oxidative hydrolysis of boronic ester to boronic acid in **195** by NaIO_4_ was followed by an intramolecular Chan–Lam coupling reaction to produce macrocycle **205**. Removal of the benzyl protective group by catalytic hydrogenation, followed by a coupling reaction of urea, produces **198**–**199**, which undergoes Boc deprotection to yield amines **209** and CP1. Furthermore, compounds **200** and CP1 undergo acetylation of free amines to produce macrocycles **202** and **203**. Macrocycle **204** is produced from amine **210** through subsequent alkylation with 4-bromobutanoate, Boc-deprotection, and lactamization using EDCI and HOBt.

**Scheme 22:**
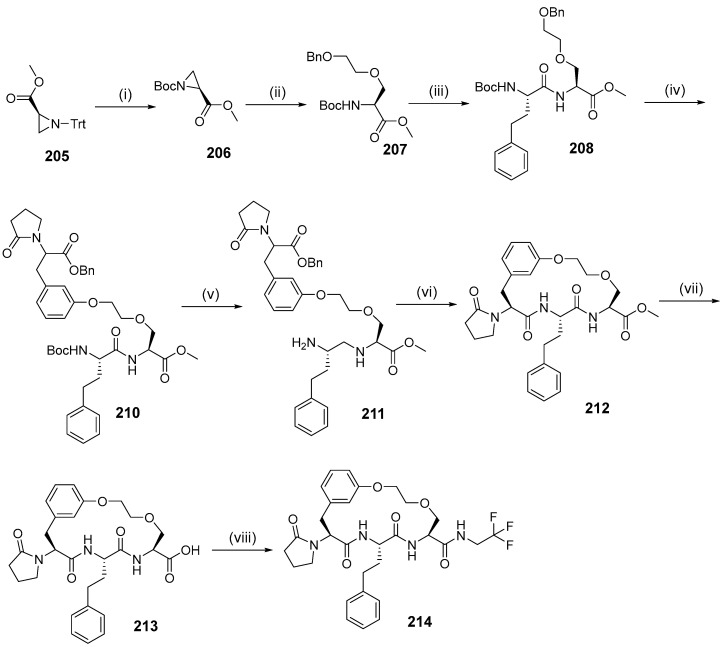
Synthesis of compound **214** using intramolecular amidation as the macrocyclization strategy. Reagen and conditions: (i) (1) TFA, DCM, MeOH, (2) Boc_2_O, TEA, MeCN; (ii) BF_3_.Et_2_O, DCM, 2-(benzyloxy)ethanol; (iii) (1) HCl, MeOH, (2) HBTU, DIOEA, DMF, Boc-L-homophenylalanine; (iv) (1) H_2_, Pd/C, MeOH, (2) DEAD, PPh_3_, THF, compound 16; (v) (1) Pd/C, H_2_, MeOH, (2) HCl/dioxane; (vi) EDCI, HOBt, DMF; (vii) NaOH, THF/H_2_O; (viii) 2,2,2-Trifluoroethylamine, EDCI, HOBt, pyridine.

Intramolecular amidation as a macrocyclization strategy was used to synthesize compound **214** (Scheme 22) from compound **212** by hydrolysis of methyl ester and a coupling reaction using trifluoroethylamine. The synthesis began with deprotecting the tritile group in aziridine **205**, then protecting it again with the Boc group present in Boc-aziridine **206**. Compound **207** was then produced by an SN_2-_type ring opening reaction mediated by BF_3_·Et_2_O on aziridine **206** with 2-benzyloxyethanol. Boc-deprotection in compound **207** was followed by a coupling reaction with Boc-L-homophenylalanine to produce dipeptide **208**, then benzyl deprotection and Mitsunobu reaction of fragment **209** yielded the macrocyclization precursor **210**. Debenzylation and Boc deprotection of compound **210** was followed by intramolecular mediation to produce macrocycle **212**.

**Scheme 23:**
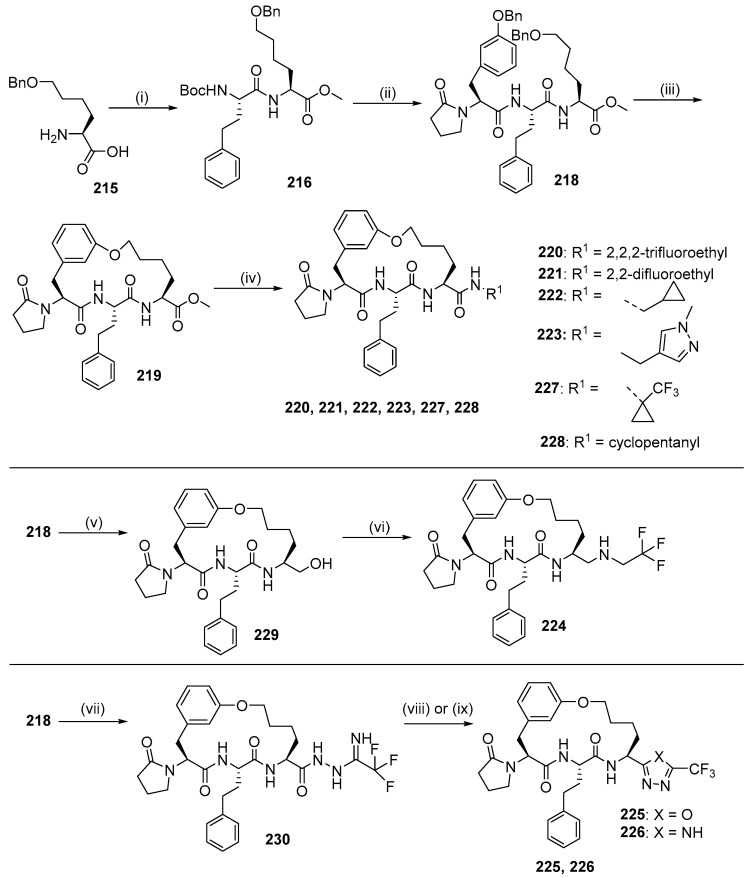
Synthesis of compounds **220**–**226** using intramolecular alkylation as a macrocyclization strategy. Reagents and conditions: (i) (1) HCl, MeOH, (2) Boc-L-homophenylalanine, HATU, DIEA, DMF; (ii) (1) HCl (4 M), dioxane, (2) compound **23**, HATU, DIEA, DMF; (iii) (1) H_2_, Pd/C, Pd(OH)_2_, MeOH, (2) TosCl, pyridine, (3) Cs_2_CO_3_, DMF; (iv) (1) NaOH, THF, H_2_O, (2) HATU, DIEA, R^1^NH_2_; (v) LiBH4, THF; (vi) (1) Dess-Martin, THF, (2) 4 Å MS, NaBH(OAc)_3_, MeOH, 2,2,2-trifluoroethylamine; (vii) (1) N_2_H_4_.H_2_O, MeOH, (2) methyl trifluoroacetamide, DCM; (viii) 1,2-dichlorobenzene; (ix) (1) TFA, DCM, (2) Burgess reagent, THF.

The synthesis of compounds **229**–**237** were involved in intramolecular alkylation as a macrocyclization strategy (Scheme 23). Dipeptide **225** was prepared from amino acid **224** by methyl esterification and HATU-mediated coupling reaction with Boc-L-homophenylalanine. Boc deprotection of dipeptide **225** and a coupling reaction with fragment **226** gave tripeptide **227**, which was then removed by two benzyl groups. The primary alcohol was chemoselectively protected with Tos groups, and Cs_2_CO_3_-mediated intramolecular. It underwent alkylation to give macrocycle **228**. After treatment of the methyl ester with sodium hydroxide, coupling of the free acid with various primary amines yielded macrocycles **229**, **230**, **231**, **232**, **236**, and **237**. Reduction of the methyl ester **228** with lithium borohydride formed alcohol **238**, and then Dess–Martin oxidation, followed by reductive amination with trifluoroethylamine, yielded macrocycle **233**. Condensation of the methyl ester **228** with hydrazine, followed by coupling with methyl trifluoroacetimidate gave the hydrazide **239**, which was thermally cyclized to give the 1,2,4-triazole **235**. Subsequent hydrolysis and dehydration of hydrazide **239** in the presence of the Burgess reagent gave 1,3,4-oxadiazole **234**.

**Scheme 24:**
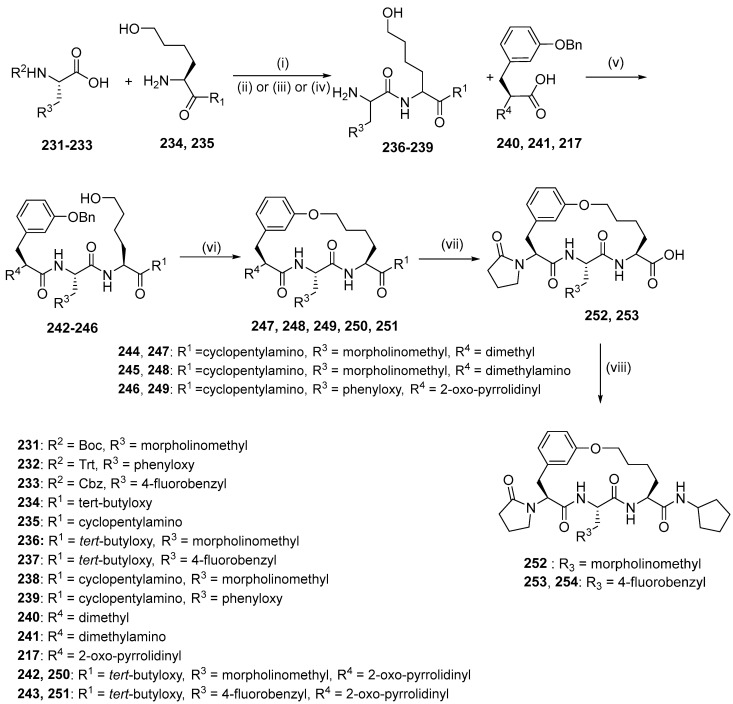
Synthesis of compounds **256**, **257**, **258**, and **263** using intramolecular alkylation as macrocyclization strategy. Reagents and conditions: (i) T_3_P, DIEA, DMF 0–20 °C, 1 h; (ii) HCl (4 M, 4 eq.), dioxane, MeOH; (iii) HCl, dioxane, DCM, 20 °C, 3 h; (iv) H_2_, Pd/C, TFA, THF; (v) T_3_P, DIEA, DMF; (vi) (1) TosCl, pyridine, (2) H_2_, Pd(OH)_2_/C, MeOH, (3) Cs_2_CO_3_, DMF; (vii) TFA, DCM; (viii) cyclopentylamine, T_3_P, DIEA, DMF 0 °C, 1 h.

Macrocyclization with the intramolecular alkylation strategy was used to synthesize compounds **247**, **248**, **249**, and **254** (Scheme 24). Fragments **231**–**233** underwent an amide coupling reaction with fragments **234**–**235** and subsequent deprotection, yielding dipeptides **236**–**239**, which were coupled to fragments **240**–**241** and **217** to yield tripeptides **242**–**246**. The tripeptides **242**–**246** were sulfated with a hydroxyl group, debenzylation of aryl benzyl ether, and intramolecularly substituted between the phenol group and the sulfonate ester, giving macrocycles **250**, **251**, **247**, **248** and **249**. Removal of the tert-butyl from compounds **250**–**251** using TFA was followed by an amide coupling reaction with a primary amine, which produced macrocycles **254** and TDI8.

**Scheme 25:**
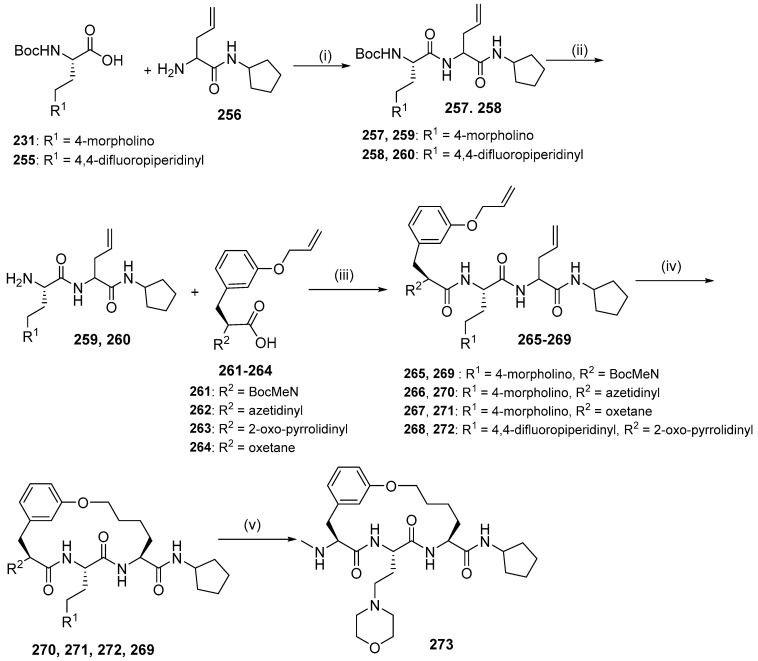
Synthesis of compounds **270**, **271**, **272**, and **273** using RCM reaction as a macrocyclization strategy. Reagent and conditions: (i) T_3_P, DMF, DIEA; (ii) HCl/dioxane, MeOH; (iii) T_3_P, DIEA, DMF; (iv) (1) Grubbs 2^nd^, DCE, (2) H_2_, Pd/C, MeOH; (v) HCl, dioxane.

Synthesis of compounds **270**, **271**, **272**, and **273** was achieved by the RCM reaction (Scheme 25). Fragments **231** and **255** underwent subsequent amide coupling reaction with fragment **256**; Boc-deprotection and an amide coupling reaction with acids **261**–**264** afforded diolefins **265**–**268**, which were subjected to RCM in the presence of Grubbs Catalyst second Generation and reducing of the C=C double bond, yielding macrocycles **270**, **271**, **272**, and **269**. Removal of the Boc protection of **269** provided macrocycle **273**.

### 3.2. Linear Peptides

#### 3.2.1. Falcitidin

**Scheme 26:**
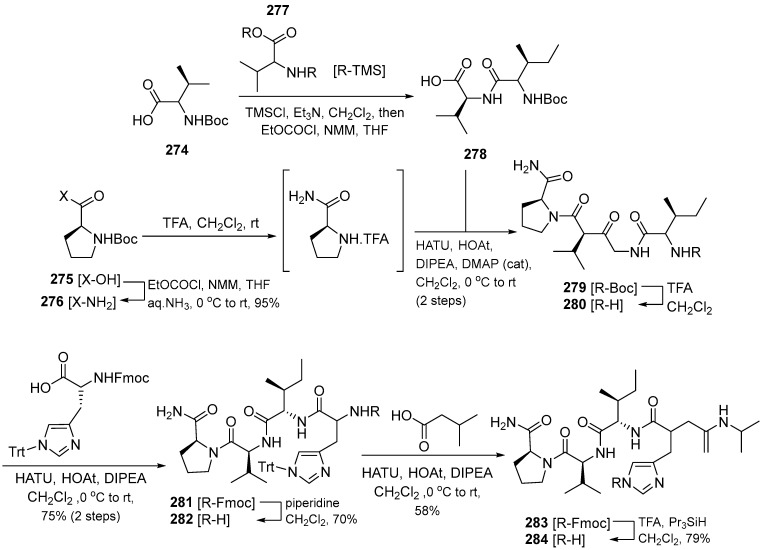
Synthesis of compound **284**.

Somanadhan et al. (2013) succeeded in synthesizing linear antimalarial peptides. Falcitidin **284** is the first member of a new class of falcipain-2 inhibitors, which is a cysteine protease used by *P. falciparum* to degrade hemoglobin during the trophozoite stage of infection [25]. Falcitidin contains isovaleric acid-D-His-L-Ile-L-Val-L-Pro-NH_2_ and was synthesized by the solution-phase peptide synthesis method (Scheme 26). The synthesis begins with deprotecting the Boc group from N-Boc-proline carboxamide using TFA. The amino-proline TFA salt was coupled with freshly prepared L-Ile-L-Val using HATU/HOAt to give L-Ile-L-Val-L-Pro-NH_2_. The dipeptide was obtained by coupling L-Ile-NHBoc with the in situ prepared bis-trimethylsilane (TMS) ether of L-Val under mixed anhydride conditions. The Boc group of the tripeptide was then cleaved using TFA and coupled to Na-Fmoc-N(im)-trityl-D-histidine using HATU/HOAt to give the Fmoc–tetrapeptide. The Fmoc group of was removed using piperidine and HATU/HOAt. The final synthetic target falcitidin was obtained after trityl deprotection of Trt-falcitidin using TFA in the presence of triisopropylsilane.

**Scheme 27:**
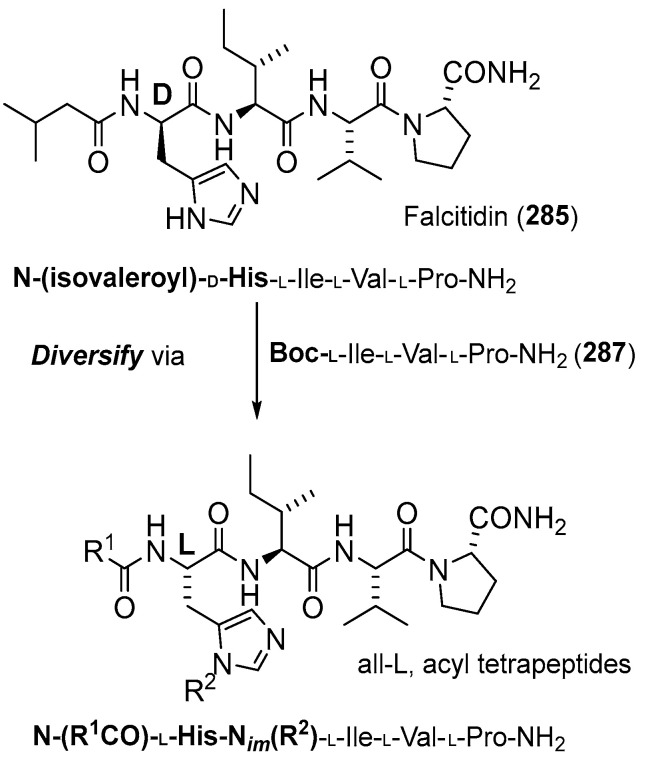
Falcitadin-derived **285** acylpeptide lead compound template.

**Scheme 28:**
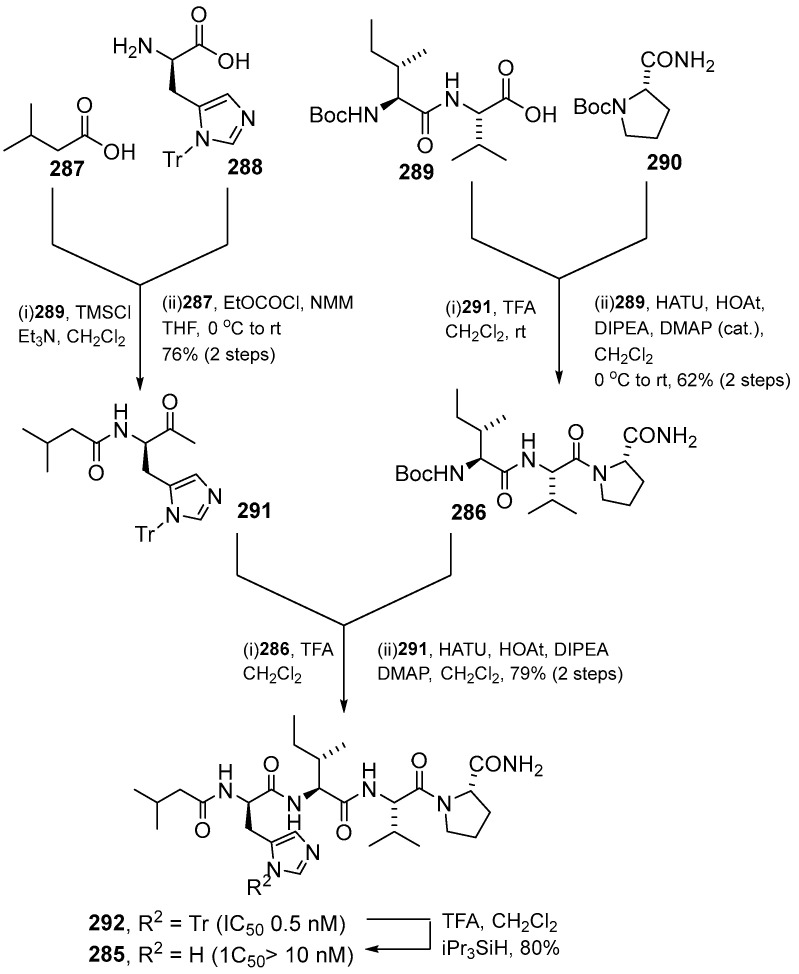
Convergent total synthesis of falcitidin **285** via the key tripeptide intermediate **286** and the whole-cell active N-trityl analog **292**.

**Scheme 29:**
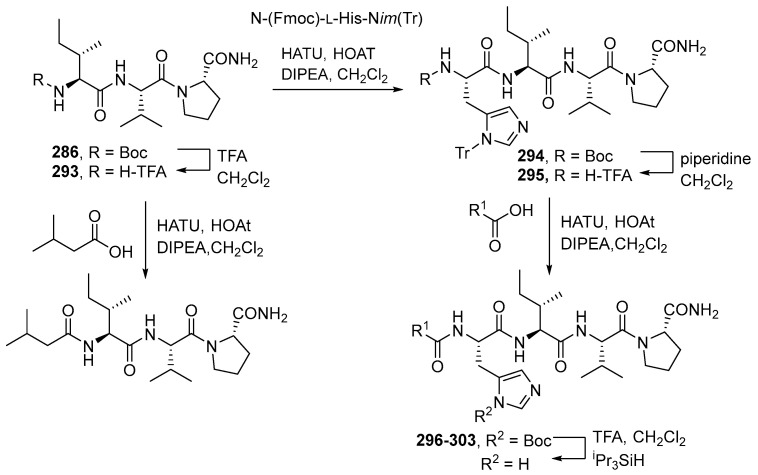
Diverted synthesis of all-L, N-acyl tetrapeptide analogs of falcitidin **285** via the common tripeptide N-Boc-L-Ile-L-Val-L-Pro-NH_2._

Kotturi et al. (2014) synthesized falcitidin and its analogs (**296**–**303**) using the same solution phase peptide synthesis (Scheme 27) [26]. First, N_im_-trityl-D-histidine **288** was converted into bis-TMS ether with TMSCl/Et_3_N_7_ and coupled immediately with the mixed anhydride of isovaleric acid **287** using ethyl chloroformate/NMM to form the trityl protected N-acyl-D-histidine. In parallel, the N-Boc proline amide was deprotected with TFA, and the crude amine salt was coupled immediately with the known dipeptide N-Boc-L-Ile-L-Val by using HATU/HOAt to yield the key tripeptide intermediate N-Boc-L-Ile-L-Val-L-Pro-NH_2_. Lastly, the Boc group of tripeptide was cleaved using TFA and coupled to N-acyl-D-histidine using HATU/HOAt to give the N_im_-trityl protected tetrapeptide **292** (Scheme 28). Falcitidin acylatide and its analogs produce eight analogs compounds (**296**–**303**) by diversifying the synthesis of the N-acyl tetrapeptide analog of falcitidin through the common tripeptide N-Boc-L-Ile-L-Val-L-Pro-NH_2_ (Scheme 29) (Table 6).

#### 3.2.2. Gallinamide

**Scheme 30:**
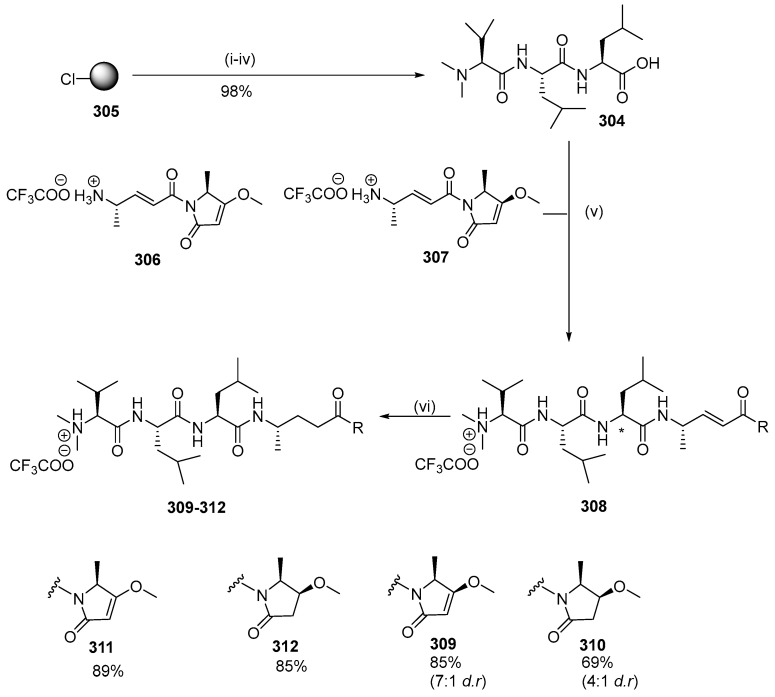
Synthesis of gallinamide A analogs **309**–**312**. Reagents and conditions: (i) resin loading: Fmoc-Leu-OH, iPr_2_EtN, DMF/CH_2_Cl_2_; (ii) Fmoc-SPPS (deprotection, 10 vol % piperidine*/*DMF; coupling, 4 equiv Fmoc-AA-OH, 4 equiv PyBOP, 8 equiv NMM, DMF; capping, 10 vol % Ac_2_O/pyridine); (iii) HCHO, NaBH(OAc)_3_, AcOH, DMF; (iv) 30 vol % HFIP/CH_2_Cl_2_; (v) HATU, NMM, 0 °C for 20 min, rt for 2 h; (vi) H_2_, Pd/C, MeOH. * indicates the site of diastereoisomerism in **309** and **310**.

Gallinamide A is a linear peptide that has antimalarial activity. Conroy et al. (2014) designed and synthesized 18 gallinamide A analogs by solid-phase synthesis and substitution of amino acid residues [27]. Four gallinamide A analogs (**309**–**312**) were originally designed with varying levels of saturation, including compound **309**, which is structurally identical to gallinamide A. Another analog was compound **310** with a reduced methoxy-enol moiety in the pyrolinone ring **309**. Part of the olefinic component of the 4(*S*)-amino-2-(*E*)-pentenoic acid unit is reduced in compound **311** and **312** has reduced olefin groups. Analog synthesis was initiated by synthesizing the *N*-terminal fragment **304** by solid-phase peptide synthesis using the Fmoc strategy. The 2-CTC resin is filled with Fmoc-Leu-OH followed by the subsequent incorporation of the amino acid. Reductive amination of the resin, followed by cleavage of the resin using hexafluoroisopropanol (HFIP), produced an excellent yield of tripeptide **304** which was coupled with imide fragments **306** and **307** prepared using a similar protocol. The coupling reaction involved HATU at low temperature to avoid epimerization to produce good yields of analogs **309** and **310**. At this stage, **309** and **310** underwent hydrogenation to give **311** and **312** (Scheme 30).

**Scheme 31:**
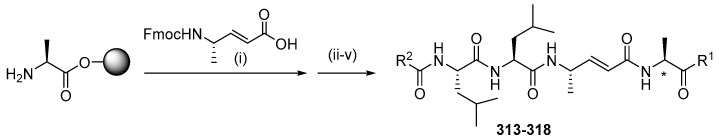
Synthesis of gallinamide A analogs **322**–**327**. Reagents and conditions: (i) 4 equiv amino acid, 4 equiv PyBOP, 6 equiv NMM; (ii) Fmoc SPPS (deprotection, 10 vol % piperidine/DMF; coupling, 4 equiv Fmoc-AA-OH, 4 equiv PyBOP, 8 equiv NMM, DMF; capping, 10 vol % Ac_2_O/pyridine); (iii) HCHO, NaBH(OAc)_3_, AcOH, DMF; (iv) 30 vol % HFIP/DCM; (v) # R^1^-NH_2_, PyBOP, DMF, 0 °C. * indicates the site of diastereoisomerism in **313**–**318**.

Analogs **313**–**318** were synthesized from 2-CTC resin filled with Fmoc-Ala (Scheme 31; Table 7). The coupling of Fmoc-protected α,β-unsaturated amino acids was followed by elongation through the Fmoc solid-phase peptide synthesis strategy. With reductive methylation of the N-terminus using formaldehyde and sodium cyanoborohydride, the peptide was cleaved from the resin using HFIP to produce the C-terminal peptide acid. C-terminal functionality was achieved through benzylamine coupling using PyBOP at low temperatures, and compounds **313**, **315**, **317**, and **318** were purified by RP-HPLC and demonstrated no significant epimerization. Compound **314** was produced by attaching 4-(*R*)-hydroxy L-proline methyl ester to the C-terminus of the two peptides with the addition of NMM as a hindered base. Aminothiazole was coupled to the C-terminus of one peptide acid using PyBOP at low temperature to yield compound **316** as a mixed diastereoisomer.

**Scheme 32:**
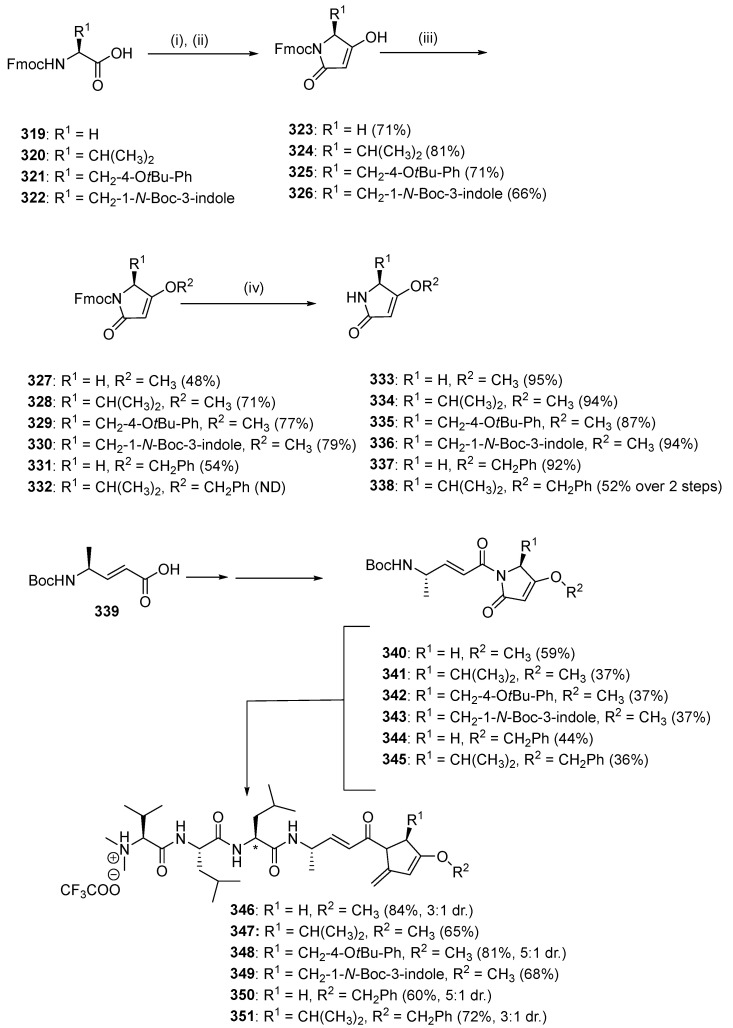
Synthesis of second-generation gallinamide A analogs **346**–**351**. Reagents and conditions: (i) (1) Meldrum’s acid, EDC, DMAP, CH_2_Cl_2_, 0 °C to rt; (ii) EtOAc, 77 °C; (iii) R^2^-OH, DIAD, PPh_3_, CH_2_Cl_2_, 0 °C to rt; (iv) 1:4 *v*/*v* piperidine/MeCN, rt, 15 min; (v) CF_3_COOC_6_F_5_, pyridine, DMF, 0 °C; (vi) **333**–**337**, n-BuLi, THF, −78 °C; (vii) 1:1 *v*/*v* TFA/CH_2_Cl_2_; (viii) tripeptide **333**, HATU, NMM, DMF, 0 °C. ND = not determined due to co-contamination with DIAD by-product. * indicates the site of diastereoisomerism in **346**, **348**, **350**, and **351**.

A further six analogs (**346**–**351**) were designed, possessing the identical peptide backbones to **309** and **310** but with a variation in the side chain on the pyrolinone unit and the enol substitution of pyrolinone (Scheme 32). The synthesis of compounds **346**–**351** was initiated by preparation of the requisite pyrolinones **333**–**338** from Fmoc-protected amino acids **319**–**322**. Then, Meldrum’s acid in ethyl acetate was coupled with amino acids **319**–**322** using EDC and DMAP followed by reflux of the Meldrum’s adduct in ethyl acetate. This step affected the condensate cyclization to give Fmoc-protected pyrolinones **323**–**326**, which were then reacted with methanol under Mitsunobu conditions using DIAD and triphenylphosphine, respectively, to produce O-methylated pyrolinones **327**–**330**. Compounds **323**–**324** were reacted with benzyl alcohol under the same conditions to produce **331**–**332**. Compounds **333**–**338** were produced by treating piperidine in acetonitrile. After each pyrrolinone building block was obtained, amino acid **339** was activated as the corresponding pentafluorophenyl ester. Separately, pyrolinones **333**–**338** were deprotonated with n-butyllithium at low temperatures before the addition of pentafluorophenyl ester to yield **340**–**345** (36–59%). Due to the unfavorable results, acidolysis of the Boc groups from **340**–**345** was followed by coupling to the N-terminal tripeptide **304** using the coupling reagent HATU and NMM as the base to produce the desired gallinamide A analogs **346**–**351** with excellent yields.

**Figure 16:**
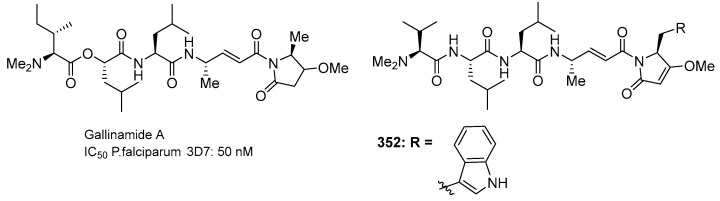
Structure of gallinamide A and synthetic analog **352**.

**Scheme 33:**
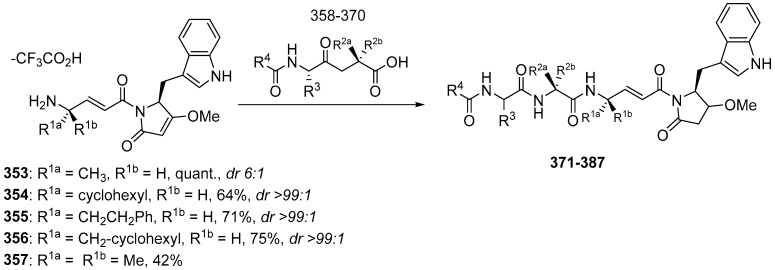
Synthesis of compounds **371**–**387**.

The natural product gallinamide A (Figure 16) was also synthesized by Stoye et al. (2019) and possesses potent inhibitory activity against *P. falciparum* cysteine proteases, namely falcipain, and therefore shows promise as a potential malaria treatment to breakdown hemoglobin in the parasitic food vacuole [28]. Gallinamide A was synthesized using NMM in a solution of the imide fragments **353**–**354** (1.0 equiv, as the trifluoroacetate salt), tripeptide **334**–**336** (1.5 equiv, as the trifluoroacetate salt), HATU and HOAt in DMF/CH_2_Cl_2_ (1:1 *v*/*v*). After consumption of the starting material (as evidenced by LC-MS), the solvent was subsequently removed by an N_2_ stream and the residue was purified by preparative RP-HPLC (Scheme 33). This synthesis produced different yields of gallinamide A analogs **371**–**387** (Table 8 and Table 9).

#### 3.2.3. PfSERA5 Analogs

PfSERA5 is an abundant asexual antigen that can inhibit parasitic growth in vitro and is a candidate malaria falciparum vaccine. Kanodia et al. (2014) reported the design and synthesis of peptides with similar sequences to the SERA5 protein, an inhibitor of malaria parasite development [29]. The solid-phase synthesis of all peptides was initiated on Rink amide resin. The appropriate Fmoc-amino acid was dissolved in a solution of TBTU/HOBT and DIPEA before being added to the resin. Fmoc was deprotected by piperidine in DMF and then acetylated by a mixture of acetic anhydride and DIPEA. The nine SE5 P1-P9 (**388**–**396**) were cleaved from the resin using TFA/H_2_O/TIS/Thioanisole/Phenol and their sequences are shown in Table 10.

#### 3.2.4. Angiotensin

Silva et al. (2015) investigated angiotensin II, a peptide that has antiplasmodial activity, as an antimalarial drugs [30,31]. They synthesized 10 peptides (**397**–**406**) (Table 11) via manual solid-phase synthesis [30]. The Fmoc strategies were applied using Wang resins and deprotection was performed by treatment with 4-MePip in DMF. Couplings were conducted using DIC/HOBt in DCM/DMF (1:1, *v*/*v*) and were monitored using the Kaiser ninhydrin test. Dry-protected peptidyl resin was exposed to TFA/H_2_O/anisole (95:2.5:2.5, *v*/*v*/*v*) for 2 h at room temperature to produce the crude linear peptides. All crude peptides were precipitated with anhydrous diethyl ether, separated from the ether–soluble reaction components by filtration, extracted from the resin with 60% ACN in water, and lyophilized. They were purified by preparative RP-HPLC in 0.1% TFA/60% ACN in water on a Waters Associates system (Delta Prep 600). The peptides were loaded onto a Phenomenex C18 (21.2 × 250 mm, 15 µm particle size, 300 Å pore size) column at a flow rate of 10.0 mL/min and eluted using a linear gradient (slope 0.33% B/min) of TFA/ACN with detection at 220 nm. Selected fractions containing the purified peptides were pooled and lyophilized.

**Figure 17:**
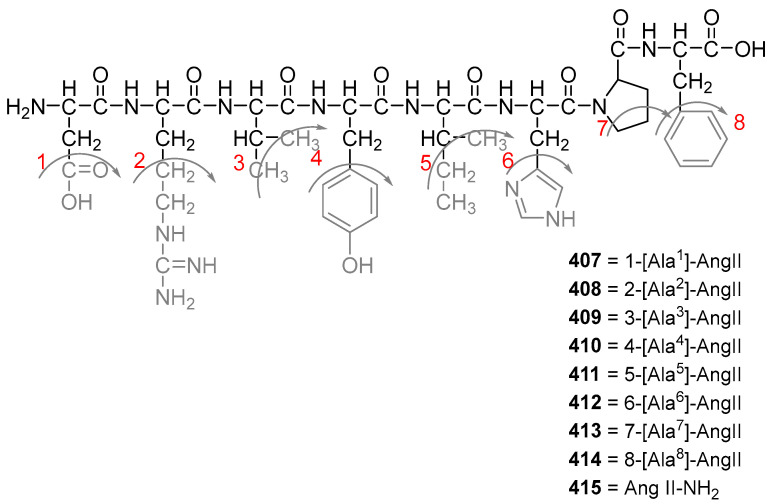
Structure of compounds **407**–**415 [31]** The additional amino acids on the native Ang II sequence are highlighted in red for each analogs.

In 2015, Silva et al. also synthesized nine octapeptides (**407**–**415**) of the renin-angiotensinsystem (RAS), which have been reported to have anti-plasmodium activity towards *P. gallinaceum* (88% sporozoite inactivation) [31]. The angiotensin II analogs (Figure 17) were synthesized by Fmoc/tert-butyloxycarbonyl (t-Boc) strategy on a solid phase, purified by liquid chromatography, and characterized by mass spectrometry. The amino acid Nα-terminal protecting group was removed with TFA in DCM in the presence of 2% anisole for 20 min. Coupling and deprotection reactions were carried out using the same reagent as before. Repeated couplings were performed for one hour using TBTU with DIPEA in DCM/NMP. Dry protected peptidylresin was exposed to 70% TFA/20% TFMSA in 10% anisole for 12 h. All crude peptides were precipitated with anhydrous diethyl ether, separated from the ether–soluble reaction components by filtration, and the fractions were lyophilized using 60% ACN (acetonitrile) in water.

Ten angiotensin analogs (**416**–**425**) were also synthesized by Torres et al. (2015) (Table 12) [32]. These lactams and sulfide bridge-containing peptides were synthesized manually by the solid-phase method using a t-Bu strategy with chloromethylated resin and a Fmoc strategy with Wang resin. Deprotection was conducted with TFA and DCM and the addition of 2% anisole. The resin was washed with anisol in isopropyl alcohol and TEA in DCM and MeOH, and the reaction was monitored with the Kaiser test. Recoupling and deprotecting the protective groups on each amino acid used the same reagents as before. Furthermore, the cyclization reaction was conducted with excess Castro reagent and DIPEA dissolved in DMSO/NMP, after which the resin was washed with TEA in DCM to obtain a dry peptidyl-resin.

Deprotection of the sulfide bridge analogs was conducted by adding 4-MePip in DCM. The amino acid coupling reaction involved treating the protected amino acid acyl-resin with a molar excess of the Boc/Fmoc protected amino acid using the DIC/HOBt reagent. The disulfide bridges were formed by first dissolving the peptide in an acetic acid solution containing iodine. The peptides were then lyophilized after being extracted with water and diethyl ether and purified by RP-HPLC.

Silva et al. (2017) synthesized eight angiotensin II hormones (**426**–**433**) (Table 13) [33]. They optimized the synthesis of these peptides for their antiplasmodial activity and to reduce their vasoconstriction and rapid degradation characteristic. The peptides were synthesized by manual solid-phase synthesis and characterized by circular dichroism spectroscopy. The synthesis was similar to previously reported and involved manual phase peptide synthesis using the Fmoc strategy and Wang resin. The amino acid residues were coupled using the DIC/HOBt reagent in DCM. Each step was followed by alternating washing with DMF, methanol, and DCM to change the degree of resin swelling and promote the removal of excess reagents. Dry peptidyl resin was added to TFA/water/anisole to obtain unprotected peptides. The S-S bonds were formed by first dissolving the crude peptide in an iodine-containing acetic acid solution. The peptides were extracted with water and diethyl ether, evaporated, and then lyophilized.

#### 3.2.5. Decoralin

Torres et al. (2018) reported the re-engineering of a wasp venom peptide, decoralin, into a synthetic anti-malaria agent through modifications that removed its hemolytic activity [34]. Dec-NH_2_ and eight analogs (**434**–**441**) were synthesized and designed to preserve specific physicochemical structures. Certain amino acid substitutions significantly improved the antiplasmodial activity, giving new sequence principles for creating potent anti-malaria drugs such as replacing the original sequence by Arg, Phe and Trp. The synthesis involved a solid-phase method and purification by chromatography and characterization using MS. The antiplasmodial activities were assessed by fluorescence microscopy. All residue substitutions resulted in increased anti-Plasmodium activity (Table 14), with [Arg]^1^-Dec-NH_2_, [Pro]^4^-Dec-NH_2_, and [Phe]^2^-Dec- NH_2_ being the most active peptides tested. 

#### 3.2.6. Carmabin and Dragomabin

**Scheme 34:**
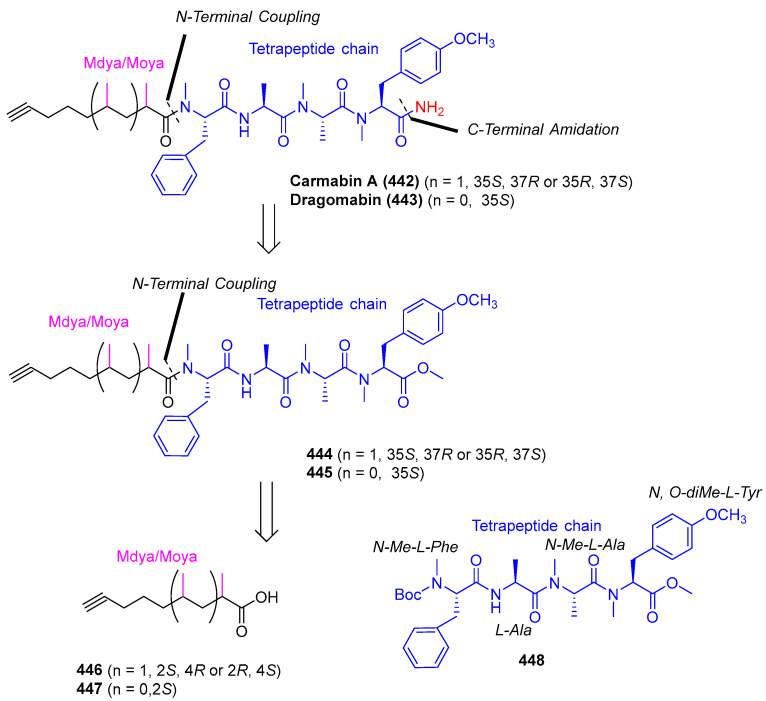
Retrosynthetic analysis of carmabin A (**442**) and dragomabin (**443**).

**Scheme 35:**
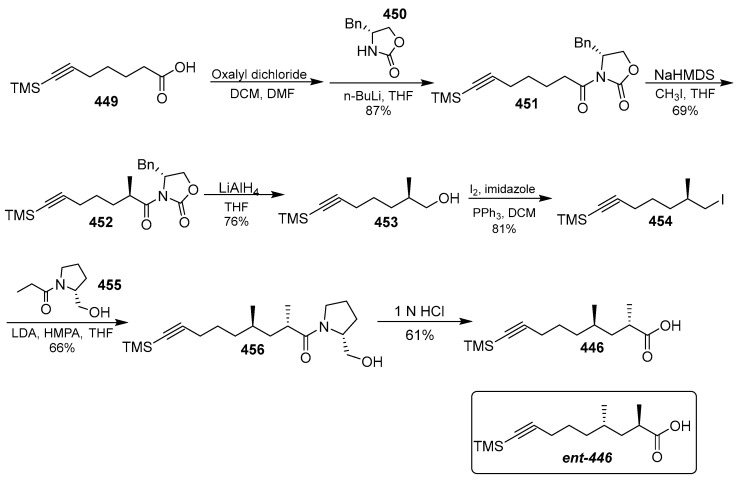
Synthesis of Mdya **446**.

**Scheme 36:**
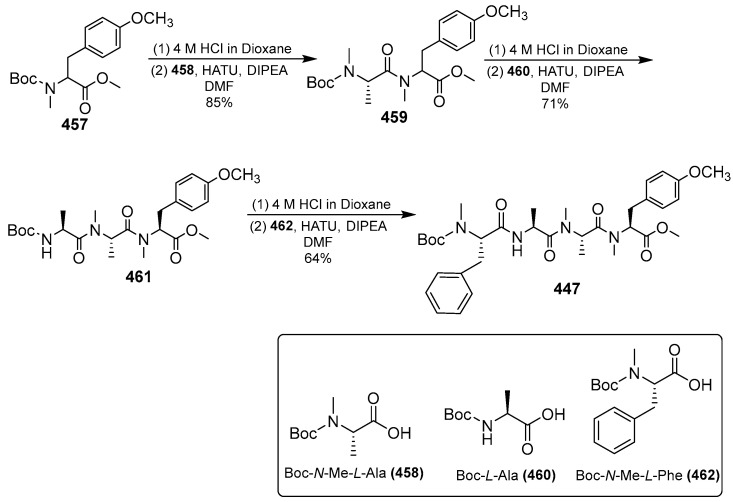
Synthesis of tetrapeptide **447**.

Ye et al. (2018) synthesized carmabin A and dragomabin, which demonstrated antimalarial activity [35]. Carmabin A and dragomabin synthesis was initiated by retrosyntethic analysis (Scheme 34). The C-terminal amide of carmabin A (**442**) and dragomabin (**443**) was prepared via amidation of the C-terminal methyl esters of compounds **444** and **445**. Compounds **448** were further disconnected into two parts: the Mdya/Moya fragment and protected tetrapeptide **454** (Scheme 35 and Scheme 36).

Tetrapeptide **44**8 was prepared by repeated condensation of amino acids (Scheme 35). The methyl group of Mdya/Moya **446**/**447** could be stereoselective. Carboxylic acid **454** was converted to the corresponding alcohol **453** in four steps involving acyl chlorination, amidation with benzyl-2-oxazolidinone, diastereoselective-methylation, and reduction. The addition of carboxylic **454** with N HCl led to the hydrolysis of the TMS group and the amide bond to yield compound **446**. 

Scheme 36 shows the construction of tetrapeptide **447**, the treatment of compound **457**, and TFA resulting in Boc deprotection. The condensation of compound **458** with the coupling reagent HATU/DIPEA produced compound **459** and repeated condensation under the same conditions yielded tetrapeptide 447. 

**Scheme 37:**
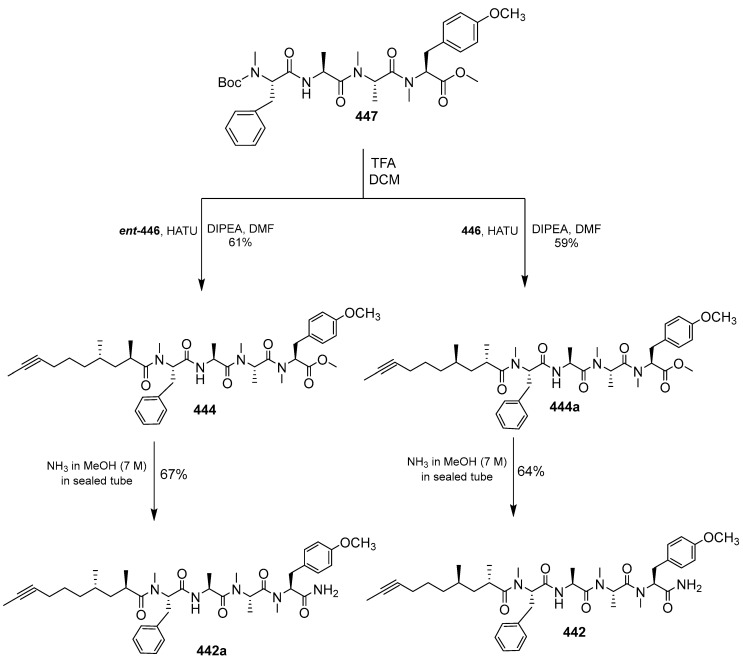
Total synthesis of carmabin A (**442**).

**Scheme 38:**
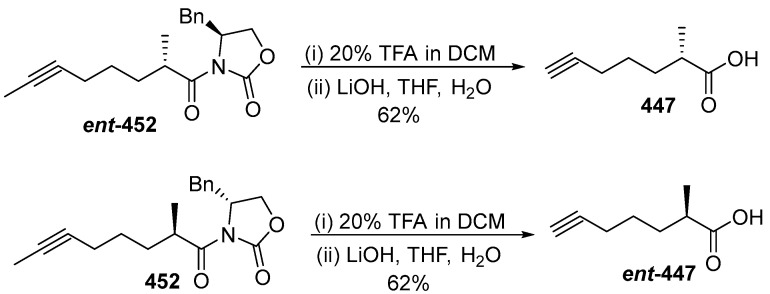
Synthesis of Moya **447** and ent-**447**.

Carmabin A was synthesized from the building blocks **446**, ent-**446**, and **447** (Scheme 37 and Scheme 38). The coupling reaction between Boc-deprotected **447** and **446** and ent-**446** was followed by the addition of ammonia to produce compounds **442** and **442a**. The configuration of carmabin A was further investigated using NMR, showing that only the **442** NMR data matched that of natural carmabin A.

**Scheme 39:**
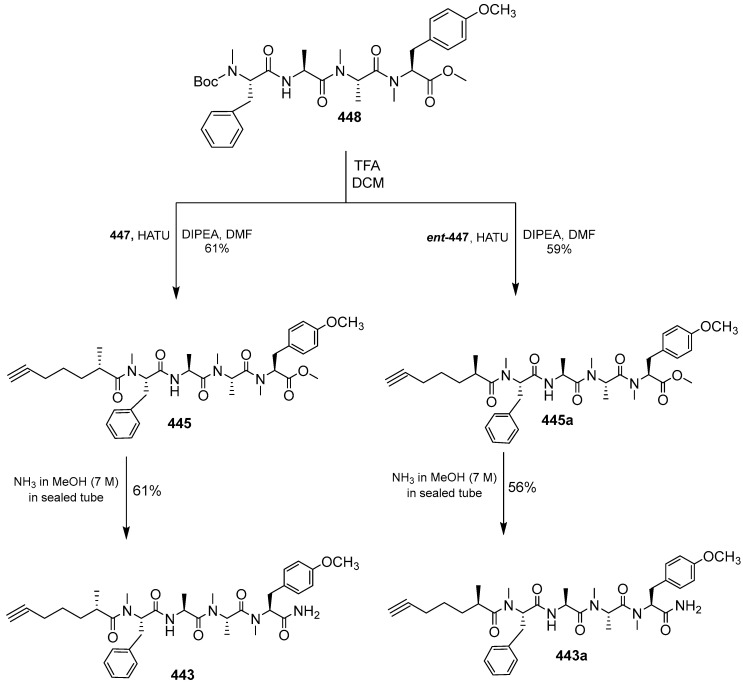
Total synthesis of dragomabin (**443**).

Dragomabin was synthesized using the building blocks Moya **447** and ent**-447**. First, Moya **447** is generated from ent**-452** by deprotecting with TMS and TFA, then the chiral auxiliary is removed (Schema 38). Dragomabin is obtained by deprotecting 448 with TMF under DCM to provide **445**, which is amidated with NH_3_ to form compound **443**. The correct structure for dragomabin was revised as shown in **443a** (Scheme 39). As dragomabin and dragonamide differ in the stereochemistry on the Moya fragment, the stereochemistry of the alkyne fragment in these lipopeptides is varied, and correlation with other natural products is not reliable. The absolute stereochemistry at C35 and C37 of carmabin A was assigned as 35R, 37S and the absolute stereochemistry at C35 of dragomabin has been revised as 35R.

### 3.3. Biology Activity of Isolated and Synthesized Compounds

Ribifolin (**1**) is moderately effective against the Plasmodium falciparum 3D7 strain with an IC_50_ of 42 µM (Table 15), while its linear analogue 1a had an IC_50_ of 519 µM, demonstrating the importance of cyclization to enhance the biological activity [3]. None of the tested compounds showed cytotoxic potential against human cells (HEK293aT), although normal growth was observed in the concentration range of 0.001–100 μM.

The isolated cyclopeptide alkaloids (**2**–**5**) from the root bark of Hymenocardia acida demonstrated antiplasmodic activity against *P. falciparum* K1, with IC_50_ values ranging from 12.2 to 27.9 μM. Only hymenocardine (**2**) exhibited cytotoxic properties against MRC-5 cells with an IC_50_ of 51.1 ± 17 μM (Table 16) [4].

The antiplasmodic activity of nummularine-R (**6**), O-desmethylnummularine-R (**7**), hemsine-A (**9**), ramosine-A (**11**), and oxyphylline-F (**14**) was evaluated against the *P. falciparum* strain KI, while cytotoxicity was tested on MRC-5 cells (human fetal lung fibroblast cells). Based on the structural features and IC_50_ values for compounds **6**, **7**, **9**, and **11**, it can be assumed that the tryptophan moiety in the side chain is important for antiplasmodic activity. Compound **14** does not contain a tryptophan unit and also showed antiplasmodic activity, indicating that tryptophan could mediate, but is not vital for the antiplasmodic activity. The most promising compound was O-desmethylnummularine-R (**7**), which exhibited an IC_50_ of >64.0 µM against MRC-5 cells (Table 17) [5].

**Figure 18:**
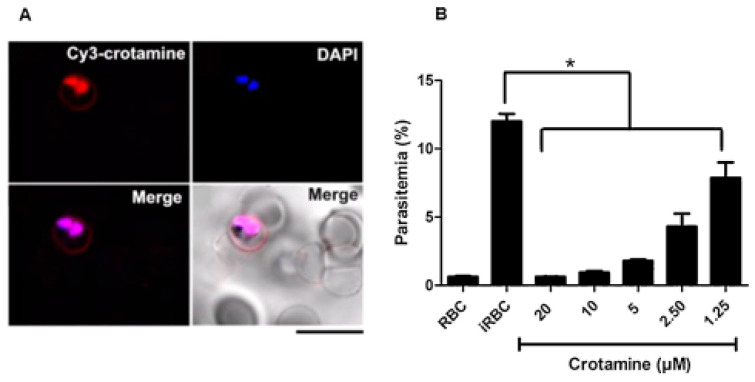
Uptake of crotamine (**15**) by parasite-infected RBCs and effect on *P. falciparum* survival in vitro. (**A**) *P. falciparum* culture was incubated (10 µM) for 1 h, at room temperature. (**B**) Crotamine was added to 20, 10, 5, 2.50, and 1.25 µM *P. falciparum* culture. * *p* < 0.05.

Maluf et al. (2016) reported that crotamine (**15**) selectively enters infected erythrocytes (Figure 18A) and has potent anti-plasmodial activity with an IC_50_ value of 1.87 µM (Figure 18B) [6]. 

**Figure 19:**
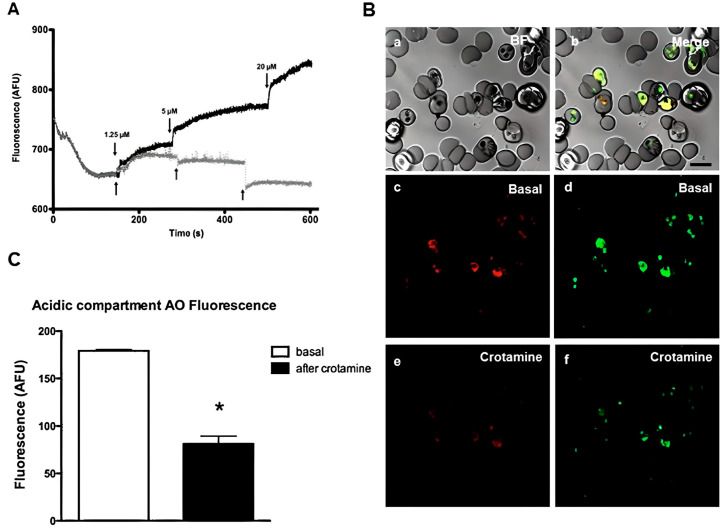
Effect of chloroquine and crotamine (**15**) on intracellular acridine orange (AO) mobilization from acidic compartments of isolated *P. falciparum* parasites. (**A**) The lysosomotropic fluorochrome AO (5 M) were added in isolated parasites solution. Different concentrations of crotamine (1.25, 5, and 20 µM) were added during AO fluorescence acquisition in spectrofluorometer cuvette. Fluorescence intensities (arbitrary fluorescence units—AFU) represent at least three different cell preparations. Crotamine (gray line) and chloroquine (black line). (**B**) The alterations of acridine orange (AO) fluorescence was performed in confocal microscopy. iRBC was loaded with 5 µM of AO during 30 min. After addition of 20 M of crotamine, the effects on fluorescence of AO were monitored. (**a**–**f**) Confocal images: (**a**) Brightfield (BF); (**b**) Merged; (**c**) basal AO red channel; (**d**) basal AO green channel; (**e**,**f**) AO red and green channels after crotamine addition; (**C**) Histogram of AO fluorescence (red channel) with mean ± SD (N = 3). * *p* < 0.05.

The instability of H^+^ homeostasis by cr otamine was also confirmed (Figure 19B,C). The authors suggest that crotamine alters the internal pH of the vesicle due to its abundant Lys and Arg residues and the resulting high net charge (8+). Internal pH regulation is important for parasite survival, as it regulates the activity of certain intracellular enzymes required for parasite growth. Thus, this polypeptide is a promising lead molecule for the development of potential new peptidomimetics that have selectivity for infected erythrocytes and the ability to inhibit malaria infection by their natural affinity for acid vesicles [6].

The cyclic octadepsipeptides, octaminomycins A (**16**) and B (**17**) showed activity against *P. falciparum* (Table 18). According to Jang et al. (2017), compounds **16** and **17** were not significantly cytotoxic at a concentration of 30 μM against human cervical cancer cells (HeLa), human promyelocytic leukemia cells (HL-60), mouse temperature-sensitive cdc2 mutant cells (tsFT210), and rat kidney cells, which were infected with ts25 (srcts-NRK). They were also evaluated for antimicrobial activity against Staphylococcus aureus 209, Escherichia coli HO141, Aspergillus fumigatus Af293, Pyricularia oryzae kita-1, and Candida albicans JCM1542, as well as antimalarial activity against the *P. falciparum* 3D7, Dd2, and K1 strains. Chloroquine was less effective against strains resistant to Dd2 and K1, whereas compounds **16** and **17** showed the same in vitro antimalarial activities against chloroquine-sensitive 3D7 and chloroquine-resistant Dd2 and K1 strains, with no antimicrobial activity up to 30 μM [7].

The antimalarial activity of the isolated kakeromamide B peptide (**18**) was evaluated against asexual blood-stage and liver-stage *P. falciparum* (Table 19). Compound **18** exhibited moderate activity against the blood stage of *P. falciparum* with an EC_50_ value of 8.9 µM as well as moderate liver-stage antimalarial activity against *P. berghei* liver schizonts with EC_50_ values of 11 µM. Although **18** displayed only moderate antimalarial activity, its ability to inhibit both the blood and liver life stages of Plasmodium, coupled with its low cytotoxicity in human cell lines, make it a promising lead compound for drug discovery [8].

**Figure 20:**
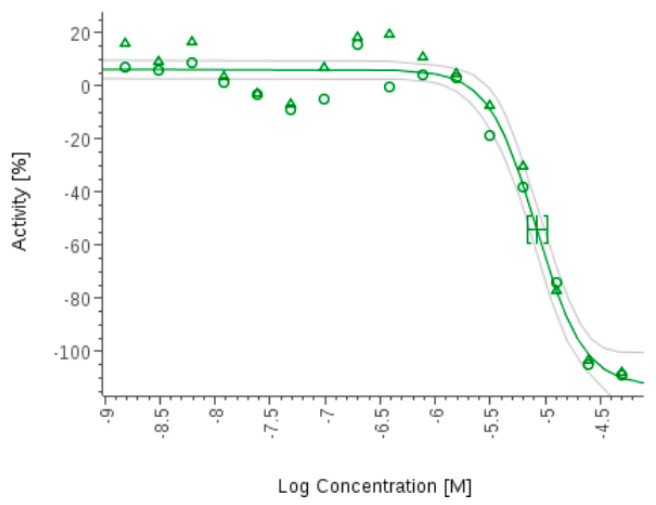
Pipecolisporin (**19**) inhibits the growth of *Tripanosoma cruzi*.

**Figure 21:**
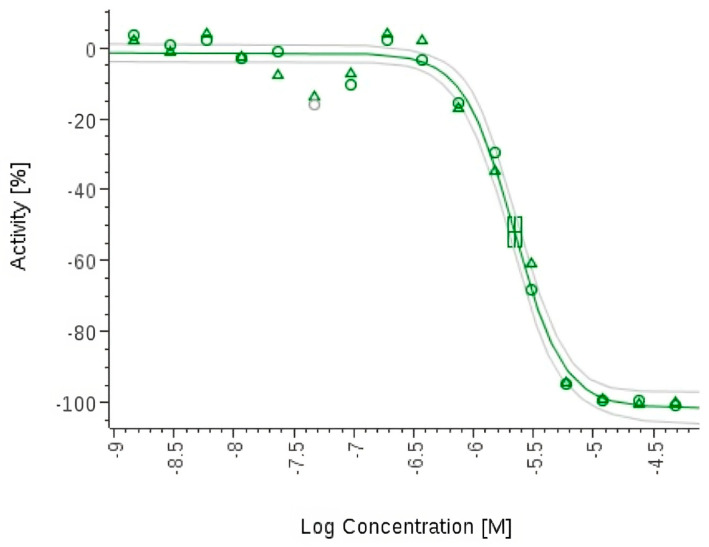
Benznidazole inhibits the growth of *Tripanosoma cruzi*.

**Figure 22:**
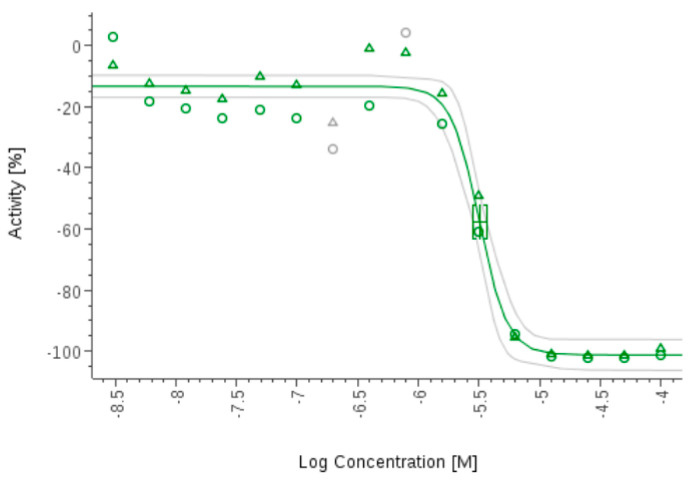
Pipecolisporin (**19**) inhibits the growth of *P. falciparum* 3D7.

**Figure 23:**
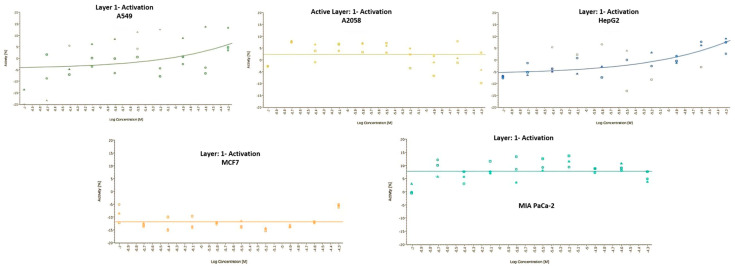
Growth inhibition curves of pipescoliporin (**19**) against human cell lines.

The novel cyclic antimalarial and antitrypanosomal hexapeptide, pipecolisporin (**19**), was isolated from cultures of Nigrospora oryzae CF-298113 and exhibited interesting activity against *P. falciparum* and *Trypanosoma cruzi* Tulahuen C4 parasites [9]. The activity against the *T. cruzi* Tulahuen C4 parasites was the most remarkable, with an IC_50_ of 8.46 µM, comparable to that of the standard drug benznidazole, currently used in the treatment of Chagas disease (IC_50_ in the same assay of 2.21 µM) (Figure 20 and Figure 21). The activity against *P. falciparum* was also in the micromolar range, with an IC_50_ of 3.21 µM (Figure 22). It was not cytotoxic to the human cancer cell lines A549 (lung carcinoma), A2058 (metastatic melanoma), MCF7 (breast adenocarcinoma), MIA PaCa-2 (pancreatic carcinoma), and HepG2 (hepatocyte carcinoma) at the highest concentration tested of 50 µM (Figure 23). 

Koshidacins A (**20**) and B (**21**) demonstrated in vitro antiplasmodial activity and cytotoxicity against human MRC-5 cells [10]. Compounds **20** and **21** demonstrated antiplasmodial activity against a chloroquine-sensitive *P. falciparum* FCR3 strain with IC_50_ values of 17.1 and 0.89 μM, respectively. Similarly, compounds **20** and **21** also exhibited antiplasmodial activity against chloroquine-resistant *P. falciparum* K1 strain with IC_50_ values of 12.5 and 0.83 μM, respectively. Compounds **20** and **21** showed cytotoxicity against human MRC-5 cells, with IC_50_ values of 6.8 and 14.7 μM, respectively, suggesting selectivity indices ranging from 0.4 to 18.4 (Table 20). In addition, when given intraperitoneally at a dose of 30 mg/kg/day for 4 days, compound **21** inhibited 41% of malaria parasites in vivo (Table 21). 

Shi et al. (2018) evaluated georatusin (**22**) produced by a soil fungus Geomyces auratus [11]. It had no obvious cytotoxicity, but displayed antiparasitic activities against Leishmania donovani (IC_50_ = 9.1 μM) and *P. falciparum* (IC_50_ = 1.6 μM) (Table 22). This discovery offers new insight into the metabolic potential and ecological importance of Geomyces and may encourage further exploration of this genus.

New highly N-methylated linear peptides, friomamaride B (**23**) and shagamides A-F (**24**–**29**), exhibited activity against three strains of blood-stage *P. falciparum* [12]. All compounds were tested for their effectiveness against three blood-stage of *P. falciparum* using the blood-stage antiplasmodial and cytotoxicity assay. Friomamaride B (**23**), shagamides C (**26**) and D (**27**) with values less than 10 µg/mL all possess potential activity. The N-terminal of phenylalanine residue is essential for this activity. None of the isolated compounds demonstrated cytotoxic activity.

The carboxypeptidase inhibitor NpCI peptide (**30**), which is related to the model enzymes of bovine carboxypeptidase A (bCPA) and porcine carboxypeptidase B (pCPB), was discovered by Cabrera-Muñoz et al. (2023) [13]. The kinetic characterization of NpCI revealed that it was a slow inhibitor of bCPA and pCPB. While pCPB inhibition was not significant, the evaluation of NpCI inhibition was also performed by comparing the decrease in bCPA inhibitory activity caused by the substrate with the increase in substrate concentration. The Dd2 strain was used for in vitro antiplasmodial activity against *P. falciparum*, showing that the cycle was significantly delayed with an IC_50_ of 5.5 µmol/L. Parasite growth can be slowed down by increasing inhibitor concentration. The growth inhibition by NpCI occurs during parasite development. The 3D7 strain displayed comparable inhibition with a delayed maturation mechanism. NpCI was not cytotoxic to human cells (IC_50_ < 25%).

Kiefer et al. (2019) synthesized 17 cyclomarin analogues (**96**–**100**, **106**–**110**, **119**–**125**) and evaluated their biological activities against chloroquine-sensitive Pfalcp strain 3D7 and multi-resistant strain Dd2, as well as against Mtb wild-type strain Erdma (Table 23) [17]. The antitubercular activity was also evaluated, and the resazurin reduction microtiter assay (REMA) was used to measure the growth inhibition of Mtb.

Three compounds (**96**, **97**, **99**) demonstrated excellent parasitic growth suppression (Table 23). Compound **99** consists of a simplified γ,δ-unsaturated side chain, and the N’-methyl tryptophan unit. Desoxycyclomarin C, which was inspired by a natural product compound, has a similar range of bioactivity, but it is significantly shortened. The anti-mycobacterial activity was demonstrated by the five derivatives (**96**, **98**, **99**, **108** and **110**) when applied to Erdman wild-type strain. Due to a remarkable simplification by replacing two of the four non-canonical amino acids by L-valine and L-tryptophan, compound **110** represents a highly appealing natural product-derived lead structure for battling Mtb.

Fagundez et al. (2018) revealed that most of the synthesized peptides have the potential to be antimalarials [20]. The corresponding cyclopeptide effectiveness was evaluated against the chloroquine-resistant K1 strain of *P. falciparum*. Cyclo-Cys(Trt)-Gly-Thr(tBu)-Gly-Cys(Trt)-Gly (compound **140**) showed potent in vitro and selective activity against this parasite with an EC_50_ = 28 µM. The inclusion of a carboxylic group from Glu (**151**, **152**) can increase solubilization, and the substitution of hydrophobic amino acids can boost biological activity (Table 24) [20].

Fagundez et al. (2018) also developed cyclopeptides containing N-methyl amino acids that demonstrated promising antiplasmodial activity, compounds **156**–**161** and **163**–**165** (Table 25) [21]. In addition, a new class of antimalarial cyclopeptides that contain N-methyl Gly has been developed that exhibits enhanced antiplasmodial activity. The in vitro evaluation of the compounds against *P. falciparum* revealed that N-Me-Gly is required to maintain the activity in the presence of a Glu with a free carboxyl group. Moreover, none of the active compounds are toxic against HepG2 cells.

**Figure 24:**
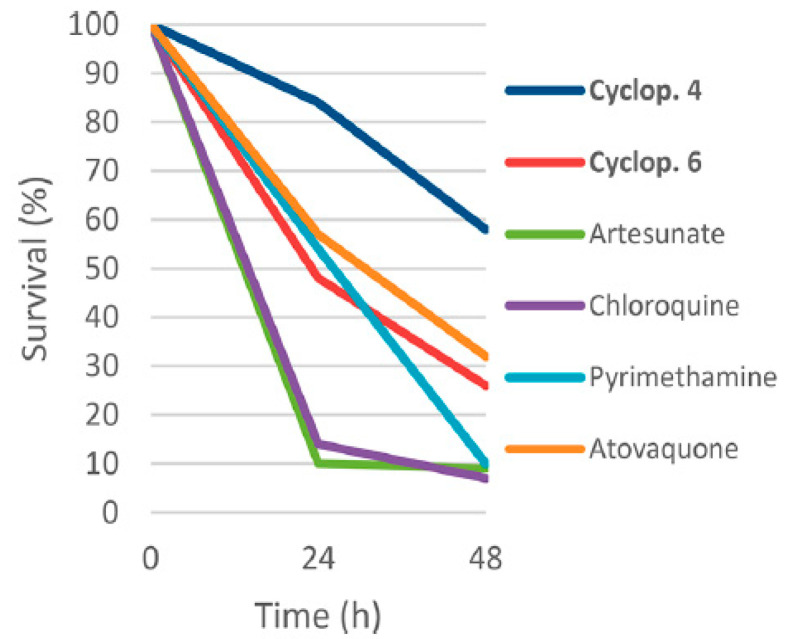
Killing profile against *P. falciparum* 3D7A strain of compounds **158** and **160**, artesunate, chloroquine, atovaquone, and pyrimethamine tested at 10 times the EC_50_ (number of replicates *n* = 2). cyclop. 4 = **158**; cyclop. 6 = **160**.

Compounds **158** and **160** were assessed for their antimalarial activity in comparison to other antimalarial drugs (Figure 24). These results point to an antimalarial mode of action that does not immediately affect parasite viability. The prophylactic potential of compounds **158** and **160** is demonstrated by their low and submicromolar EC_50_ values (0.018 and 0.355 µM) in the liver stage of the parasite.

Compounds **157** and **165** are effective against *P. berghei* parasites, reducing parasitemia by 71 and 66% on day 5. To evaluate the oral bioavailability, the plasma pharmacokinetic of compound **157** in male Swiss Albino mice, following a single oral dosage, was examined, and shows a considerable half-life of 4.93 h. 

The hirsutellide A analog, compound **177**, exhibited moderate antiplasmodial activity (IC_50_ = 2.3 μM) similar to that reported for hirsutellide A (IC_50_ = 4.2 μM). Compound **177** is not cytotoxic (IC_50_ > 100 μM) to Hep2G cells. ADME profiling for compound **177** displayed moderate stability in humans, but low stability in mouse microsomes (Table 26) [22].

Additionally, the analogs have little to no activity against Mtb H37Rv. Peptide analogs generally have higher antiplasmodial activity (IC_50_ = 1.8−7.7 μM) than the depsipeptide analogs (IC_50_ = 7.5−20.1 μM), exhibiting a higher aqueous solubility, a high plasma stability and mouse plasma stabilities. Thus, the ester-to-amide substitution and the membrane permeability of hirsutellide A analogs appear to be dependent on the nature of the amino acid substituents. This study provides insight into the structural features relevant to the cyclic peptide-related drugs. Some compounds exhibited promising antimycobacterial activity and low cytotoxicity, making them potential candidates for further research and development as anti-TB agents.

The developed macrocyclic peptides as proteasome inhibitors has potential for antimalarial drugs (Zhang et al., 2022) [24]. Cyclic peptide **201** is a noncovalent inhibitor with strong antimalarial activity and high species selectivity, but has poor pharmacokinetics, therefore a docking model was developed. Compound **201** was acetylated using the N-Terminal amino group to boost its antiparasitic and inhibition activities. Five compounds were created, and P1, P3, P5, and P2–P4 linkers were examined for their contribution to the potency and drug-like properties of macrocyclic peptides (Table 27, Table 28, Table 29 and Table 30) [24]. 

Compound **220** demonstrated good potency against parasite and pharmacokinetics and made optimal use of P1 amide moiety modification (Table 28). Compound **223** showed moderate inhibition against hu-β2c (IC_50_ = 97.4 μM). Compounds **272** and **270** exhibited moderate inhibition of β1c and β1i. All other compounds showed <50% inhibition against all the four subunits, even at 100 μM.

Similar to the observation of the simultaneous inhibition of β5 and β2 in tumors, co-inhibition of β5 and β2 is synergistic and the inhibition of Pf20s β5 is sufficient to kill Plasmodium. The compounds were optimized for aqueous solubility, passive membrane permeability, metabolic stability, and a clean off-target profile against CYP450s and the hERG channel. The proteasome function appears to be critical for parasites to survive. 

Falcitidin and its analogs (**296**–**303**) were synthesized by Kotturi et al. (2014) and showed moderate activity against *P. falciparum* 3D7, but only when N-tritylated on its histidine residue [25]. The IC_50_ activity of the new compounds, which ranged from 1 to 5 µM, was typically modest in whole cells. Compound **302** exhibited the highest activity (IC_50_ 0.14 µM). These new compounds represent an important new peptide chemotype that may be elaborated into improved antimalarial leads (Table 31).

Conroy et al. (2014) developed a new class of antimalarial drugs based on the natural product gallinamide A, which inhibits the falcipain cysteine proteases essential for malaria parasite survival [27]. These gallinamide A analogs were as effective as chloroquine against the *P. falciparum* parasite. Gallinamide A analog **309** has strong inhibitory activity against FP-2, FP-3, and *P. falciparum* in vitro (Table 32). Reducing the enol moiety in the acyl-pyrrolinone unit in compound **310** resulted in a two-fold reduction in antiplasmodial activity (IC_50_ = 210 nM), but a slight improvement in activity against FP-2 and FP-3. Analog **311** showed no measurable inhibition of FP-3 and a 3 orders of magnitude drop in inhibitory effectiveness against FP-2 (IC_50_ = 3710 nM), as well as significantly decreased activity against P.falciparum. Analog **312** lost the measurable inhibitory activity against the FPS and the parasite after losing both of its olefinic moieties.

The activities of analogs **313**, **315**, and **317** were similar: they inhibited FP-2 at low micromolar concentrations (IC_50_ = 3.4–11.5 µM), did not significantly inhibit FP-3 at 25 µM, and inhibited *P. falciparum* at nanomolar concentrations (IC_50_ = 320–5400 nM) (Table 33). Interestingly, peptide **318** demonstrated less potent antiparasitic activity (IC_50_ = 6.6 µM) but inhibited FP-2 and FP-3 by adding an N-methylproline functionality at the N-terminus of the peptide while keeping the C terminal benzylamide. The lack of activity was especially noticeable for **315** because it has the same structure as analog **309**. The addition of a more flexible and highly functionalized hydroxyproline methyl ester to the C-terminus in **314** produced inhibitory activity against FP-2 and *P. falciparum*, which was comparable to the benzylamide-derived compounds. Low micromolar antiparasitic activity (IC_50_ = 1.1 µM) and moderate inhibitory activity against FP-2 and FP-3 were achieved by C-terminal functionalization as a thiazole amide in compound **316 [27]**.

Like analog **309**, the C-terminal *N*-acyl-pyrrolinone in **346**–**351** restored strong inhibitory activity against the FPs and *P. falciparum* (Table 34). Compound **346** displayed similar inhibitory activity to **309** against FP-2, FP-3, and *P. falciparum* despite having no modification on the pyrrolinone molecule. The pyrrolinone ring of **347** was modified to include a more hydrophobic substituent, which improved its inhibitory efficacy against FP-3 and *P. falciparum*. As with compound **347**, the addition of aromatic side chains to the pyrrolinone ring did not modify the inhibitory activity against FP-2 but significantly increased activity against FP-3 and *P. falciparum*. The most effective inhibitor of FP-3 and *P. falciparum* was compound **349**, which had an indole side chain attached to the pyrrolinone ring [27].

The compounds were screened against the *P. falciparum*, namely AP M1, AP M17, and AP M18 aminopeptidase (AP) enzymes. The effective N-acyl pyrrolinone analogs **309** and **346**–**349**, as well as the C-terminal amide derivatives **309**, **315**, and **317**, had IC_50_ values of less than 600 nM against the 3D7 strain of *P. falciparum* (Table 35). The CQ-resistant, Dd2 strain of *P. falciparum* was powerfully inhibited by every tested compounds (IC_50_ = 29.0–421 nM). The compounds were selective inhibitors of *P. falciparum* over HEK298 cells, with **309**, **315**, and **317** showing no measurable inhibition of this cell line at a concentration of 50 µM, but having strong inhibitory activity against human cathepsin [27].

The gallinamide A analogs, synthesized by Stoye et al. (2019), were also tested for activity against FP2, FP3 also CQ-sensitive 3D7 and CQ-resistant W2 strains of *P. falciparum* in vitro (Table 36) [28]. Most substitutions in the compound structure were well tolerated, except for analog **387** which had a more potent activity parasite (IC_50_ 3D7 = 1 nM; W2 = 4 nM) and substituents on R^1^ which markedly enhanced the stability in plasma and blood. Five gallinamide A analogs were assessed in vitro for their stability in mouse blood and plasma (Table 37). The half-life in plasma and blood was significantly prolonged due to the substituents at R^1^ and R^3^. Additional cytotoxicity assays in the HEK293 cell line of the compounds displayed no measurable cytotoxicity at 25 µm. The last three analogs **372**, **373** and **387** were assessed in vivo in a mouse model of cerebral malaria (CM), *P. berghei* ANKA (PbA) infection. Severe signs of disease as well as the rise in parasitemia were both significantly delayed by analog **387**.

**Figure 25:**
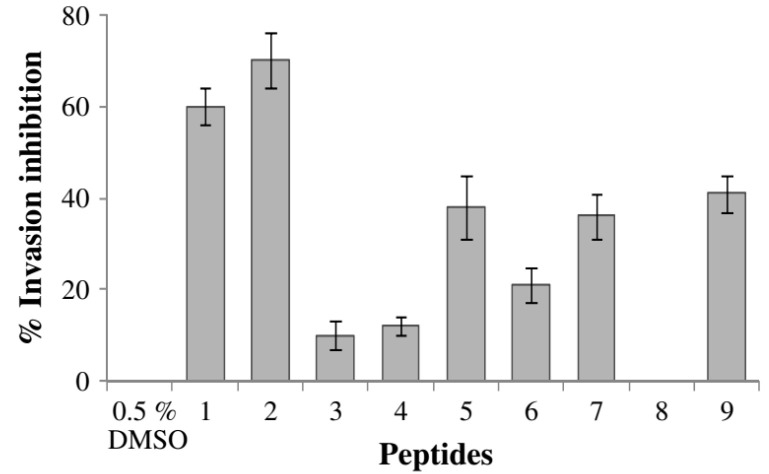
The ability of SE5 P1 (**388**) and SE5 P2 (**389**) to specifically inhibit PfSERA550 activity.

The peptides derived from PfSERA5 by Kanodia et al. (2014) inhibited the enzymatic activity of PfSERA5P50 protein, which in turn blocked the development of the parasites in an in vitro culture [29]. Evaluation of PfSERA5 derivative against *P. falciparum* development and growth revealed that SE5 P1 (**388**) and SE5 P2 (**389**) have the highest parasite growth inhibition and invasion values of 60–70% (Figure 25). 

Analysis of the synthetic substrate Suc-LLVY-AMC revealed that PfSERA550′s proteolytic activity was greatly reduced by the two C-terminal peptides SE P1 (**388**) and SE5 P2 (**389**). Kanodia et al. also describe how they used computational modeling to examine molecular docking studies with the known crystal structure of PfSERA5 to explore the effects of SE5 P1 (**388**) and SE5 P2 (**389**) peptides. SE5 P1 (**388**) and SE5 P2 (**389**) occupied 50.1 and 57.5% larger than the substrate when the Suc-LLVY-AMC was applied to see the bond (Table 38), suggesting that the peptides must interact with Glu638 and Ser640/Ser641 to be inhibitory [29].

*P. falciparum*-infected RBCs uptake of labeled SE5 P1 and SE5 P2 peptides when the localization of biotinylated peptides therein inhibited the protease activity similarly to the non-biotinylated peptides. This indicates that the peptides have access to the intracellular parasites and are co-localized with the PfSERA5 protein. PfSERA5 plays an important role in parasite development and the final proteolytic cleavage, which can be produced as a new drug design and offers information on the potential use of these peptides as antimalarial therapeutics [29].

Angiotensin II analogs synthesized by Silva et al. (2015) and tested in vitro to identify a short bioactive peptide as well as to verify the hydrophobic cluster’s influence on parasite-membrane interaction on both *P. gallinaceum* and *P. falciparum* [30]. Fluoresence microscopy was utilized to examine the effects of the peptides on *P. gallinaceum* sporozoites produced by the salivary glands and the therapeutic index (MHC/MIC ratio). Higher values in the therapeutic index indicate more antimicrobial specificity. It is a parameter that measures the specificity of an antimicrobial agent and is calculated by the ratio MHC and MIC. The MHC/MIC ratio, which was higher in peptide **401**, indicates that the peptides have varied specificities (Table 39). New peptides related to Ang II were designed, including the most hydrophobic amino acid residues (Val, Ile, Pro, and Phe), aromatic residues (Tyr, His, Pro, and Phe) and residues from the Ang II hydrophobic cluster (Tyr, Ile and His) in an attempt to verify the peptide–parasite interactions. 

**Figure 26:**
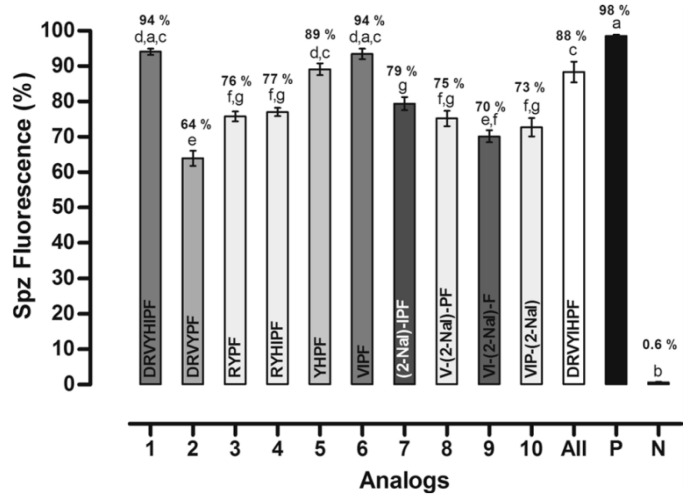
Effects of peptides on membrane permeability expressed as the percent of fluorescent mature sporozoites (mean ± standard deviation, *n* = 9). Letters indicate those results not significantly different from each other at the *p* < 0.05 level. Positive control group (+): digitonin/PBS; negative control group (−) Positive control group (+): digitonin/PBS: negative control group (−): PBS.

Due to the influence of hydrophobic clusters, side chain aromatic rings, and hydrophobic residues, these peptides showed antiplasmodial activity in *P. gallinaceum* sporozoite (64–94%) and activity between 89 and 94% (Figure 26). The three peptides with the highest antiplasmodial activity were 1 (**397**), 5 (**401**), and 6 (**402**) with 94, 89, and 94%, respectively [30].

**Figure 27:**
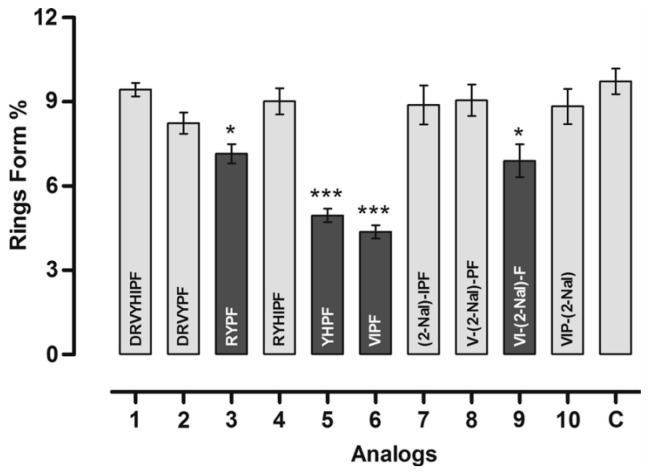
Effect of peptides in the new ring formation. * Statistically significant compared with control value *p* < 0.05. *** Statistically significant compared with control value *p* < 0.001. Analog 1 = **397**; 2 = **398**; 3 = **399**; 4 = **400**; 5 = **401**; 6 = **402**; 7 = **403**; 8 = **404**; 9 = **405**; 10 = **406**.

The effect of the peptides in the *P. falciparum* erythrocytic cycle was assessed in vitro. All peptides reduced new ring formation at 10^−8^ mol L^−1^, which was studied by Saraiva et al. as the ideal concentration for these inhibition assays. Four analogs reduced the ring formed in the blood stage, but only analogs 5 (**401**) and 6 (**402**) showed inhibition that was higher by 50% than control (Figure 27). Figure 27 showed that after 24 h of incubation with 2–3% schizont-infected erythrocyte cultures in the absence (control) or presence of 10*^−^*^8^ mol L*^−^*^1^ peptides, the percentage age of rings was evaluated (* *p* < 0.05 compared to the control, *** *p* < 0.001). The result is statistically significant compared to the (mean ± standard deviation, *n* = 2), as indicated by the dark grey shading [30].

**Figure 28:**
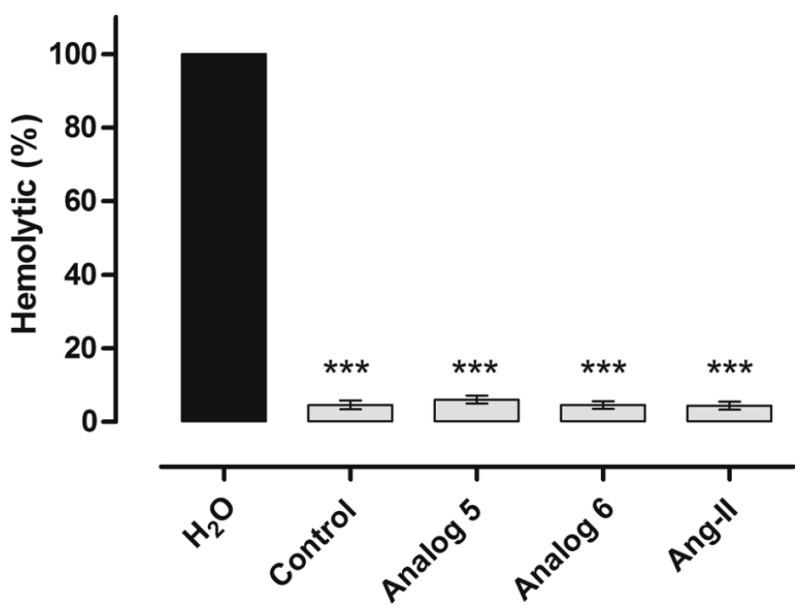
Haemolytic assay of human red blood cells. *** Statistically significant compared with distilled water value *p* < 0.05. Analog 5 = **401**; 6 = **402**.

Another technique for determining the hemolytic effect of peptides demonstrated that peptides **401** and **402** had an effect of the Ang II to define the hemolysis. These peptides did not exhibit hemolytic effects (Figure 28).

**Figure 29:**
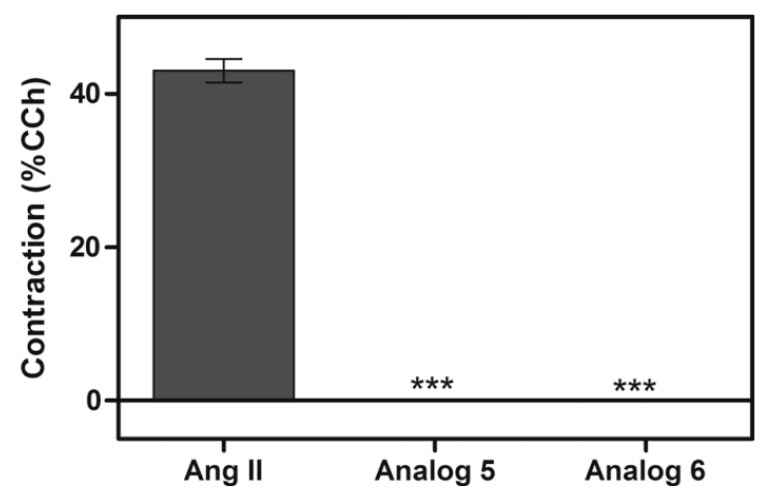
Effect of Ang II, peptide 5 (**401**) and 6 (**402**) on the contractile responses of muscle tissue compared to carbachol (CCh) activity. *** Statistically significant compared with control value *p* < 0.05 (mean ± standard deviation, n = 2)

The hydrophobic portion and the Arg, Tyr, Pro, and Phe residues increased the antiplasmodial activity when they were present in the primary sequence. Furthermore, these peptides did not display hemolysis or contractile response activities, as shown in Figure 29 (*** *p* < 0.05 compared to control, *n* = 2) [30].

**Figure 30:**
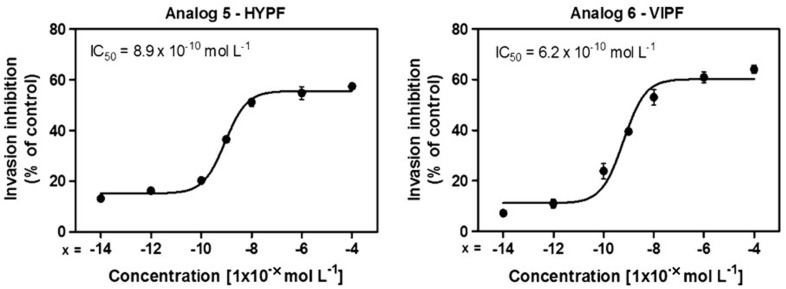
IC_50_ value for each assay. Peptide 5 (**401**) and 6 (**402**) were diluted in seven concentration [(10*^−^*^4^, 10*^−^*^6^, 10*^−^*^8^, 10*^−^*^10^, 10*^−^*^12^, 10*^−^*^14^) mol L*^−^*^1^] giving 7–65% inhibition.

The IC_50_ values are more promising than the Ang II results, which were demonstrated by evaluating seven concentrations giving 7–65% inhibition (Figure 30). The results imply that intramolecular interactions cause conformational tendencies that are crucial for antiplasmodial activity. When present on the peptide primary sequence, the hydrophobic portion and the residues of Arg, Tyr, Pro, and Phe increased the antiplasmodial activity. Following in vivo model tests of this class of peptides, this type of research aids the development of new chemotherapeutics, which can be explored as antimalarial drugs [30].

**Figure 31:**
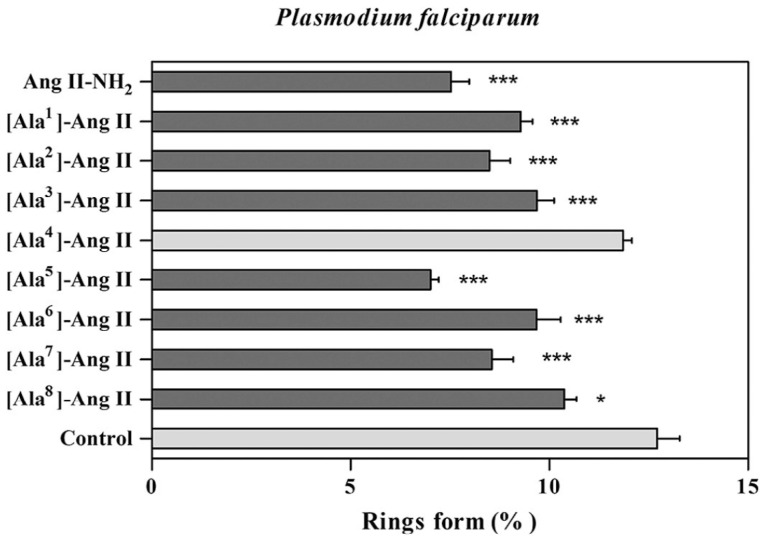
Effect of analogues in the new ring formation. * Statistically significant compared with control value *p* < 0.05. *** Statistically significant compared with control value *p* < 0.01. Dark grey shading indicates that the result is statistically significant compared with control.

The synthesized RAS octapeptides were tested for their effectiveness against *P. falciparum* and *P. gallinaceum*, revealing that only [Ala^5^]-Ang II showed equipotent Ang II activity with 45% of biological activity [31]. At 10^−8^M concentration, *P. gallinaceum* erythrocytic cycle [Ala^6^]-Ang II decreased parasite invasion by 49%. The efficiency of anti-plasmodial activity was significantly impacted by the presence or absence of amino acid substitutions. The anti-plasmodial activity of the Ang II molecule depends on specific amino acid side chains. The biological activities and binding affinities are affected when the biologically active peptide is replaced by an alanine residue. From Figure 31, the percentage of rings was determined after 24 h of incubation of erythrocyte culture infected with 2–3% schizonts in the absence (control) or in the presence of 10*^−^*^8^ M analogs. Dark grey shading denotes that the result is statistically significant compared to the control.

**Figure 32:**
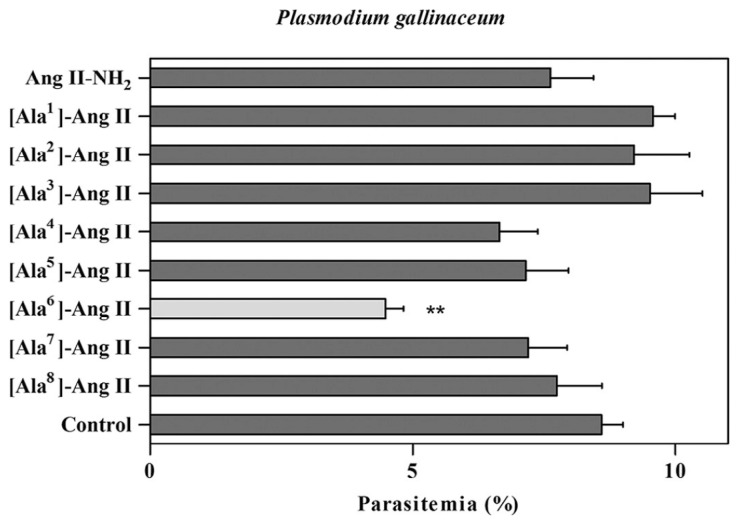
Effect of analogs in the erythrocytic cycle of *P. gallinaceum*. ** Statistically significant compared with control value *p* < 0.05. Dark grey shading indicates that the result is not statistically significant compared with control

Regarding the contractile response, [Ala^5^]-Ang II and [Ala^6^]-Ang II did not promote contractile activity compared to Ang II, and carbachol [Ala^5^]-Ang II and [Ala^6^]-Ang II reduced parasite invasion in red blood cells in the erythrocyte hemolysis assays, and presented no effect on cells integrity at 10^−8^ M concentration. This model directs new possibilities for peptide design that can act more effectively in preventing the erythrocytic cycle of the parasite and other phases of the human–malaria cycle. Figure 32 demonstrates that after erythrocyte cultures were infected for 24 h with 3% parasitemia, the percentage of total parasites was determined in the absence (control) or presence of 10*^−^*^8^ M analogs. Dark grey shading denotes that the result is not statistically significant compared with control [31].

Other synthesized angiotensin analogs presented high antiplasmodial activity in the *P. gallinaceum* test [32]. They have similar hydrophobic interactions between the isolated guanidyl (Arg^2^) and hydroxyl (Tyr^4^) groups and the side chains of alkyl and aromatic amino acid. The guanidyl (Arg^2^) and carboxyl (Asp^1^) groups seem to be less significant in this case. Lactam bridges have an important role in constricting the conformation of bioactive peptides. When evaluated against *P. falciparum*, all lactam bridge analogs lacked significant activity against *P. gallinaceum* and perform better against *P. falciparum* because of the rigidity of the peptide bridge and possibe increased resistance to degradation under this kind of restriction.

**Figure 33:**
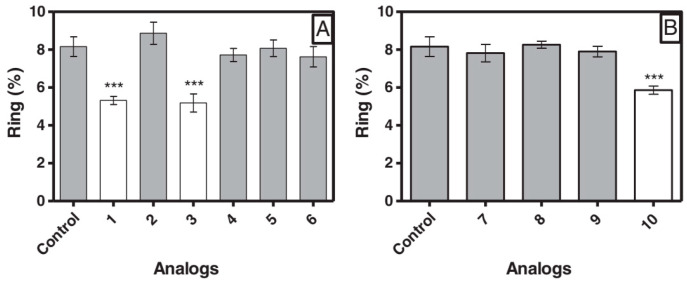
Effect of synthetic peptides in erythrocyte invasion by *Plasmodium falciparum*. (**A**) Analog 1–6; (**B**) Analog 7–10. *** Statistically significant compared with control value *p* < 0.05 Analog 1 = **416**; 2 = **417**; 3 = **418**; 4 = **419**; 5 = **420**; 6 = **421**; 7 = **422**; 8 = **423**; 9 = **424**; 10 = **425**.

Analog **416** and **418** presented significant antiplasmodial activity, however analog **425**, a disulfide bridge analog, was the most active peptide against *P. gallinaceum* (Figure 33). It was also active against *P. falciparum* because it has the same restriction size and implications when considering hydrophobic and hydrophilic interactions between side chains. All analogs frequently adopt a β-turn conformation, which is consistent with that of the analog that is believed to be most active against *P. gallinaceum* [32].

**Figure 34:**
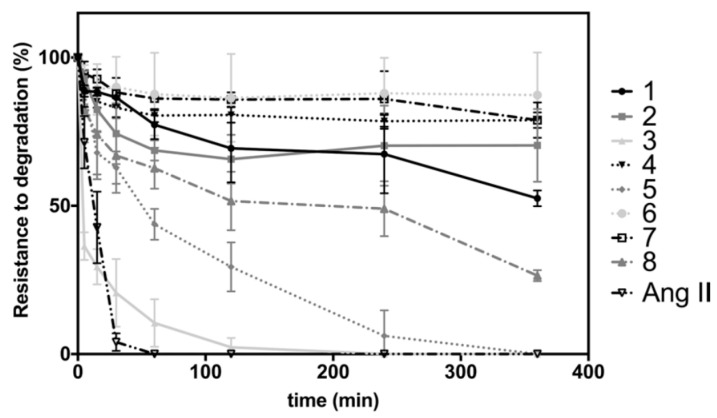
Stability of Ang II derivatives in human serum. Analog 1 = **426**; 2 = **427**; 3 = **428**; 4 = **429**; 5 = **430**; 6 = **431**; 7 = **432**; 8 = **433**.

Angiotensin II hormones were also synthesized to investigate erythrocytic cycle invasion [33]. Peptides **426** and **427** exhibited >80% activity on *P. gallinaceum* and >40% activity on *P. falciparum*. The hemolytic effect, contractile response, and stability in human serum were determined (Figure 34), demonstrating that the peptides did not present hemolytic effects as they were inactive when compared to positive controls and were resistant to degradation in human serum after a prolonged exposure (6 h), especially peptides **426** and **427**, which displayed excellent stability.

**Figure 35:**
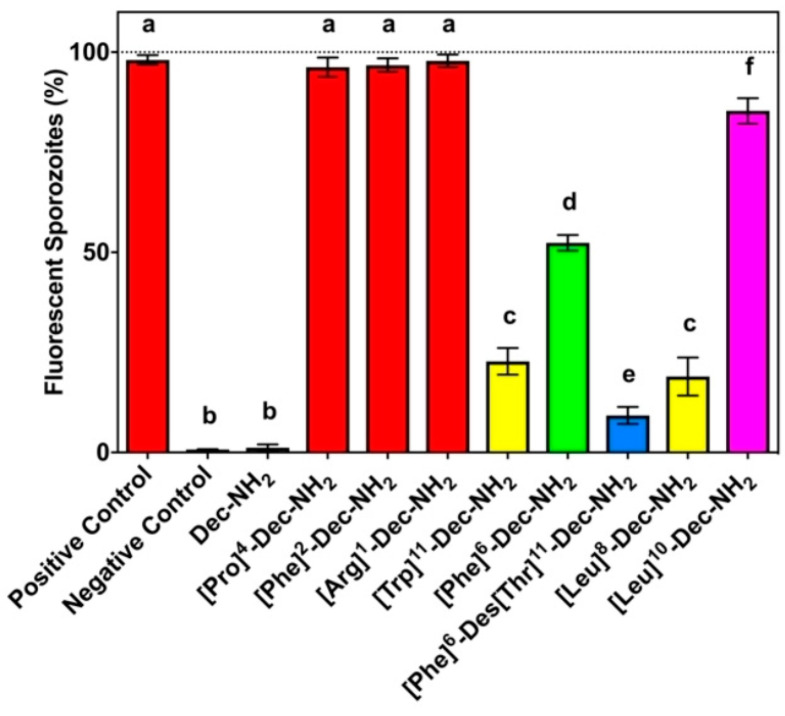
Effects of Dec-NH_2_ and analogs on membrane permeability. Positive control group (+): digitonin/PBS; negative control group (–): PBS. Peptide concentration = 60 mmol L^−1^. Letters indicate those results that are not significantly different from each other at *p* < 0.05 by Turkey’s test.

The study of synthesized Dec-NH_2_ and eight analog peptides reported that certain amino acid substitutions significantly improved the peptide’s antiplasmodial activity, providing new sequence principles for creating potent anti-malaria drugs, such as replacing the original sequence by Arg, Phe, and Trp [34]. The most active peptides examined were [Arg]^1^-Dec-NH_2_ (**438**), [Pro]^4^-Dec-NH_2_ (**437**), and [Phe]^2^-Dec-NH_2_ (**439**); all compounds substitutions improved the effectiveness, with the gihest antiplasmodial activities achieved with mutations to the N-terminus of Dec-NH_2_. A higher antiplasmodial activity was achieved by the introduction of a positive charge, increased hydrophobicity, and introduction of a restrictor residue (Figure 35).

## 4. Conclusions

This review on antimalarial peptides discussed the natural sources, chemical access and antimalarial properties of the peptides, showing that nature (higher plant, microbes, venom) is a source of these antimalarial peptides. Alternatively, they can be chemically synthesized using a variety of techniques, such as solution phase, solid phase and a combination of solid and solution phase synthesis. The selection of coupling reagent, cyclization site, and protection groups are important factors to consider for the successful synthesis. Most isolated and synthesized peptides are active against *P. falciparum* with some exhibiting inhibition of *P. gallinaceum* and *P. berghei*. Moreover, several peptides were also cytotoxic against MRC5-cells, HEK2931, and HepG2. The information presented in this review may be useful in the design and synthesis of new potent antimalarial agents.

## Figures and Tables

**Table 1:** Isolated antimalarial peptides.

Compound		Source	Reference
**1**	Ribifolin	*Jatropha ribifolia*	[3]
**2**	Hymenocardine	*Hymenocardia acida*	[4]
**3**	Hymenocardinol	*Hymenocardia acida*	
**4**	Hymenocardine *N*-oxide	*Hymenocardia acida*	
**5**	Hymenocardine-H	*Hymenocardia acida*	
**6**	Nummularine-R	*Ziziphus oxyphylla*	[5]
**7**	O-desmethylnummularine-R	*Ziziphus oxyphylla*	
**8**	O-desmethylnummularine-R *N*-oxide	*Ziziphus oxyphylla*	
**9**	Hemsine-A	*Ziziphus oxyphylla*	
**10**	Hemsine-A *N*-oxide	*Ziziphus oxyphylla*	
**11**	Ramosine-A	*Ziziphus oxyphylla*	
**12**	Oxyphylline-C	*Ziziphus oxyphylla*	
**13**	Oxyphylline-E	*Ziziphus oxyphylla*	
**14**	Oxyphylline-F	*Ziziphus oxyphylla*	
**15**	Crotamine	rattlesnake venom	[6]
**16**	Octaminomycins A	a microbial metabolite fraction library of *Streptomyces* sp. RK85-270	[7]
**17**	Octaminomycins B	a microbial metabolite fraction library of *Streptomyces* sp. RK85-270	
**18**	Kakeromamide B	Fijian marine cyanobacterium *Moorea producens*	[8]
**19**	Pipecolisporin	*Nigrospora oryzae*	[9]
**20**	Kosidachins A	*Pochonia binensis*	[10]
**21**	Kosidachins B	*Pochonia binensis*	
**22**	Georatusin	*Geomyces auratus*	[11]
**23**	Friomaramide B	*Inflatella coelosphaeroide*	[12]
**24**	Shagamide A	*Inflatella coelosphaeroide*	
**25**	Shagamide B	*Inflatella coelosphaeroide*	
**26**	Shagamide C	*Inflatella coelosphaeroide*	
**27**	Shagamide D	*Inflatella coelosphaeroide*	
**28**	Shagamide E	*Inflatella coelosphaeroide*	
**29**	Shagamide F	*Inflatella coelosphaeroide*	
**30**	NpCI	*Nerita versicolor*	[13]

**Table 2:** Synthesis of macrocyles by method A.

Peptide Precursor	Product	Time (Days)	Macrocycliz Yield ^c^/Purity ^d^ (%)	Overall Yield ^e^ (%)
H-L-Cys(Trt)-Gly-L-Thr(^t^Bu)-Gly-L-Cys(Trt)-Gly-OH	**140**	3	34 ^a^/98	32
H-L-Ser(^t^Bu)-L-Phe-L-Cys(Trt)-L-Met-L-Cys(Trt)-Gly-OH	**141**	3	38 ^a^/85	31
H-L-Val-Ser(^t^Bu)-L-Phe-L-Cys(Trt)-L-Ile-L-Cys(Trt)-OH	**142**	3	43 ^a^/82	42
H-L-Cys(Trt)-L-Arg(Pbf)-L-Cys(Trt)-L-Phe-L-Ser(^t^Bu)-Gly-OH	**143**	3	27 ^a^/80	22
H-L-Cys(Trt)-L-Ile-L-Cys(Trt)-L-Phe-L-Arg(Pbf)-Gly-OH	**144**	3	26 ^a^/93	23
H-L-Cys(Trt)-L-Cys(Trt)-L-Ile-L-Phe-L-Glu(γ-^t^Bu)-Gly-OH	**145**	1	46 ^a^/89–59 ^b^/85	43 ^a^/55 ^b^
H-L-Cys(Trt)-L-Cys(Trt)-L-Ile-L-Phe-L-Glu(^t^Bu)-Gly-OH	**146**	2	59 ^a^/83	51
H-L-Ser(^t^Bu)-L-Ile-L-Ser(^t^Bu)-L-Phe-L-Glu(^t^Bu)-Gly-OH	**147**	3	34 ^a^/93	32
H-L-Met-L-Phe-L-Phe-L-Glu(^t^Bu)-L-Met-Gly-OH	**148**	2	80 ^a^/93	63
H-L-Cys(Trt)-L-Cys(Trt)-L-Phe-L-Ser(^t^Bu)-Gly-OH	**149**	1	54 ^a^/94	51
H-L-Cys(Trt)-L-Cys(Trt)-L-Ser(^t^Bu)-Gly-OH	**150**	1	69 ^a^/96	58

^a^ HBTU was used as a coupling reagent for macrocyclization. ^b^ HATU was used as a coupling reagent for macrocyclization. ^c^ For the purified final compounds. ^d^ Determined by HPLC analysis. ^e^ Yields for the linear peptides were based on the determination of the resin loading.

**Table 3:** Synthesis of macrocycles by method B and DIC/Cl-HOBt.

Peptide Precursor	Product	Crude Yield ^a^/Purity ^b^ (%)	Isolated Macrocycle Yield ^a^/Purity ^b^ (%)	Time (h)
H-Gly-L-Cys(Trt)-L-Ile-L-Cys(Trt)-L-Phe-L-Glu-OH	**151**	68/81	51/95	16
H-Gly-L-Cys(Trt)-L-Cys(Trt)-L-Ile-L-Phe-L-Glu-OH	**152**	98/72	63/95	16
H-Gly-L-Ser(^t^Bu)-L-Ile-L-Ser(^t^Bu)-L-Phe-L-Glu-OH	**153**	63/78	46/93	16
H-L-Met-Gly-L-Met-L-Phe-L-Phe-L-Glu-OH	**154**	99/83	80/95	16

^a^ Yield based on the determination of resin loading. ^b^ Determined by HPLC analysis.

**Table 4:** Comparison of methods A and B.

Macrocyle Methodology A	Yield/Purity	Macrocycle Methodology B	Yield/Purity
**155**	59 ^a^/85	78	51/95
**156**	59/83	79	63/95
**157**	34/93	80	46/93
**158**	80/93	81	80/95

^a^ HATU was used as coupling reagent for macrocyclization.

**Table 5:** List of synthesized depsipeptide and peptide analogs of hirsutellide A.

cpd	I	II	III	X	R
**177**	*allo*-Ile	_D_-Phe	Gly	–O–	–CH_3_
**178**	Leu	_D_-Phe	Gly	–O–	–CH_3_
**179**	Val	_D_-Phe	Gly	–O–	–CH_3_
**180**	Gly	_D_-Phe	Gly	–O–	–CH_3_
**181**	Ile	_D_-Phe	Gly	–O–	–H
**182**	Ile	_D_-Phe	Gly	–NH–	–CH_3_
**183**	Val	_D_-Phe	Gly	–NH–	–CH_3_
**184**	*allo*-Ile	_D_-Phe	Gly	–NH–	–CH_3_
**185**	*allo*-Ile	_D_-Phe (4-Cl)	Gly	–NH–	–CH_3_
**186**	*allo*-Ile	_D_-Phe (4-OCH_3_)	Gly	–NH–	–CH_3_
**187**	*allo*-Ile	_D_-Phe	Gly	–NH–	–CH_3_
**188**	*allo*-Ile	_D_-Phe	Pro	–NH–	–CH_3_

**Table 6:** Yield of falcitidin acylated analogs **296**–**303**.

Entry	R^1^C(O)	R^2^	Yield (%)
**296**	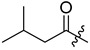	Tr	79 (from **295**)
**297**	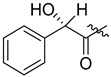	H	80 (from **296**)
**298**		Tr	67 (from **295**)
**299**		H	20 (from **298**)
**300**	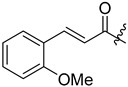	H	37 (from **295**)
**301**	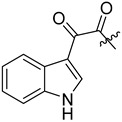	H	33 (from **295**)
**302**		Tr	89 (from **295**)
**303**	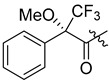	H	70 (from **302**)

**Table 7:** Yields of gallinamide A analogs **313**–**318**.

Analogue	R^2^	R^1^	Yield (Based on Original Resin Loading)
**313**	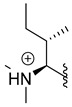	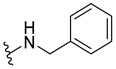	40% (5:1 *d.r.*)
**314**		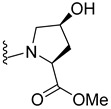	33% (1:1 *d.r.*)
**315**	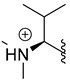	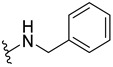	65% (7:1 *d.r.*)
**316**		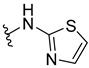	62% (1:1 *d.r.*)
**317**		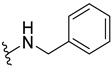	78% (6:1 *d.r.*)
**318**	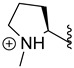		75% (5:1 *d.r.*)

**Table 8:** List of tripeptides synthesized to generate compounds **371**–**387**.

cpd	R^4^	R^3^	R^2a^	R^2b^	Yield (%)
**358**	Me_2_NCH(*^i^*Pr)–	–CH_2_CH(CH_3_)_2_	–CH_2_CH(CH_3_)_2_	–H	99
**359**	Me_2_NCH(*^i^*Pr)–	–CH_2_-cyclohexyl	–CH_2_CH(CH_3_)_2_	–H	98
**360**	4-(*N*-Me)-piperidine–	–CH_2_-cyclohexyl	–CH_2_CH(CH_3_)_2_	–H	94
**361**	4-(*N*-Me)-piperidine–	–CH_2_CH(CH_3_)_2_	–CH_2_CH(CH_3_)_2_	–H	99
**362**	Me_2_NCH(*^i^*Pr)–	–CH_2_Ph	–CH_2_CH(CH_3_)_2_	–H	91
**363**	Me_2_NCH(*^i^*Pr)–	–CH_2_CH_2_Ph	–CH_2_CH(CH_3_)_2_	–H	98
**364**	Me_2_NCH(*^i^*Pr)–	–CH_2_CH(CH_3_)_2_	–CH_2_CH_2_Ph	–H	94
**365**	Me_2_NCH(*^i^*Pr)–	–CH_2_-cyclohexyl	–CH_2_CH_2_Ph	–H	90
**366**	Me_2_NCH(*^i^*Pr)–	–CH_2_-cyclohexyl	–CH_2_-cyclohexyl	–H	90
**367**	Me_2_NCH(*^i^*Pr)–	–CH_2_CH(CH_3_)_2_	–CH_2_-cyclohexyl	–H	95
**368**	4-(*N*-Me)-piperidine–	–CH_2_-cyclohexyl	–CH_2_-cyclohexyl	–H	81
**369**	4-(*N*-Me)-piperidine–	–CH_2_CH(CH_3_)_2_	–CH_2_-cyclohexyl	–H	98
**370**	Me_2_NCH(*^i^*Pr)–	–CH_2_CH(CH_3_)_2_	–CH_3_	–CH_3_	93

**Table 9:** Overview of the yields of synthetic gallinamide A analogs [28].

cpd	R^4^	R^3^	R^2a^	R^2b^	R^1a^	R^1b^	Yield (%)
**352**	Me_2_NCH(*^i^*Pr)–	–CH_2_CH(CH_3_)_2_	–CH_2_CH(CH_3_)_2_	–H	–CH_3_	–H	91
**371**	Me_2_NCH(*^i^*Pr)–	–CH_2_-cyclohexyl	–CH_2_CH(CH_3_)_2_	–H	–CH_3_	–H	72
**372**	4-(*N*-Me)-piperidine–	–CH_2_-cyclohexyl	–CH_2_CH(CH_3_)_2_	–H	–CH_3_	–H	86
**373**	4-(*N*-Me)-piperidine–	–CH_2_CH(CH_3_)_2_	–CH_2_CH(CH_3_)_2_	–H	–CH_3_	–H	78
**374**	Me_2_NCH(*^i^*Pr)–	–CH_2_Ph	–CH_2_CH(CH_3_)_2_	–H	–CH_3_	–H	92
**375**	Me_2_NCH(*^i^*Pr)–	–CH_2_CH_2_Ph	–CH_2_CH(CH_3_)_2_	–H	–CH_3_	–H	83
**376**	Me_2_NCH(*^i^*Pr)–	–CH_2_CH(CH_3_)_2_	–CH_2_CH_2_Ph	–H	–CH_3_	–H	90
**377**	Me_2_NCH(*^i^*Pr)–	–CH_2_-cyclohexyl	–CH_2_CH_2_Ph	–H	–CH_3_	–H	87
**378**	Me_2_NCH(*^i^*Pr)–	–CH_2_-cyclohexyl	–CH_2_-cyclohexyl	–H	–CH_3_	–H	93
**379**	Me_2_NCH(*^i^*Pr)–	–CH_2_CH(CH_3_)_2_	–CH_2_-cyclohexyl	–H	–CH_3_	–H	71
**380**	4-(*N*-Me)piperidine–	–CH_2_-cyclohexyl	–CH_2_-cyclohexyl	–H	–CH_3_	–H	68
**381**	4-(*N*-Me)piperidine–	–CH_2_CH(CH_3_)_2_	–CH_2_-cyclohexyl	–H	–CH_3_	–H	95
**382**	Me_2_NCH(*^i^*Pr)–	–CH_2_CH(CH_3_)_2_	–CH_3_	–CH_3_	–CH_3_	–H	81
**383**	Me_2_NCH(*^i^*Pr)–	–CH_2_CH(CH_3_)_2_	–CH_2_CH(CH_3_)_2_	–H	–CH_3_	–CH_3_	51 ^a^
**384**	Me_2_NCH(*^i^*Pr)–	–CH_2_CH(CH_3_)_2_	–CH_2_CH(CH_3_)_2_	–H	–cyclohexyl	–H	69
**385**	Me_2_NCH(*^i^*Pr)–	–CH_2_-cyclohexyl	–CH_2_CH(CH_3_)_2_	–H	–cyclohexyl	–H	65
**386**	Me_2_NCH(*^i^*Pr)–	–CH_2_CH(CH_3_)_2_	–CH_2_CH(CH_3_)_2_	–H	–CH_2_-cyclohexyl	–H	88
**387**	Me_2_NCH(*^i^*Pr)–	–CH_2_CH(CH_3_)_2_	–CH_2_CH(CH_3_)_2_	–H	–CH_2_CH_2_Ph	–H	83

^a^ Combined yield of diastereomers.

**Table 10:** Amino acid sequences of the peptides from PfSERA5.

Peptides	Amino Acid Sequence	Position	Mol. Mass (Da)
SE5 P1 (**388**)	Ac-TTKKESKIYDYYL-NH_2_	**830**–**842** aa	1821
SE5 P2 (**389**)	Ac-KASPEFYHNLYFKNF-NH_2_	**843**–**857** aa	1946
SE5 P3 (**390**)	Ac-NVGKKNLFSEKEDN-NH_2_	**858**–**871** aa	1663
SE5 P4 (**391**)	Ac-KKYVKLPSNGTTGEQ-NH_2_	**176**–**190** aa	1691
SE5 P5 (**392**)	Ac-GSSTGTVRGDTEPISDS-NH_2_	**191**–**207** aa	1707
SE5 P6 (**393**)	Ac-SESLPANGPDSPTVK-NH_2_	**233**–**247** aa	1540
SE5 P7 (**394**)	Ac-FKEIKAETEDDDEDDY-NH_2_	**384**–**399** aa	2003
SE5 P8 (**395**)	Ac-YIIFGQDTAGSGQSGK-NH_2_	**881**–**896** aa	1670
SE5 P9 (**396**)	Ac-TALESAGTSNEVSERV-NH_2_	**900**–**915** aa	1620

Note: aa= amino acid

**Table 11:** Purity of peptides determined by LC/MS [30].

Entry	Name	Sequence	HPLC Purity ^a^ (%)	Calcd Mass ^b^ (g mol^−1^)	Obsd Mass ^b^ (g mol^−1^)
**397**	[Ile^6^,His^5^]-All	DRVYHIPF	98	1045.5	1046
**398**	des-Ile^5^, His^6^-All	DRVYPF	98	795.4	796
**399**	des-Asp^1^-Val^3^,Ile^5^,His^6^-All	RYPF	99	581.3	582
**400**	[Ile^4^,His^3^]-des-Asp^1^,Val^3^-All	RYHIPF	98	831.4	833
**401**	des-Asp^1^,Arg^2^,Val^3^,Ile^5^-All	YHPF	99	562.2	563
**402**	des-Asp^1^,Arg^2^,Tyr^4^,His^6^-All	VIPF	99	474.3	475
**403**	[2-Na^1^]-des-Asp^1^-Arg^2^,Tyr^4^,His^6^-All	(2-NaI)-IPF	97	572.3	574
**404**	[2-Na^2^]-des-Asp^1^-Arg^2^,Tyr^4^,His^6^-All	V-(2-NaI)-PF	99	558.3	559
**405**	[2-Na^3^]-des-Asp^1^-Arg^2^,Tyr^4^,His^6^-All	VI-(2-NaI)-F	97	574.3	575
**406**	[2-Na^4^]-des-Asp^1^-Arg^2^,Tyr^4^,His^6^-All	VIP-(2-NaI)	98	523.3	524

^a^ HPLC profiles were obtained under the following conditions: Column Supelcosil C18 (4.6 × 150 mm), 60 Å, 5 μm; Solvent System: A (0.1% TFA/H_2_O) and B (0.1% TFA in 60% ACN/H_2_O); Gradient: 5–95% B in 30 min; Flow: 1.0 mL/min; λ = 220 nm; Injection Volume: 50 μL and sample concentration: 1.0 mg/mL. ^b^ The masses were determined by LC/ESI–MS using a Micromass instrument (model ZMD) coupled to a Waters Alliance (model 2690) system. Mass measurements were performed in a positive mode with the following parameters: mass range between 300 and 2000 *m*/*z*; nitrogen gas flow: 4.1 L/h; capillary: 2.3 kV; cone voltage: 32 V; extractor: 8 V; source heater: 100 °C; solvent heater: 400 °C; ion energy: 1.0 V and multiplier: 800 V.

**Table 12:** Sequences of compounds **416**–**425** [32].

Entry	Sequence	HPLC Purity ^a^	Calculated Mass	Observed Mass ^b^
**416**	Glu-Asp-Arg-Orn-Val-Tyr-Ile-His-Pro-Phe	99%	1272.4	1271.4
**417**	Glu-Asp-Arg-Lys-Val-Tyr-Ile-His-Pro-Phe	99%	1284.4	1286.1
**418**	Asp-Glu-Arg-Val-Orn-Tyr-Ile-His-Pro-Phe	99%	1272.4	1272.4
**419**	Asp-Glu-Arg-Val-Lys-Tyr-Ile-His-Pro-Phe	99%	1284.4	1286.4
**420**	_D_Glu-Asp-Arg-Val-Orn-Tyr-Ile-His-Pro-Phe	98%	1272.4	1272.4
**421**	Glu-Asp-Arg-Val-_D_Orn-Tyr-Ile-His-Pro-Phe	98%	1272.4	1272.4
**422**	Asp-Arg-Cys-Val-Cys-Tyr-Ile-His-Pro-Phe	99%	1249.4	1250.4
**423**	Cys-Asp-Arg-Val-Cys-Tyr-Ile-His-Pro-Phe	98%	1249.4	1250.4
**424**	Asp-Cys-Arg-Cys-Val-Tyr-Ile-His-Pro-Phe	99%	1249.4	1250.4
**425**	Cys-Asp-Arg-Cys-Val-Tyr-Ile-His-Pro-Phe	99%	1249.4	1249.4

^a^ HPLC profiles were obtained under the following conditions: Column Supelcosil C18 (4.6 × 150 mm), 60 Å, 5 μm; Solvent System: A (0.1% TFA/H_2_O) and B (0.1% TFA in 60% ACN/H_2_O); gradient: 5–95% B in 30 min; flow: 1.0 mL min1; λ = 220 nm; injection volume: 50 μL; and sample concentration: 1.0 mg mL*^−^*^1^. ^b^ The masses were determined by LC/ESI-MS using a Micromass instrument (model ZMD) coupled to a Waters Alliance (model 2690) system. Mass measurements were performed in a positive mode with the following parameters: mass range between 500 and 2000 *m*/*z*; nitrogen gas flow: 4.1 L h*^−^*^1^; capillary: 2.3 kV; cone voltage: 32 V; extractor: 8 V; source heater: 100 °C; solvent heater: 400 °C; ion energy: 1.0 V, and multiplier: 800 V.

**Table 13:** Design of angiotensin II-derived synthetic peptides [33].

Entry	Sequence	HPLC Purity ^a^ (%)	Calculed Mass ^b^ (g mol^−1^)	Observed Mass ^b^ (g mol^−1^)	Antiplasmodial Activity vs. *P. gallinaceum* (% Fluorescent Sporozoltes)	Antiplasmodial Activity vs. *P. falciparum* (New Ring Formation%)	Design Rationale
Ang II	DRVYIHPF				88	47	WT peptide
**426**	CDRVYIHPFC	98	1249.6	1250	92	42	Contrained version of Ang II
**427**	CDRVYHIPFC	98	1249.6	1250	91	42	Residues H and I were reversed, which is expected to increase antiplasmodial activity based on previously described linear variant.
**428**	CDRVCYHIPF	98	1249.6	1250	82	11	Designed based on most potent antiplasmodial peptide (CDRVCYIHPF) obtained previously, with an additional 1H reverse modification.
**429**	CDRVYPFC	97	999.4	1000	95	9	Hydrophobic patch composed of H and I was deleted to asses its influence on functions
**430**	CRYHIPFC	98	1035.5	103.6	98	0	D and V were deleted to optimize interactions between R and Y within the same plane. I and H were reversed to evaluate the effect of new pi-stack interactions in the activity.
**431**	CRYPFC	99	765.3	786	4	29	Most important residues for function within the Ang II sequence were kept to build a minimal peptide with antiplasmodial activity.
**432**	CYHPFC	97	766.3	767	6	17	Only aromatic residues were kept, inspired by the linear form (YHPF), which was one of our lead peptides previously described.
**433**	CVIPFC	98	675.3	676	7	3.5	Aliphatic and hydrophobic residue F were kept based on previous lead linear peptide (VIPF)

Purity of peptides determined by LC/MS is shown, along with their respective antiplasmodial activity and design rationale. ^a^ HPLC profiles were obtained under the following conditions: Column Supelcosil C18 (4.6 × 150 mm), 60 Å, 5 μm; Solvent System: A (0.1% TFA/H_2_O) and B (0.1% TFA in 60% ACN/H_2_O); Gradient: 5–95% B in 30 min; Flow: 1.0 mL/min; λ = 220 nm; Injection Volume: 50 μL and sample concentration: 1.0 mg/mL. ^b^ The masses were determined by LC/ESI–MS using a Micromass instrument (model ZMD) coupled to a Waters Alliance (model 2690) system. Mass measurements were performed in a positive mode with the following parameters: mass range between 300 and 2000 *m*/*z*; nitrogen gas flow: 4.1 L/h; capillary: 2.3 kV; cone voltage: 32 V; extractor: 8 V; source heater: 100 °C; solvent heater: 400 °C; ion energy: 1.0 V and multiplier: 800 V.

**Table 14:** Characterization and theoretical physicochemical properties of decoralin and synthetic analogs.

Peptide	Sequence	H	µ_H_	q	Purity ^a^	Calculated Mass (Da)	Observed Mass ^b^ (Da)
Dec-NH_2_ (**434**)	SLLSLIRKLIT-NH_2_	0.780	0.652	+3	99%	1254.8	1256
[Leu]^8^-Dec-NH_2_ (**435**)	SLLSLIRLLIT-NH_2_	1.025	0.479	+2	98%	1239.8	1241
[Leu]^10^-Dec-NH_2_ (**436**)	SLLSLIRKLLT-NH_2_	0.771	0.647	+3	99%	1254.8	1256
[Pro]^4^-Dec-NH_2_ (**437**)	SLLPLIRKLIRT-NH_2_	0.849	0.583	+3	99%	1265.8	1267
[Arg]^1^-Dec-NH_2_ (**438**)	RLLSLIRKLIT- NH_2_	0.692	0.704	+4	99%	1339.9	1340
[Phe]2-Dec-NH_2_ (**439**)	SFLSLIRKLIT- NH_2_	0.788	0.659	+3	99%	1288.8	1290
[Phe]^6^-Dec-NH_2_ (**440**)	SLLSLFRKLIT- NH_2_	0.779	0.651	+3	99%	1288.8	1290
[Phe]^6^-Dec[Thr]^11^-Dec-NH_2_ (**441**)	SLLSLFRKLI- NH_2_	0.831	0.392	+3	98%	1188.8	1190

^a^ HPLC profiles were obtained under the following conditions: Column Phenomenex C_18_ (4,6 × 150 mm), 60 Å, 5 µm; Solvent System: A (0.1% TFA/H_2_O) and B (60% ACN/A), Gradient 5–95% B in 30 min; Flow 1.0 mL min^−1^; λ = 220 nm, injection Vol.: 100 µL and Sample Concentration: 1.0 mg mL^−1^. ^b^ The molecular ions are reported as their average calculated and measured mass-to-charge ratios (*m*/*z*).

**Table 15:** Orbitides isolated from Jatropha species and their potential antiplasmodial activities.

Species	Compounds	Sequences	Antiplasmodial Activity (IC_50_ (µM))	References
*Jatropha ribofilia*	Ribifolin (1)	cyclo ILGSIILG	42	[3]
*Jatropha curcas*	Curcacyline B	cyclo ILGSPILLG	10,000	[3,36]
*Jatropha chevalieri*	Chevalierin A	cyclo IMGIPILA	9	[3,37]
*Jatropha mahafalensis*	Mahafacyclin A	cyclo VFGTILG	16	[3,38]
*Jatropha mahafalensis*	Mahafacyclin B	cyclo FFGTFFG	2	[3,39]
*Jatropha pohliana*	Pohlianin A	cyclo VLLYPLG	57	[3,40]
*Jatropha pohliana*	Pohlianin B	cyclo LLLYPLG	25	[3,40]
*Jatropha pohliana*	Pohlianin C	cyclo FGGTIIFG	16	[3,40]

**Table 16:** Antiplasmodial activity against against *P. falciparum* strain KI and cytotoxicity against MRC5-Cells (IC_50_ μM) of compounds **2** (Hymenocardine), **3** (Hymenocardinol), **4** (Hymenocardine N-Oxide), and **5** (Hymenocardine-H) from the root bark of Hymenocardia acida.

Compound	*P. falciparum* KI	MRC-5
**2**	16.4 ± 7	51.1 ± 17
**3**	17.5 ± 9	>64.0
**4**	12.2 ± 7	>64.0
**5**	27.9 ± 17	>64.0
chloroquine	0.2 ± 0.1	

**Table 17:** Antiplasmodial activity against against *P. falciparum* strain K1 and cytotoxicity against MRC5-Cells (IC_50_ μM) for compounds **6**, **7**, **9**, **11**, and **14** from the roots of Ziziphus oxyphylla.

Compound	*P. falciparum* KI	MRC-5
**6**	3.2 ± 3	30.6 ± 4
**7**	7.1 ± 2	>64.0
**9**	13.6 ± 9	>64.0
**11**	>32.0	>64.0
**14**	7.4 ± 3	31.2 ± 1
chloroquine	0.3 ± 0.2	

**Table 18:** Antimalarial activities for octaminomycins A (**16**) and B (**17**) with chloroquine as positive control against chloroquine-sensitive (3D7) and resistant (Dd2 and K1) strains of *P. falciparum* (IC_50_ in μM).

Compound	*P. falciparum* 3D7	*P. falciparum* Dd2	*P. falciparum* KI
**16**	1.5	1.6	1.3
**17**	1.5	1.1	0.83
chloroquine	0.041	0.52	0.46

**Table 19:** Antimalarial activity and cytotoxicity of kakeromamide B (**18**).

Compound	Blood-Stage *P. falciparum*	Liver-Stage *P. berghei*	HEK2931 Cytotoxicity	HepG2 Cytotoxicity
**18**	8.9	11, >12	>23	>23
Atovaquone (+control)	0.0061	<0.00028, 0.0017, 0.0037	>2.0	not tested

**Table 20:** In vitro antiplasmodial activity, cytotoxicity, and selectivity index of koshidacins A (**20**) and B (**21**) and artemisinin.

Compound	IC_50_ (μM)				
	Antiplasmodial Activity		Cytotoxicity	Selectivity Index	
	*pf* KI ^a^	*pf* FCR3 ^b^	MRC-5	MRC-5/*pf* KI	MRC-5/*pf* FCR3
**20**	12.5 ± 1	17.1 ± 1.0	6.8 ± 2	0.5	0.4
**21**	0.83 ± 0.04	0.89 ± 0.04	14.7 ± 4	18.4	16.3
artemisinin ^c^	0.022 ± 0.001	0.026 ± 0.003	>25	>1136	>961

^a^ Cloroquine-resistant strain. ^b^ Chloroquine-sensitive strain. ^c^ Drug commonly used to treat malaria.

**Table 21:** In vivo antiplasmodial activity in the Peters’ 4-day suppressive test of koshidacins A (**20**) and B (**21**) and artesunate (30 mg/kg/day × 4 days, i.p.) in a mouse model.

Compound	Inhibition (%)
**20**	18.3 ± 6
**21**	41.0 ± 2
Artesunate ^a^	99.7 ± 0.10

^a^ Drug commonly used to treat malaria.

**Table 22:** IC_50_ (µM) of compounds **23**–**29** against blood-stage *P. falciparum* strains.

Compound	NF54	Dd2	3D7
**23**	3.93	3.30	8.65
**24**	2.61	1.72	2.10
**25**	>10	4.37	>10
**26**	3.73	1.07	2.52
**27**	3.05	2.02	>10
**28**	>10	>10	>10
**29**	>10	>10	>10
CQ	0.03	1.28	>10

CQ = chloroquine.

**Table 23:** The biological activity of the new cyclomarin derivatives against Pfalcp (chloroquine sensitive 3D7, multi-resistant Dd2 strains, and Mtb (Erdman wt strain)), as well as the overall synthesis processes.

Entry	Compound	R′	R″	R‴	*Pfalcp* IC_50_ [nm] 3D7	IC_50_ [nm] Dd2	*Mtb* MIC [µM] wt	Overall Synthetic Steps
ref *Mtb*	Isoniazid						0.9	
ref *Pfalcp*	CQ				3.4	233.9		
1	desoxycyclomarin C	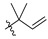	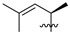	Me	39.8	NA	0.9	37
2	**96**	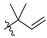	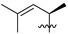	Me	9.0	12.9	0.5	34
3	**97**	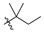	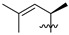	Me	4.4	6.5	1.7	31
4	**98**	*i*Pr	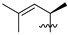	Me	47.8	76.0	0.4	36
5	**99**	Me	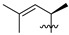	Me	13.4	17.8	0.9	31
6	**100**	H	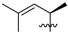	Me	28.1	27.5	1.5	31
7	**106**	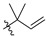	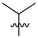	Me	34.4	71.8	21.2	32
8	**107**	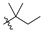	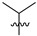	Me	57.6	200.2	1.7	33
9	**108**	*i*Pr	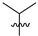	Me	355.7	300.3	0.4	34
10	**109**	Me	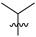	Me	230.3	256.7	1.6	29
11	**110**	H	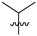	Me	314.2	421.5	0.5	29
12	**119**	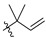	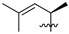	H	47.9	36.7	2.3	32
13	**120**	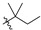	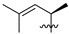	H	452.0	346.5	>32	33
14	**121**	*i*Pr	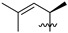	H	177.5	287.8	1.8	34
15	**122**	Me	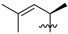	H	362.4	318.4	3.4	32
16	**123**	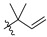	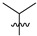	H	303.5	260.8	2.6	30
17	**124**	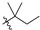	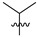	H	428.5	305.1	3.2	31
18	**125**	H	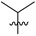	H	287.4	196.7	4.3	36

**Table 24:** Antiplasmodium activities of macrocycles.

Compound	EC_50_ *P. falciparum* K1 (µM)	Compound	EC_50_ *P. falciparum* K1 (µM)
**140**	0.028 ± 0.006	**148**	4.6 ± 1
**141**	0.68 ± 0.04	**149**	8.4 ± 1
**142**	6.50 ± 0.05	**150**	0.73 ± 0.06
**143**	16.8 ± 1	**151**	0.47 ± 0.04
**144**	43.1 ± 1	**152**	0.42 ± 0.05
**145**	0.42 ± 0.03	**153**	>50
**146**	>10	**154**	>50
**147**	3.8 ± 1		

Control: chloroquine, EC_50_ = 0.47 µM. Artemisinin: EC_50_ = 0.02 µM. Artesunate: EC_50_ = 0.003 µM.

**Table 25:** Parasite growth inhibition and selectivity index^a^.

Cyclopeptide	EC_50_/EC_50_ *P. falciparum* K1 (µM) ^b^	EC_50_ *P. falciparum* 3D7 (µM) ^c^	SI IC_50_^d^/IC_50_ P*f*K1	SI IC_50_^e^/IC_50_ P*f*3D7
**156**	8.0 ± 0.5/39 ± 3	3.8 ± 0.1	>1250	>6.6 × 10^4^
**157**	0.008 ± 0.004/1.0 ± 0.5	0.25 ± 0.02	>1.3 × 10^7^	>1.0 × 10^6^
**158**	0.040 ± 0.006/1.0 ± 0.3	1.0 ± 0.3	>2.5 × 10^6^	>2.5 × 10^5^
**159**	0.13 ± 0.04/4.0 ± 0.6	1.4 ± 0.6	>7.7 × 10^5^	>1.8 × 10^5^
**160**	9.0 ± 0.5/59 ± 3	1.8 ± 0.1	>1.1 × 10^4^	> 1.4 × 10^5^
**161**	150 ± 5/900 ± 9	nt	nt	nt
**163**	nt	5400 ± 100	nt	>46
**164**	nt	210 ± 10	nt	>1.2 × 10^3^
**165**	0.20 ± 0.04/4.0 ± 0.6	12 ± 1	5 × 10^5^	>2 × 10^4^

^a^ Values represent the mean ± SD (*n* = 2). ^b^ Control: chloroquine, EC_50_ = 0.47 µM; Artemisinin, EC_50_ = 20 µM; Artesunate, EC_50_ = 3 µM. ^c^ Control: Pyrimethamine, EC_50_ = 35 µM; Artesunate, EC_50_ = 5 µM. ^d^ Cytotoxicity against murine macrophages J774. ^e^ Cytotoxicity against HepG2 cells; nt: not tested.

**Table 26:** Biological activities and ADME profile of compound **177**.

Bioactivity/ADME Profile Compound 177	Values
antimicrobial activities	
MIC (Mtb H73Rv)	>80 µM
MIC (*M. vaccae*)	18.8 µM
IC_50_ (*P. falciparum* 3D7)	2.3 µM
cytotoxicity	
IC_50_ (Hep2G cells)	>100 µM
membrane permeability ^a^	280 nm/s
% human serum albumin binding	88%
Intrinsic microsomal clearance (Cl_int_)	
Human	46.53 mL/(min g) tissue
Mouse	38.3 mL/(min g) tissue
Plasma stability (% remain after 2 h incubation) ^b^	
Human plasma	103%
Mouse plasma	18%

^a^ Passive artificial membrane permeability. ^b^ Estimated by quantifying the amount of a compound remaining after incubation with human and mouse plasma for 2 h at 37 °C.

**Table 27:** Optimization of the N-Terminal Cap and Linker.

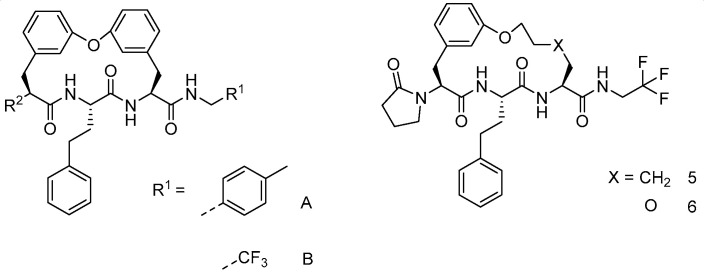												
			EC_50_ (µM)	β5 IC_50_ (µM)			Permeability		Liver Microsomal Stability	Kinetic Solubility	Cytotoxicity HepG2	Lipopholicity
ID	R^1^	R^2^	3D7	Pf20S	c20S	i20S	PAMPA	MDR	m/hLM	pH 6.8	Viability %	cLogP
**cyclic peptide 201**	A	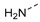	0.078	0.13	>100	>100	Un-detected	>2.9/<1	90.6 179.4	<1.6	71	6.3
**202**	A	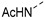	0.002	0.017	>100	>100	22	>34/1	171.3 N.D.	-	93	6.2
**203**	B	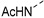	0.003	0.014	4.0	>100	151	>0.71/<4	269.7 11.2	<1.4	93	4.7
**204**	B	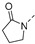	0.0002	0.003	26.3	>100	214	31/5	>768 644.3	3.8	77	5.3
**214**	-	-	0.002	0.019	2.4	>100	187	53/2	566.6 349.8	76	97	4.2
**220**	-	-	0.019	0.080	6.4	>100	120	89/2	397.4 275.7	>130	109	3.0

PAMPA unit: nm/s; MDR unit: A→B, nm/s; ratio (B→A/A→B); Metabolic stability Clint unit: μL/min/mg; Kinetic solubility (μg/mL); Cytotoxicity: % viability at 30 μM. “-”: not tested.

**Table 28:** Optimization of the P1 Group.

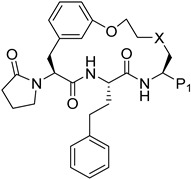											
		EC_50_ (µM)	β5 IC_50_ (µM)			Permeability		Metabolic Stability	Kinetic Solubility	Cytotoxicity HepG2	Lipopholicity
ID	P1	3D7	Pf20S	c20S	i20S	PAMPA	MDR	m/hLM	pH 6.8	Viability %	cLogP
**220**	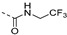	0.002	0.019	2.4	>100	187	53/2	566.6 349.8	76	97	4.2
**221**	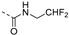	0.008	0.022	8.9	>100	72	>11/<5	288 187.7	35	101	4.0
**222**	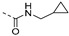	0.003	0.007	3.0	>100	103	68/2	477.6 340.6	49	101	4.4
**223**	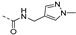	0.008	0.054	9.8	>100	11	>6/<1	261 187.7	>120	107	3.4
**224**	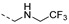	>2.77	23.8	>100	>100	-	-	-	-	-	4.8
**225**	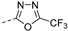	>2.77	9.0	>100	>100	160	11/17	>768 672.1	6.2	109	3.3
**226**	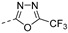	>2.77	64.0	>100	>100	82	>21/<1	59.6 28.6	2	103	3.7
**227**	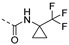	0.398	2.5	>100	>100	145	44/2	703.4 288	31	111	4.5
**228**	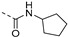	0.001	0.003	2.5	>100	151	57/3	657.8 596.5	100	96	4.9

PAMPA unit: nm/s; MDR unit: A→B, nm/s; ratio (B→A/A→B); Metabolic stability Clint unit: μL/min/mg; Kinetic solubility (μg/mL); Cytotoxicity: % viability at 30 μM. “-”: not tested.

**Table 29:** Optimization of the P3 Group.

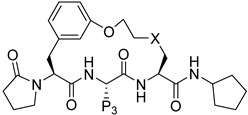											
		EC_50_ (µM)	β5 IC_50_ (µM)			Permeability		Metabolic Stability	Kinetic Solubility	Cytotoxicity HepG2	Lipopholicity
ID	P3	3D7	Pf20S	c20S	i20S	PAMPA	MDR	m/hLM	pH 6.8	Viability %	cLogP
**228**	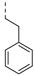	0.001	0.003	2.5	>100	151	57/3	657.8 596.5	100	96	4.9
**247**	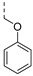	0.072	0.91	13.1	>100	208		529 405	20	104	4.5
**254**	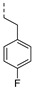	0.011	0.009	2.7	51.0	212	45/3	>768 618	17	100	5.0
**272**	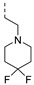	0.012	0.043	3.2	46.5	133	49/1	128 113	>130	102	2.8
TDI8304	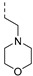	0.016	0.081	>100	>100	15	>2/<1	6 19	>130	103	2.4

PAMPA unit: nm/s; MDR unit: A→B, nm/s; ratio (B→A/A→B); Metabolic stability Clint unit: μL/min/mg; Kinetic solubility (μg/mL); Cytotoxicity: % viability at 30 μM. “-”: not tested.

**Table 30:** Optimization of the P5 Group.

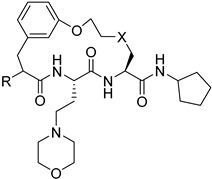												
		EC_50_ (µM)	β5 IC_50_ (µM)			Permeability		Metabo-lic Stability	Kinetic Solubility	Cytotoxicity HepG2	Lipopholicity	TPSA
ID	P3	3D7	Pf20S	c20S	i20S	PAMPA	MDR	m/hLM	pH 6.8	Viability %	cLogP	
TDI8304	-	0.016	0.081	>100	>100	15	>2/<1	6 19	>130	103	2.4	129.3
**249**	Gemdime-thyl	0.096	0.21	45.2	>100	269	35/11	690 167	90	97	3.7	109
**271**	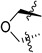	0.034	0.56	31.1	>100	89	>28/<1	5 4	68	102	2.4	118
**273**	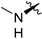	0.32	4.9	>100	>100	0	>2/<1	−14 −26	>140	100	2.2	121
**248**	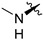	0.7	3.9	>100	>100	20	1.2/5	−1 15	>130	104	2.8	99.8
**270**	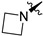	0.32	2.0	13.1	17.6	0	>2/<1	−27 6	>140	100	2.9	112.2

PAMPA unit: nm/s; MDR unit: A→B, nm/s; ratio (B→A/A→B); Metabolic stability Clint unit: μL/min/mg; Kinetic solubility (μg/mL); Cytotoxicity: % viability at 30 μM. “-”: not tested.

**Table 31:** Falcitidin acylated analogs and their IC_50_ values against *P. falciparum* 3D7 in a whole-cell assay (standardized with commercial chloroquine; IC_50_ 0.020 ± 0.002 µM).

Compound	IC_50_ (µM)
**296**	1.6
**297**	1.2
**298**	1.4
**299**	>10
**300**	1.3
**301**	3.3
**302**	0.14
**303**	2.4

**Table 32:** Inhibiton of FP-2, FP-3, and *P. falciparum* by gallinamide A analogs **309**–**312** (*n* = 3).

Analogue	IC_50_ FP-2 [nM]	IC_50_ FP-3 [nM]	IC_50_ *P. falciparum* (3D7) [nM]
**309**	6.78 ± 0.44	292 ± 1	8.93 ± 73
**310**	2.81 ± 0.39	163 ± 21	210 ± 80
**311**	3710 ± 420	>50,000	4375 ± 690
**312**	>50,000	>50,000	>50,000

**Table 33:** Inhibition of FP-2, FP-3, and the 3D7 Strain of *P. falciparum* by gallinamide A analogs **313**–**318** ^a^.

Analogue	IC_50_ FP-2 [nM]	IC_50_ FP-3 [nM]	IC_50_ *P. falciparum* (3D7) [nM]
**313**	10,500 ± 640	>25,000	540 ± 210
**314**	7700 ± 190	>50,000	1900 ± 620
**315**	11,500 ± 190	>25,000	320 ± 10
**316**	2500 ± 150	34,000 ± 460	1100 ± 800
**317**	3400 ± 390	>25,000	5400 ± 380
**318**	6000 ± 160	46,000 ± 5400	6600 ± 3800

^a^ Errors are standard error of the mean of three experiments.

**Table 34:** Inhibition of FP-2, FP-3, and the 3D7 strain of *P. falciparum* by gallinamide A analogs **309** and **346**–**351** ^a^.

Analogue	IC_50_ FP-2 [nM]	IC_50_ FP-3 [nM]	IC_50_ *P. falciparum* (3D7) [nM]
**309**	6.78 ± 0.44	292 ± 1	89.3 ± 73
**346**	24.8 ± 23	225 ± 27	57.3 ± 26
**347**	9.52 ± 0.14	131 ± 44	16.6 ± 9
**348**	5.25 ± 2	81.4 ± 8	20.0 ± 8
**349**	12.0 ± 3	66.7 ± 25	9.7 ± 2
**350**	9.59 ± 0.21	196 ± 7	96.0 ± 74
**351**	6.86 ± 3	182 ± 16	62.0 ± 47
CQ			17.3 ± 3

^a^ Errors are standard error of the mean of three experiments.

**Table 35:** Inhibition of the CQ-Resistant Dd2 Strain of *P. falciparum*, HEK298 Cells, and *P. falciparum* aminopeptidase M1, M17, and M18 (AP M1, M17, and M18) by gallinamide A analogs ^a^.

Analog	IC_50_ *P. falciparum* (Dd2) [nM]	HEK298 [nM]	AP M1, M17, and M18 [nM]
**309**	302 ± 126	14 200 ± 1050	>10,000
**313**	421 ± 2	>50,000	>10,000
**315**	378 ± 20	>50,000	>10,000
**317**	170 ± 68	>50,000	>10,000
**346**	419 ± 185	16,300 ± 1700	>10,000
**347**	165 ± 68	9650 ± 1480	>10,000
**348**	67.0 ± 30	18,900 ± 110	>10,000
**349**	29.0 ± 16	8500 ± 124	>10,000

^a^ Errors are standard error of the mean of three experiments.

**Table 36:** Inhibition of FP2, FP3 and 3D7 and W2 Strains of *P. falciparum* ^a^.

Compound	IC_50_ FP-2 [nM]	IC_50_ FP-3 [nM]	IC_50_ 3D7 [nM]	IC_50_ W2 [nM]
**352**	12	67	9.7	ND
**371**	59	131	14	11
**372**	31	117	26	28
**373**	29	79	42	49
**374**	60	851	24	10
**375**	57	228	5	7
**376**	3097	10,235	2593	691
**377**	3523	8788	1248	478
**378**	464	459	229	119
**379**	235	407	72	37
**380**	455	547	164	55
**381**	156	480	205	59
**382**	>50,000	>50,000	>10,000	3729
**383**	>50,000	>50,000	6994	1017
**384**	84	484	9	10
**385**	169	490	83	95
**386**	270	2571	76	54
**387**	33	112	1	4
CQ	ND	ND	4	55
ART	ND	ND	32	21
E64	68	136	ND	ND

^a^ CQ = cholorquine, ART = artemisin, E64 = proteinase inhibitor E64. ND = not determined.

**Table 37:** In Vitro degradation half-life in mouse blood and plasma.

	Degradation Half-Life (min)					
	352	372	373	375	384	387
plasma	<2	126	82	557	>600	373
blood	<2	41	17	110	>600	273

**Table 38:** The shared percentage volume of each peptide and Suc-ILVY-AMC substrate according to the poses and conformations selected in the docking studies.

Peptides	Act > 50%	Shared Volume %
SE5 P1 (**388**)	1	50.1
SE5 P2 (**389**)	1	57.5
SE5 P3 (**390**)	0	27.9
SE5 P4 (**391**)	0	41.6
SE5 P5 (**392**)	0	19.9
SE5 P6 (**393**)	0	43.5
SE5 P7 (**394**)	0	45.1
SE5 P8 (**395**)	0	40.2
SE5 P9 (**396**)	0	39.2

**Table 39:** Therapeutic index determination (MHC/MIC ratio).

	**MHC [M]**	**MIC [M]**	**MHC/MIC**
Ang II	10^−5^	10^−12^	10^7^
Peptide **401**	10^−5^	10^−14^	10^9^
Peptide **402**	10^−6^	10^−14^	10^8^

Therapeutic index was determined for the compounds that had anti-plasmodial activity during the erythrocytic cycle of *P. falciparum*.

## Data Availability

Not Applicable.
